# Integrated multiomics analysis identifies PHLDA1+ fibroblasts as prognostic biomarkers and mediators of biological functions in pancreatic cancer

**DOI:** 10.3389/fimmu.2025.1592416

**Published:** 2025-07-04

**Authors:** Rui Wang, Guan-Hua Qin, Yifei Jiang, Fu-Xiang Chen, Zi-Han Wang, Lin-Ling Ju, Lin Chen, Da Fu, En-Yu Liu, Su-Qing Zhang, Wei-Hua Cai

**Affiliations:** ^1^ Department of Hepatobiliary Surgery, Affiliated Nantong Hospital 3 of Nantong University, Nantong, Jiangsu, China; ^2^ Medical School of Nantong University, Nantong, Jiangsu, China; ^3^ Department of Hepatobiliary and Pancreatic Surgery, Affiliated Tumor Hospital of Nantong University, Nantong, Jiangsu, China; ^4^ Department of Nuclear Medicine, Ruijin Hospital, Shanghai Jiaotong University School of Medicine, Shanghai, China; ^5^ Nantong Institute of Liver Disease, Affiliated Nantong Hospital 3 of Nantong University, Nantong, Jiangsu, China; ^6^ Department of General Surgery, Ruijin Hospital, Shanghai Jiaotong University School of Medicine, Shanghai, China; ^7^ Department of General Surgery, Qilu Hospital, Cheeloo College of Medicine, Shandong University, Jinan, Shandong, China

**Keywords:** pancreatic cancer, PHLDA1, prognostic biomarker, tumor microenvironment (TME), spatial transcriptomics

## Abstract

**Background:**

Pancreatic cancer (PC) is marked by extensive heterogeneity, posing significant challenges to effective treatment. The tumor microenvironment (TME), particularly cancer-associated fibroblasts (CAFs), plays a critical role in driving PC progression. However, the prognostic and functional contributions of distinct CAF subtypes remain inadequately understood. Here, we introduce a novel 7-gene risk model that not only robustly stratifies PC patients but also unveils the unique role of PHLDA1 as a key mediator in tumor-stroma crosstalk.

**Methods:**

By integrating single-cell RNA sequencing (scRNA-seq), spatial transcriptomics, and bulk RNA sequencing data, we comprehensively characterized the heterogeneity of CAFs in PC. We identified five CAF subtypes and focused on matrix CAFs (mCAFs), which were strongly associated with poor prognosis. A 7-gene mCAF-associated risk model was constructed using advanced machine learning algorithms, and the biological significance of PHLDA1 was validated through co-culture experiments and pan-cancer analyses.

**Results:**

Our multiomics analysis revealed that the novel 7-gene model (comprising USP36, KLF5, MT2A, KDM6B, PHLDA1, REL, and DDIT4) accurately predicts patient survival, immunotherapy response, and TME status. Notably, PHLDA1 was uniquely overexpressed in CAFs and correlated with the activation of key protumorigenic pathways, including EMT, KRAS, and TGF-β, underscoring its central role in modulating the crosstalk between CAFs and malignant ductal cells. Pan-cancer analysis further supported PHLDA1’s prognostic and immunomodulatory significance across multiple tumor types.

**Conclusion:**

Our study presents a novel 7-gene prognostic model that significantly enhances risk stratification in PC and identifies PHLDA1+ CAFs as promising prognostic biomarkers and therapeutic targets. These findings provide new insights into the TME of PC and open avenues for personalized treatment strategies.

## Background

Pancreatic cancer (PC) is an aggressive neoplasm of the digestive system and may be expected to emerge as the major frequent cause of cancer-related fatality by 2030 (1). Despite the incremental progress in diagnostic modalities and therapeutic strategies, the overall survival (OS) rate for PC continues strikingly low, at less than 10% (2). This grim prognosis is attributed to the fact that over 80% patients preclude the possibility of curative surgery and increases the risk of tumor recurrence (3). For patients with unresectable PC, chemotherapy regimens based on fluorouracil or gemcitabine, has shown limited efficacy, with survival extension not exceeding 12 months (4–6). Therefore, to improve clinical outcomes, there is an imperative need to elucidate the intricate biological underpinnings of pancreatic cancer cells and their associated cellular milieu comprehensively.

The tumor microenvironment (TME) has recently assumed a central focus on oncological research and drug development, encompassing a diverse array of cellular and noncellular elements, comprising immune cells, cancer-associated fibroblasts (CAFs) or cytokines (7–9). The intricate interplay within the TME is pivotal in modulating malignancy progression (10). CAFs, a predominant cell type in the stromal constituents, is closely associated with invasion, metastasis, or poor prognosis in a variety of malignant tumors (11, 12). Single-cell analyses have revealed distinct CAF subtypes, each characterized by unique genetic signatures or functional attributes. The heterogeneity of fibroblasts has been investigated across various cancers, including colorectal (13, 14), chordoma (15), breast (16, 17), and head and neck cancer (18), among others. The variability in CAF types and functions across different tumor types highlights the complexity of their functional role within the TME, indicating a need for further investigation into their multifaceted contributions. Given the marked heterogeneity within the CAF population, we hypothesize that distinct CAF subtypes exert unique influences on pancreatic cancer progression. In particular, we postulate that a specific subset characterized by elevated PHLDA1 expression plays a pivotal role in mediating the crosstalk between malignant ductal cells and the tumor microenvironment. We propose that PHLDA1+ CAFs contribute to tumor growth and immune modulation by activating protumorigenic signaling pathways—such as PI3K/Akt, TGF-β, and KRAS—which, in turn, may impact patient prognosis and therapeutic response. This hypothesis underpins our investigation into the prognostic and functional significance of PHLDA1+ CAFs in pancreatic cancer.

In recent years, machine learning (ML) approaches have become indispensable for extracting robust prognostic and biological insights from high‐dimensional cancer datasets (19). Supervised methods—such as Lasso‐Cox regression, Random Forests, and Support Vector Machines—have been widely applied to bulk and single‐cell transcriptomic profiles to derive multi‐gene signatures that accurately stratify patients by survival risk and therapeutic response. Unsupervised algorithms, including consensus clustering and non‐negative matrix factorization, have facilitated the identification of novel TME cellular subtypes by grouping cells with shared expression patterns, thereby revealing heterogeneity that is otherwise obscured in bulk analyses (20). More recently, deep learning frameworks have been integrated with spatial transcriptomics to infer spatially resolved cell–cell interactions, enabling the construction of predictive models that link the spatial distribution of immune and stromal populations to clinical outcomes (21). In pancreatic cancer and other malignancies, such integrative ML pipelines have successfully uncovered prognostic signatures within CAFs, predicted immunotherapy responders based on TME composition, and highlighted key signaling pathways driving tumor–stroma crosstalk. By leveraging these advanced algorithms, our study not only constructs a robust 7‐gene risk model but also situates PHLDA1+ CAFs within a framework of ML‐driven TME analysis, underlining their relevance for precision prognostication and therapeutic targeting.

ScRNA-seq technology has enabled the characterization of tumor cell heterogeneity with single-cell resolution, thereby laying a more robust foundation for the comprehensive elucidation of tumor pathogenesis, therapeutic strategies, and prognostic outcomes (22, 23). Spatial transcriptomics (ST) methodologies facilitate the acquisition of whole-transcriptome data within tissue sections, concurrently preserving the spatial context of cellular localization (24).

In this research, multi-omics data were used to elucidate the contributions of CAFs in the malignant progression from a multidimensional perspective. Furthermore, we sought to investigate the influence of CAFs on the prognosis of patients with PC and their possible predictive value for the response to immunotherapy. Our research contributes to clarifying the biological roles of CAFs in the development of PC and offers guidance for creating innovative treatment approaches.

## Methods

### Data collection

All data were obtained from GEO database (https://www.ncbi.nlm.nih.gov/geo/) and Xena database (https://xena.ucsc.edu/). ScRNA data consisted of GSE154778, GSE155698, and GSE231535 datasets, comprising 38 samples of primary pancreatic cancer and control tissues (25–27). Spatial transcriptomic data were derived from the GSE235315 dataset, used for deconvolution of single-cell data to observe cell type distribution (28). The bulk datasets were divided into three parts: 1. The dataset for training the prognostic model was sourced from the TCGA-PAAD cohort, including 176 pancreatic cancer patients with survival and clinical information. 2. The datasets for validating the prognostic model were obtained from the GSE28735, GSE57495, and GSE62452 datasets, all containing survival information for pancreatic cancer patients (29–31). 3. The dataset for expression analysis was created by batch-correcting and merging the TCGA-PAAD cohort with the GTEX pancreatic cohort to increase the number of control samples, totaling 176 pancreatic cancer tissues and 167 control pancreatic tissues.

### Data processing

For single-cell data, analyses were conducted using Seurat 4.2.2. Data were normalized for dimensionality reduction and clustering. The Harmony algorithm was employed to correct batch effects across datasets and samples. Cell annotation was performed using SingleR and existing methods. The percentage of cells was displayed using the “ggalluvial” software package after identifying marker genes for cell types.

For spatial transcriptomic data, the “cell2location” package was installed in a Python 3.9 environment for analysis. The “scanpy” package was used to import spatial transcriptomic data, filtering out low-quality cells after removing mitochondrial genes. A negative binomial regression model was used to train a feature matrix from single-cell data, achieving optimal results with max_epochs set to 250. Shared genes between single-cell and spatial data were identified as reference signatures for deconvolution analysis, predicting cell abundance. Considering the availability of data and code, we supplemented the analyzed relevant code with Seurat objects to the supplemental notebook.

For bulk transcriptomic data, the “sva” package facilitated batch correction and merging of TCGA and GTEX data. The data were then analyzed for different expression and survival.

### Identification of CAF subtypes

To define and annotate cancer-associated fibroblast (CAF) subtypes, we first performed dimensionality reduction and unsupervised clustering within the CAF across all three scRNA-seq cohorts (GSE154778, GSE155698, GSE231535). Clustering was conducted in Seurat v4.2.2 using principal component analysis (PCA) followed by the Louvain algorithm (resolution = 0.6). Marker genes for each cluster were identified with FindMarkers (log_2_ fold change > 0.25, adjusted P < 0.05). We required that each putative CAF subtype exhibit at least five independently validated “signature” genes (e.g., FAP, POSTN, COL1A1 for matrix CAFs (mCAFs); CXCL1, IL6, CXCL12 for iCAFs) with significant overexpression relative to other fibroblast clusters.

To assess consistency across datasets, we reclustered CAFs independently in each cohort under identical parameters (Harmony for batch correction, followed by Louvain clustering). Each of the five subtypes (iCAFs, proCAFs, mCAFs, MT2A^+^ myCAFs, CXCL14^+^ myCAFs) appeared in all three cohorts, and the adjusted Rand index (ARI) between integrated and per-dataset cluster assignments exceeded 0.85 in each case. Finally, to verify that subtype definitions were not an artifact of a single clustering technique, we repeated the CAF subtyping using a Leiden algorithm (resolution = 0.5) and hierarchical clustering on z-score–normalized expression profiles; subtype identities and relative proportions differed by less than 5% compared to the Louvain result.

### Identification of malignant versus non-malignant cells

First, we sorted the input expression matrix according to the order of genes in the genome, followed by data normalization. The cells were then clustered based on Euclidean distance or correlation. A Gaussian mixture model (GMM) was used to estimate the variance of each cluster, with the cluster showing the least variance serving as the diploid reference (i.e., normal cells) for subsequent analysis. When calculating copy number alterations (CNA) through gene expression, CopyKAT grouped every 25 genes into a detection window and assessed the significance of the mean expression differences between adjacent windows. Windows with significant differences were identified as chromosomal breakpoints. Finally, hierarchical clustering using CNA data was performed to distinguish between aneuploid tumor cells and diploid normal cells. This process was carried out using the R package “CopyKAT”.

### Inference of cell–cell communication networks

The underlying mechanisms of cell-to-cell communication were uncovered by the “CellChat” v1.5.0. The netVisual_circle function visualized the number and strength of communications between cells, whereas the netAnalysis_computeCentrality function inferred the input and output weights of specific signaling pathways.

### Estimation of cell-type proportions in TCGA-PAAD cohort

The deconvolution algorithm extracted representative features from high-dimensional data and mapped them to a lower-dimensional space to identify the proportions of elements in the high-dimensional data. To perform deconvolution, the “IOBR” package was used. First, the generateRef_seurat function extracted feature genes from single-cell data to construct a deconvolution feature expression matrix. The deconvo_tme function then applied the SVR algorithm to deconvolve the abundance of all cell types in the TCGA-PAAD dataset.

### Identification of core gene modules through high-dimensional co-expression analysis

hdWGCNA (high-dimensional WGCNA) is a systems biology method that analyzes high-throughput gene expression data to uncover relationships between genes. Specifically, the SetupForWGCNA function constructs a WGCNA object, and the MetacellsByGroups function creates metacell information. Gene module analysis was performed based on the soft threshold of the co-expression network, and module eigengenes were calculated to identify core genes.

### Machine learning and prognostic model construction

To develop a prognostic risk model, ten machine learning methods were used for selection and modeling: Lasso, Enet, StepCox, SurvivalSVM, CoxBoost, SuperPC, Ridge, plsRcox, RSF, and GBM. These were combined in various ways to create 101 different algorithms to assess the diagnostic efficiency of the models.

### Cross-validation and model selection

To mitigate the risk of overfitting associated with testing 101 algorithms, we implemented a rigorous 10-fold cross-validation procedure on the training set. Specifically, the TCGA-PAAD cohort was randomly partitioned into 10 equal subsets. In each iteration, 9 folds were used to train the model, while the remaining fold served as the validation set. We computed performance metrics, including the concordance index (c-index) and the area under the receiver operating characteristic curve (AUC), for each fold. The final model was selected based on the highest average performance across the 10 folds. Additionally, sensitivity analyses were performed to assess the stability of model parameters. The selected model was further validated using independent external datasets to ensure its generalizability.

### Immune infiltration and immunotherapy assessment

To analyze the overall immune microenvironment and potential for immunotherapy in high- and low-risk patients, the CIBERSORT algorithm was performed. Additionally, the expression of key factors such as chemokines, TNF family factors and HLA family molecules in high- and low-risk groups was further investigated. Overall activation of the immune microenvironment was assessed by the “ESTIMATE” package.

The “IOBR” package was used for evaluating tumor TME-related gene sets. The TIDE website (http://tide.dfci.harvard.edu/) was subsequently performed to analyze immunoreactivity and to assess immunotherapy sensitivity based on factors, such as co-mutation frequency, tumor mutation burden, and immune checkpoint expression. Finally, the external immunotherapy datasets IMvigor210 and GSE91061 were used for validation (32).

### Enrichment analysis

All genes were ranked by logFC values. HALLMARK enrichment analysis was conducted using the “GSEA” and “clusterProfiler” packages, with a significance threshold of adjusted *P* < 0.05.

### Drug screening and molecular docking

Drug screening was primarily conducted using the DSigDB database available on the Enrichr website. Drugs with an adjusted *P* < 0.01 were selected. The top 20 drugs were selected for display according to the binding score. The top1 drug was chosen for further analysis. For molecular docking, AutoDock Tools 1.5.6 was used to set charges, add polar and nonpolar hydrogens, and define rotatable bonds. The receptor grid files were generated by AutoDock Tools. AutoDock Vina 1.2.5 was then employed to dock the ligand structures with the generated receptor grid files. The results were visualized, analyzed, and plotted using PyMOL 3.2.

### Pan-cancer analysis

Pan-cancer data were derived in accordance with UCSC Xena (https://xenabrowser.net), encompassing 24 tumor types from TCGA. The “Limma” package was used for uniform standardization and normalization of all datasets. Survival analysis was then performed. Gene sets related to angiogenesis, cell cycle, and EMT were sourced from previous studies (33–35). Correlation scatter plots were created using “ggplot2”.

### Clinical samples

Tumor tissues and paired adjacent tissues were obtained from Ruijin Hospital, Shanghai Jiao Tong University School of Medicine. The detail patient clinicopathologic information can be viewed in **Supplementary Table S1**. The research protocol was approved by the Research Ethics Committee of Ruijin Hospital, Shanghai Jiao Tong University School of Medicine. All the participants agreed to participate in this cohort study and provided written informed consent.

### Cell culture and transfection

PATU-8988 and PANC-1 were purchased from the Cell Bank of the Chinese Academy of Sciences. Cancer-associated fibroblasts (CAFs) were obtained from the tumor tissues. The above were cultured in RPMI-1640 medium containing 10% fetal bovine serum (FBS) and 1% penicillin/streptomycin (P/S). The incubation temperature was 37°C and the incubator was 5% CO_2_. Short hairpin RNA of PHLDA1 was provided by Genechem (Shanghai, China). For transfections, proper plasmids were introduced into the supernatant using HilyMax (Dojindo,Japan). After 8–12 hours, the medium was replaced and then validated.

### Immunohistochemistry and immunofluorescence

After being formalin-fixed and paraffin-embedded, the tumor tissue samples were sectioned onto slides. IHC was then performed to validate the expression of PHLDA1.

Following deparaffinization and rehydration, the slides were subjected to antigen repair. This was subsequently followed by antibody incubation, color development and sealing. Finally, representative images were captured under a microscope. Similar to the IHC protocol, IF was carried out.

### RNA extraction and real-time quantitative PCR

Total RNA was abstracted by TRIzol reagent (Invitrogen, USA). cDNA was gotten by reverse transcription using HiScript III RT SuperMix (Vazyme, Nanjing, China). RT-qPCR was performed with the ChamQ SYBR qPCR Master Mix (Vazyme Biotech, China) according to the manufacturer’s instructions.

### Patient-derived organoid construction and evaluation

Pancreatic tumor tissues from patients were quickly separated in RPMI-1640 medium that had been chilled beforehand and digested for 30 minutes at 37°C using collagenase. Individual cells were subsequently placed into Matrigel (Corning, USA) and filtered through a Falcon 40 μm cell screen (Corning, USA). They were then grown in the full organoid medium (OmaStem, China). Following the manufacturer’s instructions, the CellTiter-Glo 3D cell viability assay (Promega, USA) was used to measure the relative activity of the organoids after being co-cultured with CAFs.

### Western blot

RIPA buffer (Epizyme, China) combined with protease inhibitors (Epizyme, China) was used to extract proteins from cells. Separated from 10% SDS-PAGE, proteins were transferred to PVDF membranes. PHLDA1 primary antibody (Abcam, UK) and the relevant secondary antibody were used for incubation. Finally, protein expression levels were determined using ECL reagents (Epizyme, China).

### Cell proliferation assay

For the Cell Counting Kit-8 (CCK-8; MeilunBio, China) assay, 2000 cells were plated in a 96-well plate and then cultivated at 37°C in an incubator with 5% CO2. Following the addition of 90 μL of growth media and 10 μL of CCK-8 to each well at specified times, the cells were cultured for an additional 2 hours, and the optical density (OD) values of each group were measured at 450 nm. For colony formation, the cells were seeded at a density of 1000 cells per well in a 6-well plate, and the medium was replaced every three days. The cells were subsequently fixed and stained with 0.1% crystal violet at room temperature for 30 minutes. ImageJ software was used to quantify the number of colonies after they were imaged.

### Cell migration assay

For the migration assay, the upper chamber was filled with pancreatic cancer cells (5 × 10^4^) suspended in 200 µL of serum-free media, while 1×10^6^ CAFs were seeded in lower chamber culture plates containing 700 μl of RPMI-1640 medium supplemented with 10% FBS. After 24 hours, the cells that had moved to the lower side of the membrane were fixed and stained for 15 minutes at room temperature with a 1% crystal violet solution. ImageJ software was used to count the number of moving cells.

### Statistical analysis

Statistical analyses were performed using GraphPad Prism 9.0. Continuous variables are presented as mean ± standard deviation (SD), and their distribution was assessed by the Shapiro–Wilk test. For comparisons between two independent groups, an unpaired two-tailed Student’s t-test was applied when data were normally distributed; otherwise, the Mann–Whitney U test was used. For comparisons among three or more independent groups, one-way analysis of variance (ANOVA) followed by Tukey’s multiple comparisons test was employed. Categorical variables were compared using Pearson’s chi-square test; when any expected cell count was less than 5, Fisher’s exact test was used instead. Survival curves were generated by the Kaplan–Meier method, and differences between survival curves were evaluated by the log-rank test. All statistical tests were two-tailed, and a P value < 0.05 was considered statistically significant.

## Results

### Characterization of the single-cell landscape in pancreatic cancer

**Figure 1:**
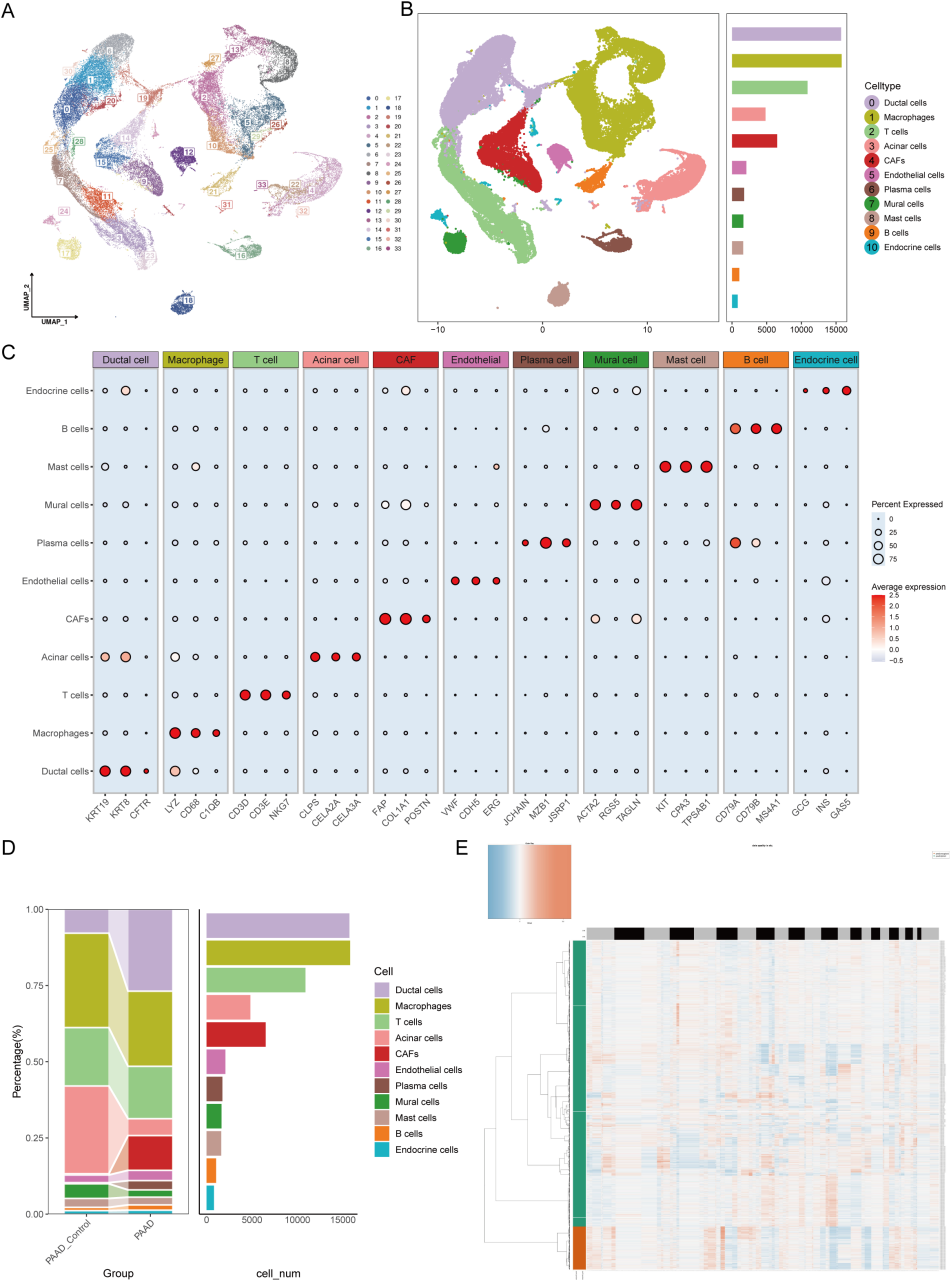
Single-cell atlas features of pancreatic cancer construction. **(A)** UMAP visualization analysis of 56,853 cells from 34 clusters by integrating the GSE154778, GSE155698 and GSE231535 datasets. **(B)** The number of each cell type in the integrated dataset. **(C)** Percentages and abundances of marker genes expressed in different cell types. The horizontal axis represents marker genes in different cell types, and the vertical axis represents different cell subpopulations. The size of the dots represents the percentage of expression, and the color shading represents the average expression level. **(D)** Histograms of the percentages and numbers of various types of cells in normal and pancreatic tumor tissues. **(E)** The CopyKAT algorithm suggested that most ductal cells were malignant.

Based on methodological quality control standards, we retained 5,805 normal control group cells and 56,853 pancreatic cancer cells for downstream analysis. These included 12,458 from GSE154778, 37,583 from GSE155698, and 12,617 from GSE231535. After batch effect removal, 34 clusters of cells labeled 0–33 were identified (**Figure 1A**). Using SingleR and existing methods, we annotated these 34 clusters into 11 cell types. The markers for each cell type included ductal cells (KRT19, KRT8, and CFTR), macrophages (LYZ, CD68, and C1QB), T cells (CD3D, CD3E, and NKG7), acinar cells (CLPS, CELA2A, and CELA3A), cancer-associated fibroblasts (FAP, COL1A1, and POSTN), endothelial cells (VWF, CDH5, and ERG), plasma cells (JCHAIN, MZB1, and JSRP1), pericytes (ACTA2, RGS5, and TAGLN), mast cells (KIT, CPA3, and TPSAB1), B cells (CD79A, CD79B, and MS4A1), and endocrine cells (GCG, INS, and GAS5) (**Figures 1B, C**). The changes in each cell type of consistency were compared and we found that proportions of ductal cells, CAFs, and plasma cells increased, whereas those of acinar cells and pericytes decreased. These findings suggested that ductal cells, CAFs, and plasma cells may be associated with the malignant progression (**Figure 1D**). Additionally, we found that most malignant cells originated from ductal cells using the CopyKAT (**Figure 1E**). This raised the question of whether malignant ductal cells have significant biological differences from nonmalignant ductal cells, accelerating progression of pancreatic cancer.

### Crosstalk between cancer-associated fibroblasts and malignant ductal cells

**Figure 2:**
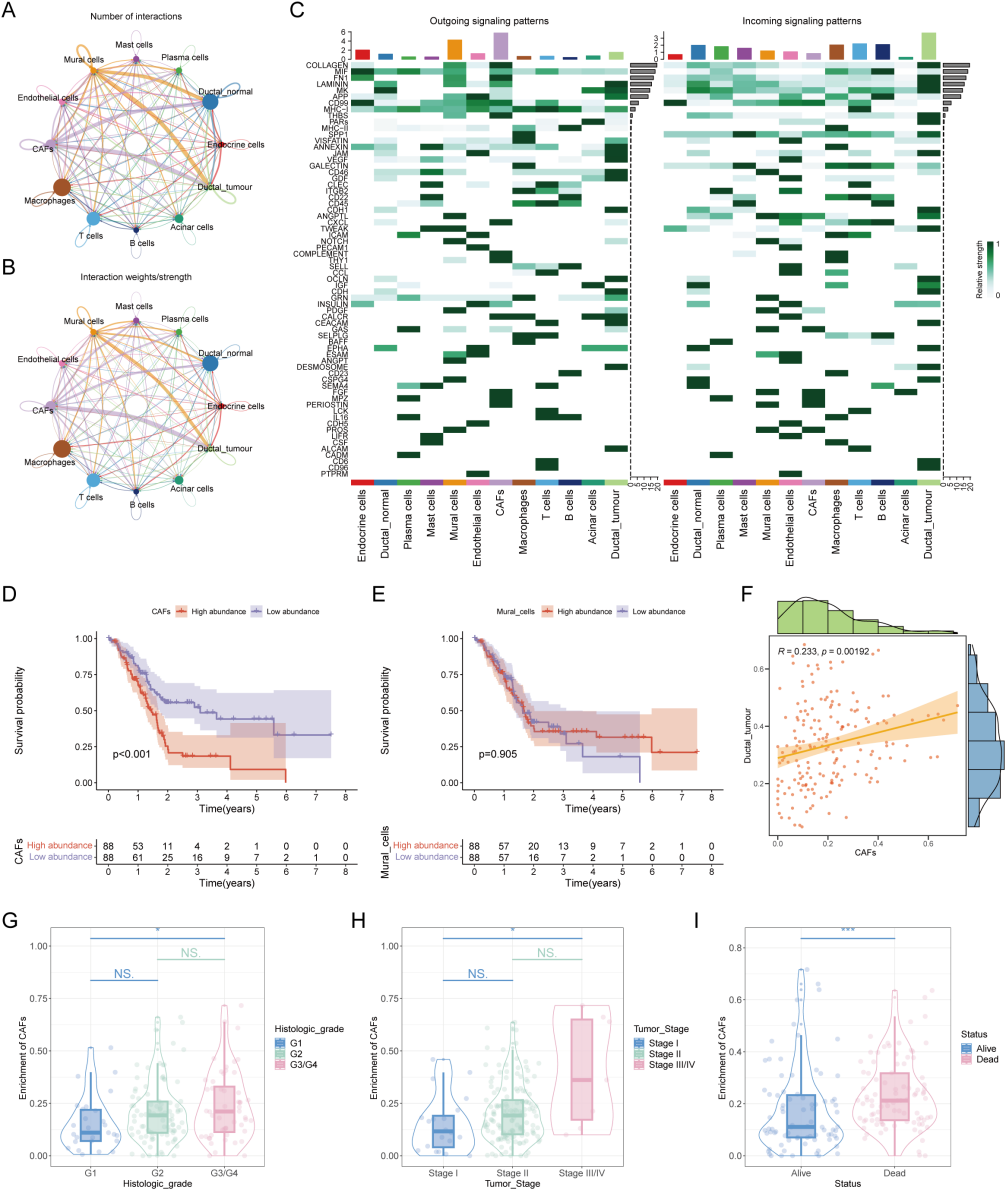
In-depth analysis of mutual crosstalk between ductal cells and cancer-associated fibroblasts. **(A)** Number of interactions between different cell subpopulations. **(B)** Interaction weights/strengths among each cell type. **(C)** Outgoing signaling patterns and incoming signaling patterns of all cell types. **(D)** Survival analysis revealed that the abundance of CAFs was correlated with poor prognosis in patients with pancreatic cancer. **(E)** KM plot showing the relationship between the abundance of mural cells and patient prognosis. **(F)** Correlation analysis of the abundance between CAFs and malignant ductal cells. **(G)** The abundance of CAFs was correlated with clinical stage. **(H)** A positive association was found between the number of CAFs and the pathological stage of patients. **(I)** There was an association between the number of CAFs and the survival status of patients. * represents p<0.05; *** represents p<0.001.

To address this, we divided the ductal cells in the single-cell data into tumor-associated ductal cells and normal ductal cells based on whether they were diploid and calculated their communication with other cell components in the microenvironment. The results revealed that CAF or pericytes were most closely interconnected with malignant ductal cells (**Figures 2A, B**). In terms of specific communication signals, tumor-associated ductal cells increase the output of signals such as ALCAM and OCLN and the input of signals such as CD96 and CD6 (**Figure 2C**). To explore whether CAFs or pericytes play a more important role in promoting disease, we extracted a reference set of feature genes from the single-cell expression matrix and deconvoluted them into ordinary transcriptome data. We found that the abundance of CAFs was significantly related to overall survival, with a higher proportion of CAFs associated with worse overall survival (**Figures 2D, E**). In addition, CAFs had a substantial positive correlation with the quantity of tumor-associated ductal cells, suggesting that CAFs may be the most important cells involved in malignant progression via affecting ductal cells (**Figure 2F**). At the same time, CAFs were also related to clinical stage, with worse stages such as G3/G4 or Stage III/IV having a higher proportion of CAFs (**Figures 2G–I**). Elucidating the characteristics and functions of CAFs may be important for understanding pancreatic cancer.

### mCAFs correlate with clinical prognosis in pancreatic cancer

**Figure 3:**
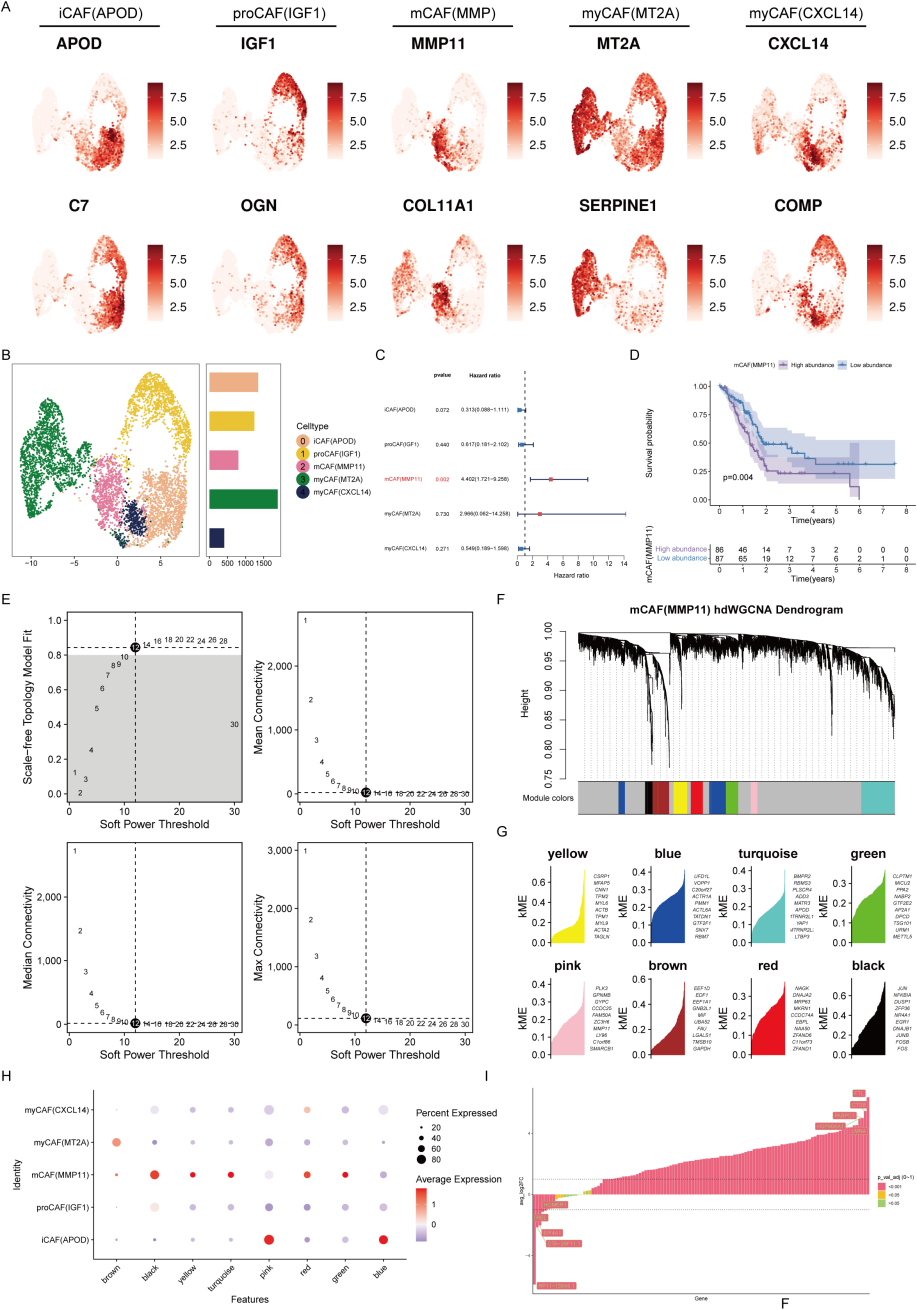
mCAFs are associated with poor prognosis in pancreatic cancer patients. **(A)** Expression levels of marker genes for 5 different CAF subtypes. The color shading represents the intensity of expression. **(B)** UMAP plot demonstrating the distribution of the five different CAF subtypes. Histograms indicate the number of cells in each type of subpopulation. **(C)** Univariate Cox analysis to assess the impact of five different CAF subtypes on the prognosis of patients with pancreatic cancer. **(D)** Survival analysis revealed that the abundance of mCAFs was correlated with poor prognosis in patients with pancreatic cancer. **(E)** The selection of the optimal soft threshold. **(F)** Scale-free topological network models were built using an ideal soft threshold of 12, and genes were partitioned into modules to create gene clustering trees. **(G)** The feature-based gene connectivity for each gene in the scale-free topological network analysis was calculated to determine the highly connected genes in each module. **(H)** Bubble plots illustrating the associations of different color modular genes with different CAF isoforms, with the black module gene having the highest correlation with mCAFs. **(I)** Differential expression analysis of black module genes.

Next, by secondary dimensionality reduction and clustering on the CAFs, and based on their respective expression characteristics, five cell subgroups were derived: inflammatory CAFs (iCAFs), progenitor CAFs (proCAFs), matrix CAFs (mCAFs), and myogenic CAFs (two subtypes with high expression of MT2A and CXCL14 respectively) (**Figures 3A, B**). Univariate Cox analysis revealed that among these five types of cells, only mCAFs had a significant correlation with overall survival. The survival curve also revealed that a greater abundance of mCAFs was related to worse prognosis, suggesting mCAFs constituted the most significant malignant CAF subtype (**Figures 3C, D**). HdWGCNA is an important means to mine core genes. To parse the characteristic genes of mCAFs, hdWGCNA was performed. Given a soft threshold of 12, we achieved the best attributes of the scale-free topological network model and good connectivity (**Figure 3E**). At this time, all genes were divided into eight color series module genes, including yellow, blue, turquoise, green, pink, brown, red, and black modules (**Figures 3F, G**). By calculating the correlation of each module gene with different CAF subtypes, we found that the black module genes had the highest correlation with mCAFs, indicating that the module genes most closely fit the characteristics of mCAFs (**Figure 3H**). This module gene has a total of 125 genes. Through differential expression analysis, we retained genes whose expression significantly differed, which will be used as candidate genes for the next step of core prognostic gene screening. (**Figure 3I**).

### Performance of a prognostic model

**Figure 4:**
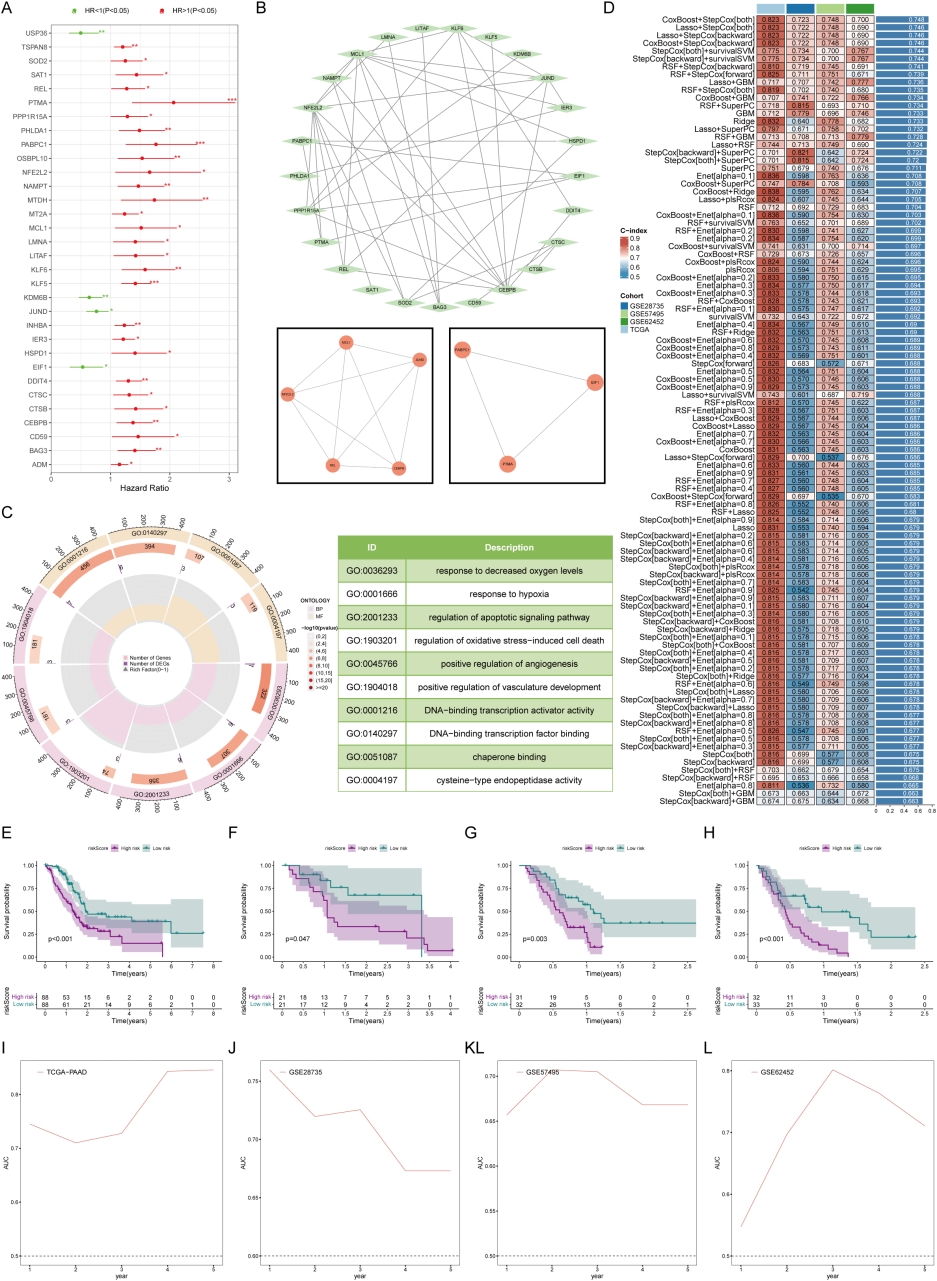
Establishment and testing of the prognostic model. **(A)** Univariate Cox analysis revealed 32 marker genes associated with survival in patients with pancreatic cancer. **(B)** Network map of protein interactions of 32 survival-related genes. **(C)** Gene ontology enrichment analysis suggested that survival-related mCAF marker genes activated protumor-related biological processes. **(D)** 101 machine learning algorithms for marker gene screening and prognostic model construction. **(E–H)** Overall survival was compared between the high- and low-risk groups in K-M plots in both the training **(E)** and validation cohorts **(F–H)**. **(I–L)** Time-dependent ROC curves for estimating 1-, 3-, and 5-year overall survival in the training **(I)** and validation **(J–L)** cohorts. * represents p<0.05; ** represents p<0.01; *** represents p<0.001.

Before constructing a prognostic model, we prescreened survival for the candidate genes. By Univariate Cox regression analysis, a total of 32 genes were substantial correlated with overall survival (**Figure 4A**). We constructed a protein interaction network of these 32 genes, and the MCODE algorithm extracted two core submodules from it (**Figure 4B**). Enrichment analysis revealed that these survival-related mCAF characteristic genes were enriched mainly in processes promoting tumors, such as hypoxia, angiogenesis, and apoptosis (**Figure 4C**). The results of feature gene screening and model construction based on 101 survival analyses revealed that after CoxBoost was used for core gene screening and StepCox was used for prognostic model construction, the best prognostic model was obtained. The average concordance index (C-index) reached 0.748, indicating excellent predictive performance (**Figure 4D**). The genes involved in the construction of this prognostic model included USP36, KLF5, MT2A, KDM6B, PHLDA1, REL, and DDIT4. According to this prognostic model, pancreatic cancer patients were stratified by disease status. Patients in the low-risk group had a better prognosis than those in the high-risk group (**Figures 4E–H**). The model exhibited consistently robust predictive ability in both the training and validation cohorts, particularly at the second and third years of follow-up, where the area under the ROC curves (AUCs) exceeded 0.7 and even reached above 0.8(**Figures 4I–L**), suggesting that the reliability and accuracy of the constructed prognostic model.

### Immune atlas of high- and low-risk groups

**Figure 5:**
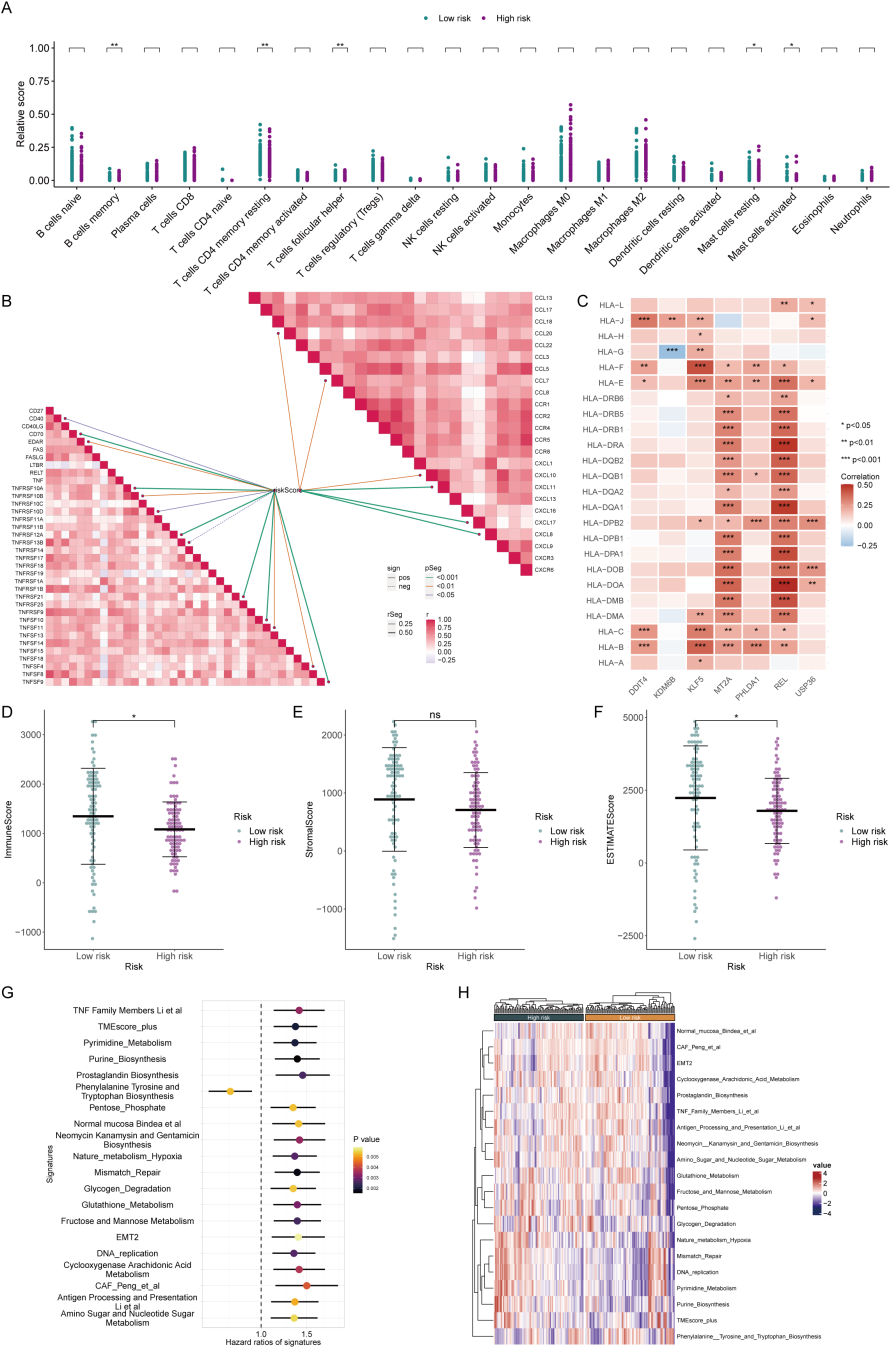
Assessment of the immune microenvironment in patients in high and low-risk groups. **(A)** The CIBERSORT algorithm demonstrated enrichment levels of various types of immune cells in patients from different risk groups. **(B)** Correlation analysis of different immunoreactive factors with the risk score. **(C)** Relationships of core genes with molecules associated with antigen presentation. **(D–F)** The ESTIMATE algorithm evaluated the ImmuneScore **(D)**, StromalScore **(E)**, and ESTIMATEScore **(F)** in patients from the high- and low-risk groups. **(G**, **H)** Assessment of hazard ratios (HRs) and activation levels for gene sets associated with the tumor microenvironment.

Furthermore, we assessed the immune status of different groups patients. CIBERSORT analysis revealed that high-risk patients had fewer immune activation-related cells, such as memory B cells and follicular helper T cells, but more mast cells. These findings suggested that high-risk patients may have certain defects in assisting the activation of adaptive immunity (**Figure 5A**). In terms of immune activity factors, chemokines and the TNF family are common cell factors that induce immune cell aggregation and activate inflammatory responses. The risk score was markedly positively corresponded to these two molecules, suggesting that high-risk patients experienced a high degree of cytokine storm, which strongly promoted continuous chronic progression and delay of tumor development (**Figure 5B**). The HLA family comprises common antigen-presenting-related molecules. Except for MT2A and REL in the prognostic model, which are significantly positively correlated with the HLA family, most molecules are unrelated or even negatively correlated with the HLA family. These findings suggest that there was no obvious antigen presentation activation in the high-risk group (**Figure 5C**). Microenvironment scoring revealed a more active immune status and a smaller proportion of tumor cells in the low-risk group. Conversely, patients in the high-risk group exhibited a state of immune deficiency and high tumor tissue infiltration (**Figures 5D–F**). In addition, we evaluated many gene sets related to the tumor microenvironment, including genes related to mismatch repair, EMT, and various biological metabolisms, all of which had positive risk scores (HRs). The high-risk group had higher CAF scores, EMT scores, etc., suggesting that the high-risk group was overall in a state of low antitumor immunity and an accelerated protumor environment (**Figures 5G, H**).

### Immunotherapy response assessment by risk score

**Figure 6:**
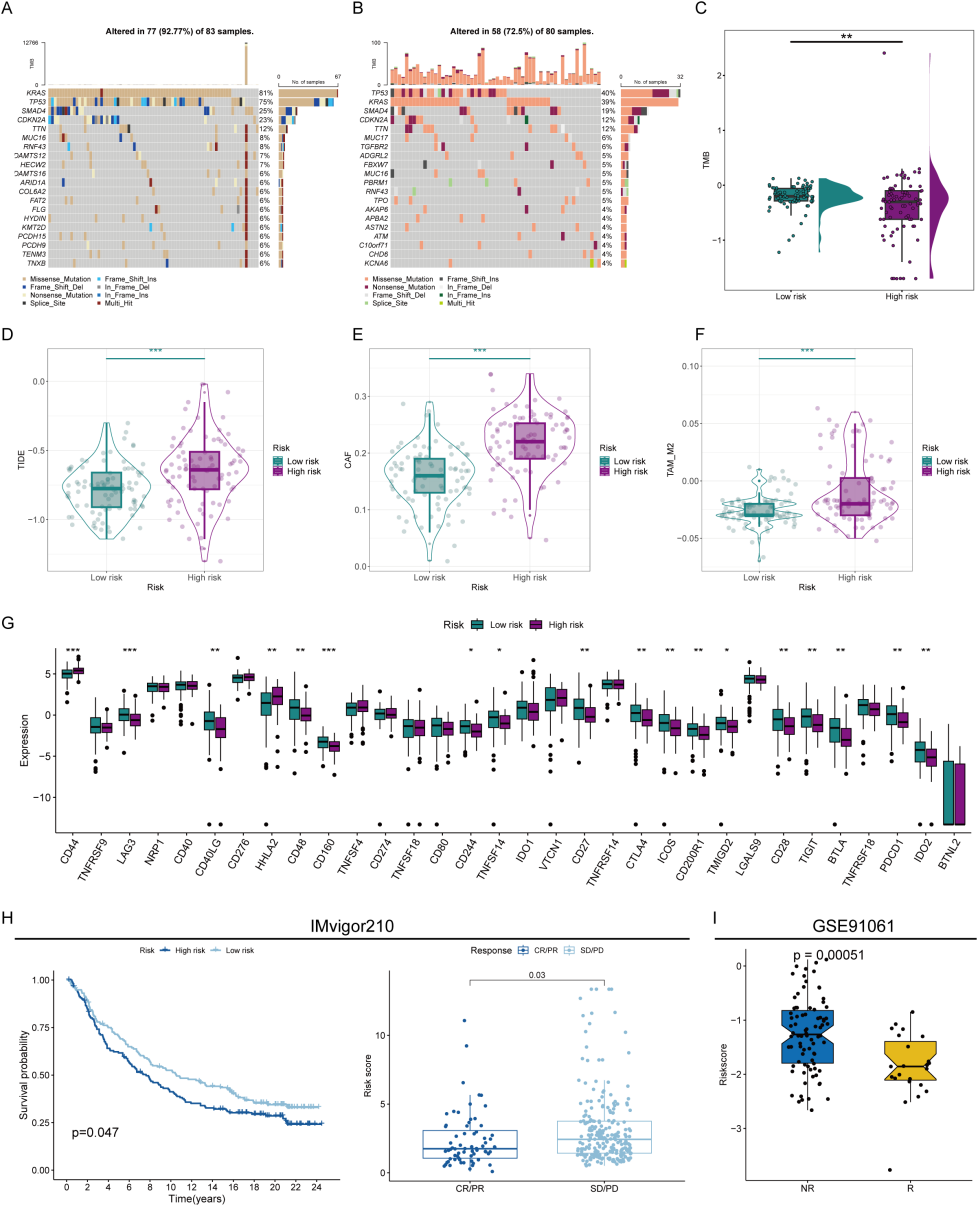
Prediction of immunotherapy sensitivity. **(A)** Mutation analysis of patients in the high- and low-risk groups. **(B)** Correlation analysis of different immunoreactive factors with the risk score. **(C)** Comparison of tumor mutation burden (TMB) in patients in the high- and low-risk groups. **(D)** TIDE scores of high- and low-risk score patients. **(E, F)** Comparison of CAF **(E)** and TAM_M2 **(F)** infiltration levels in the immune microenvironment of patients in the high- and low-risk groups. **(G)** Assessment of the expression abundance of immune checkpoint molecules. **(H, I)** Prediction of immunotherapy efficacy by risk score in immunotherapy cohorts. * represents p<0.05; ** represents p<0.01; *** represents p<0.001.

To further demonstrate the immunotherapy sensitivity of patients by degree of risk, we first assessed their mutation status. The proportion of mutations was higher in patients in the low-risk group (92.77%), especially in KRAS, with a mutation rate reaching 81%. Furthermore, the tumor mutational load is higher in low-risk individuals, representing that immunotherapy may be more likely to be beneficial for these patients (**Figures 6A–C**). Besides, the high-risk group’s higher TIDE scores suggested a lesser chance of benefit since they showed signs of rejection and immunological dysregulation. (**Figures 6D–F**). The low-risk group also showed higher expression of conventional immune checkpoints like CTLA-4 and PD1, indicating that these patients are better suited to start immunotherapy. (**Figure 6G**). To prove that the prognostic model could help assess the possibility of immunotherapy, we used two external treatment cohorts for validation. In the IMvigor210 cohort, high-risk patients assessed by our prognostic model also had significantly lower overall survival probabilities, and the risk scores of patients with complete remission and partial remission were also significantly lower than those of patients with stable disease and progressive disease (**Figure 6H**). Another immunotherapy dataset also revealed that immunoreactive patients had lower risk scores (**Figure 6I**), illustrating our model’s strong immunological response prediction capabilities.

### Crosstalk between PHLDA1^+^ CAFs and malignant ductal cells

**Figure 7:**
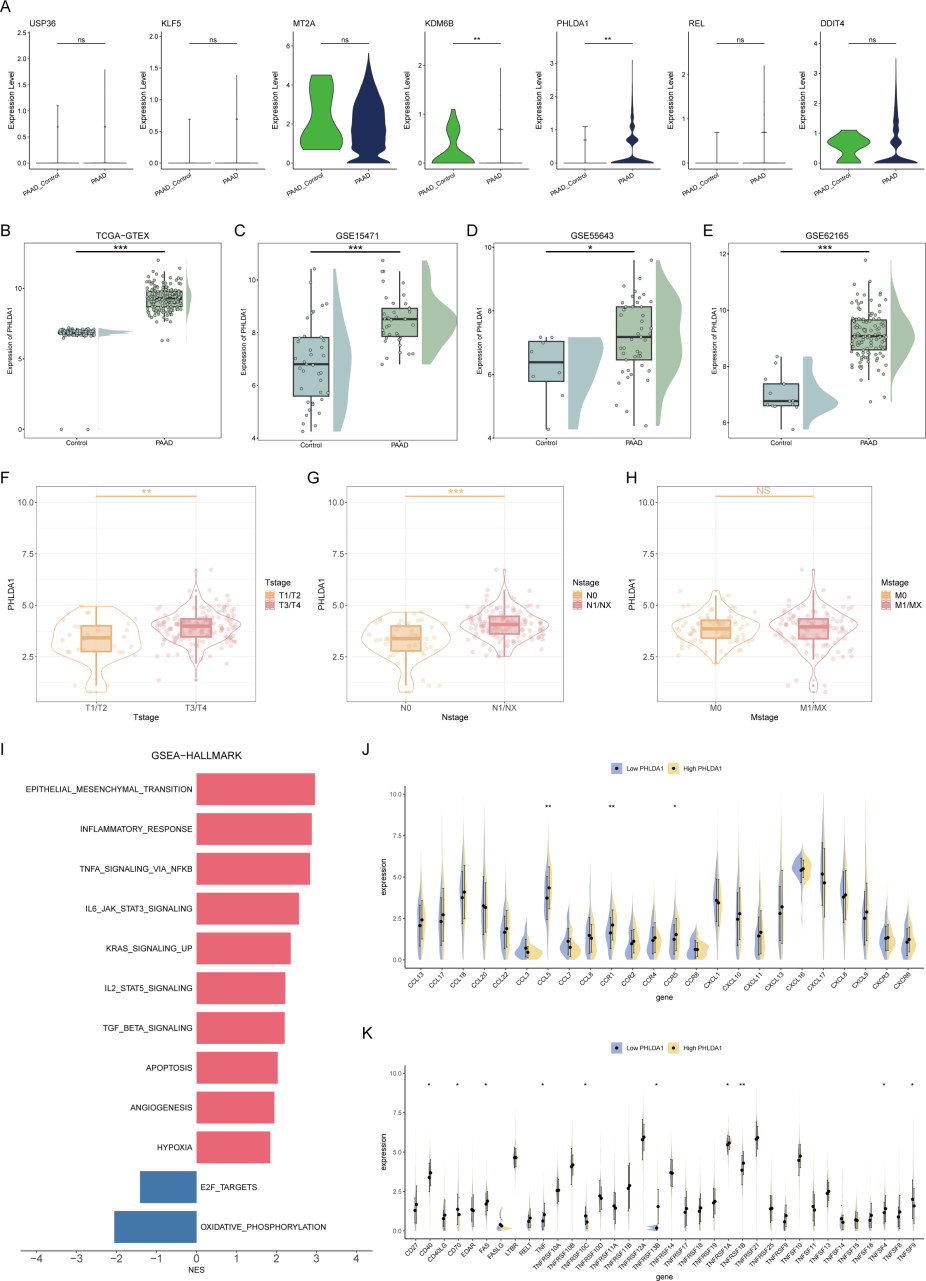
Screening of key molecular markers and intervention targets. **(A)** Box plots representing the expression levels of 7 model genes in pancreatic cancer and normal tissues. **(B–E)** Expression of PHLDA1 in different datasets. **(F–H)** The expression levels of PHLDA1 at different T stages **(F)**, N stages **(G),** and M stages **(H)** were compared. **(I)** GSEA revealed enriched pathways in patients with high or low PHLDA1 expression in pancreatic cancer. **(J, K)** Differences in the expression levels of chemokines **(J)** and TNF family molecules **(K)** in patients with high or low PHLDA1 expression in pancreatic cancer. * represents p<0.05; ** represents p<0.01; *** represents p<0.001.

**Figure 8:**
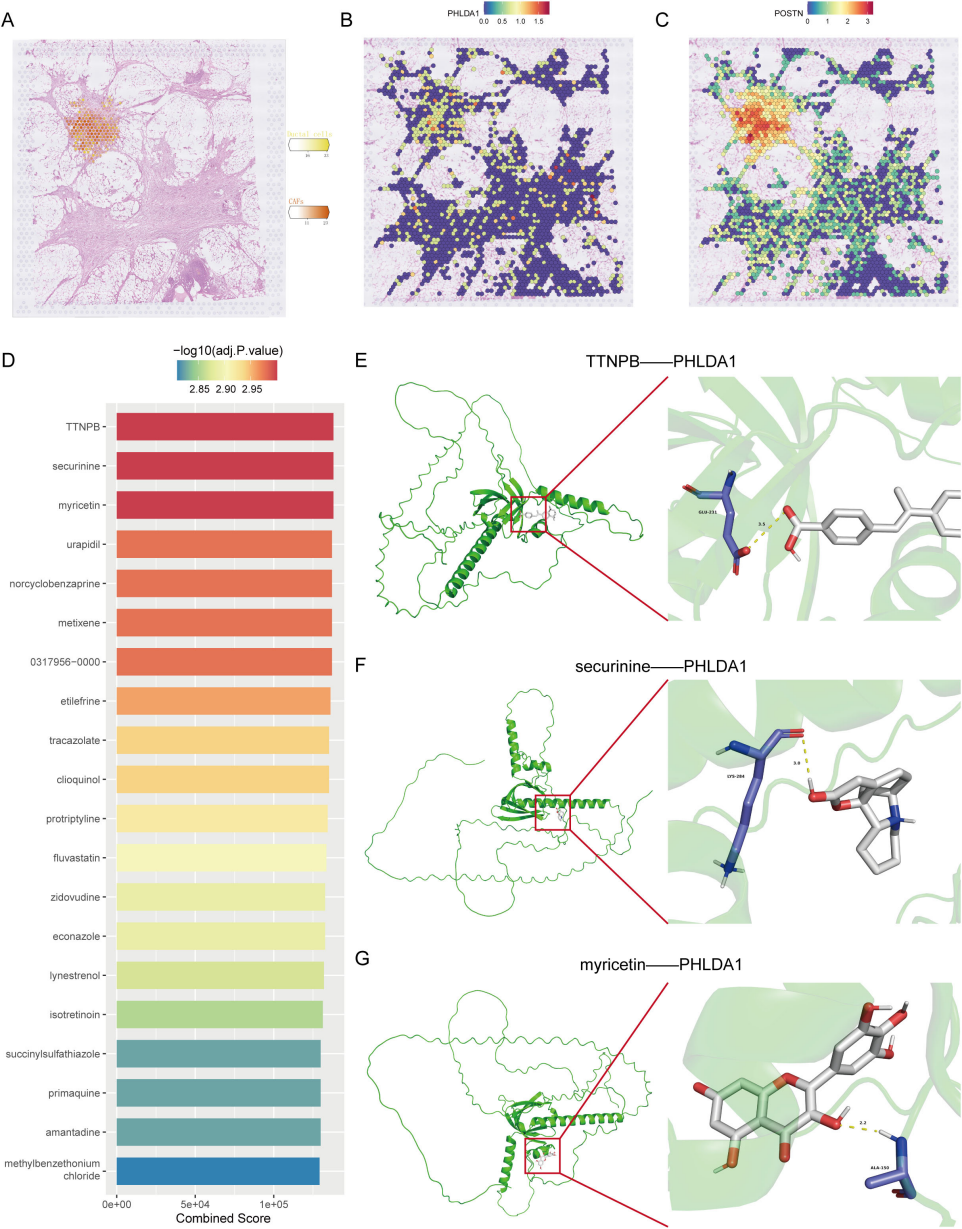
Spatial transcriptomic analysis and target molecule docking of PHLDA1. **(A)** Spatial distribution of CAFs and malignant ductal cells in pancreatic cancer. **(B, C)** Distribution of PHLDA1 **(B)** and POSTN **(C)** expression in the region of colocalization of CAFs with malignant duct cells. **(D)** Top 20 drugs targeting PHLDA1 in the DSigDB database. **(E, G)** Three-dimensional structure of the molecular docking of PHLDA1 with TTNPB **(E)**, securinine **(F)** and myricetin **(G)**.

Subsequently, we further confirmed the most important genes could be used as molecular markers and intervention targets. Through the examination of seven key genes’ expression levels, only PHLDA1 was highly expressed in CAFs in the pancreatic cancer group, suggesting that this gene could be a potential procancer CAF marker (**Figure 7A**). In four pancreatic cancer datasets, including the TCGA-GTEX cohort, PHLDA1 expression was remarkably elevated in the pancreatic cancer samples (**Figures 7B–E**). Meanwhile, PHLDA1 was related to TNM stage, and poorer TNM stages are associated with higher PHLDA1 expression (**Figures 7F–H**). Further enrichment analysis demonstrated that high PHLDA1 expression activated classic protumor pathways such as the EMT, KRAS, and TGFB pathways (**Figure 7I**). Moreover, patients with high PHLDA1 expression also presented increased expression of chemokines and TNF family members, including CCL5, CCR1, and TNFRSF1B (**Figures 7J, K**). Spatial transcriptome analysis can better observe the spatial location of cells based on more spatial information. Unrolling cell types into tissue sections revealed significant colocalization of CAFs and ductal cells, indicating a clear spatial interaction between the two (**Figure 8A**). Moreover, the expression of PHLDA1 and the CAF marker POSTN was also concentrated in the colocalization area of CAFs and ductal cells, indicating that PHLDA1 may mediate their interaction (**Figures 8B, C**).

### Screening of small molecule drugs and molecular docking

Drug screening is an essential step for the clinical translation of molecular targets. To screen for potential drugs targeting PHLDA1, we carried out a drug screening based on DSigDB and identified the top 20 drugs according to their binding scores. Among them, three drugs, TTNPB, securinine, and myricetin, had the highest binding scores (**Figure 8D**). To further determine which drug has the best binding rate with PHLDA1, molecular docking was performed separately for the three drugs with PHLDA1. As seen in **Figures 8E–G**, TTNPB presented the lowest binding energy of -6.816 kcal/mol, indicating that it bound most stably with PHLDA1 and could potentially be a PHLDA1-targeted drug.

In our drug‐screening pipeline, TTNPB emerged as the top candidate for PHLDA1 targeting based on its lowest docking energy among the top 20 ranked compounds. TTNPB is a well‐characterized synthetic retinoic acid receptor (RAR) agonist that has previously been shown to modulate fibroblast differentiation and extracellular matrix remodeling in various contexts. Although no studies to date have directly linked TTNPB to PHLDA1 inhibition, several reports indicate that RAR activation can suppress profibrotic signaling cascades (e.g., TGF-β/Smad) in stromal fibroblasts, which raises the possibility that TTNPB may indirectly attenuate PHLDA1‐driven CAF activation. Moreover, retinoid signaling has been reported to downregulate key EMT‐associated transcription factors—many of which overlap with PHLDA1 downstream effectors—thereby providing a mechanistic rationale for TTNPB’s potential efficacy in disrupting CAF–tumor crosstalk. Future work should therefore prioritize *in vivo* validation of TTNPB in CAF‐rich pancreatic cancer models, such as co‐implantation of PHLDA1‐high CAFs with orthotopic tumor cells, to assess whether pharmacologic RAR activation can reduce tumor stiffness, limit desmoplasia, and enhance anti‐tumor immunity. Additionally, given the established immunosuppressive role of CAFs, combining TTNPB with immune‐checkpoint inhibitors (e.g., anti‐PD-1/PD-L1) or other stroma-modulating agents may further potentiate therapeutic responses. Such combinatorial strategies could help overcome the stromal barriers that frequently limit drug delivery and immunotherapy efficacy in pancreatic cancer.

### PHLDA1^+^ CAFs promote malignant progression in pancreatic cancer

**Figure 9:**
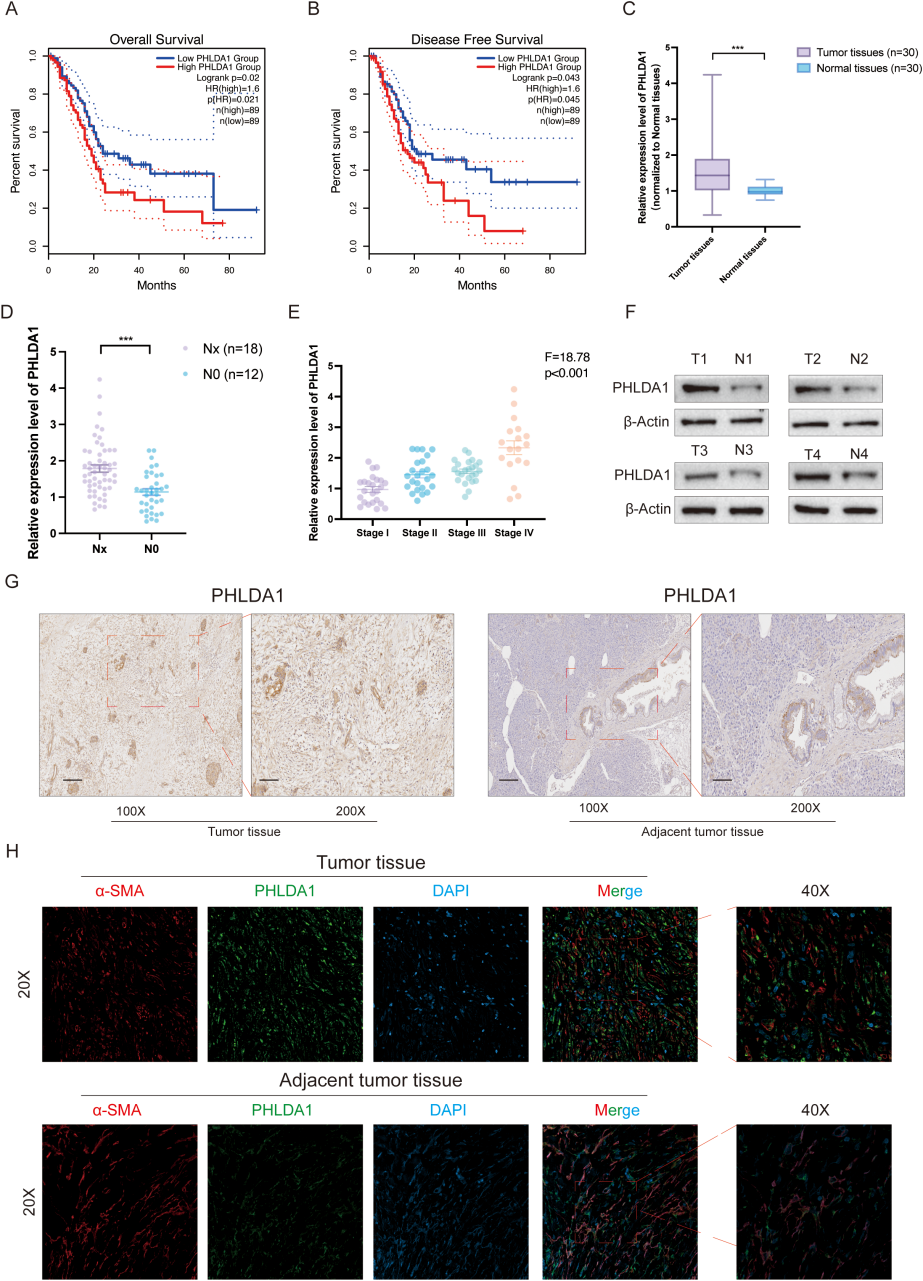
PHLDA1 is highly expressed and associated with poor prognosis in pancreatic cancer patients. **(A, B)** The overall survival **(A)** and disease-free survival **(B)** analysis of PHLDA1 in pancreatic cancer patients. **(C)** The mRNA expression level of PHLDA1 in tumor tissues (n=30) and paired adjacent tumor tissues (n=30). **(D)** The mRNA expression level of PHLDA1 in patients with lymph node metastasis. **(E)** PHLDA1 expression in patients with different pathological stages (n=30). **(F)** Western blot showing the protein level of PHLDA1 in tumor tissues and paired normal tissues (n=4). **(G)** IHC staining showing the expression level of PHLDA1 in tumor tissues and paracancerous tissues. **(H)** Colocalized distribution of PHLDA1 with the CAF marker α-SMA in cancer and adjacent tumor tissues.

**Figure 10:**
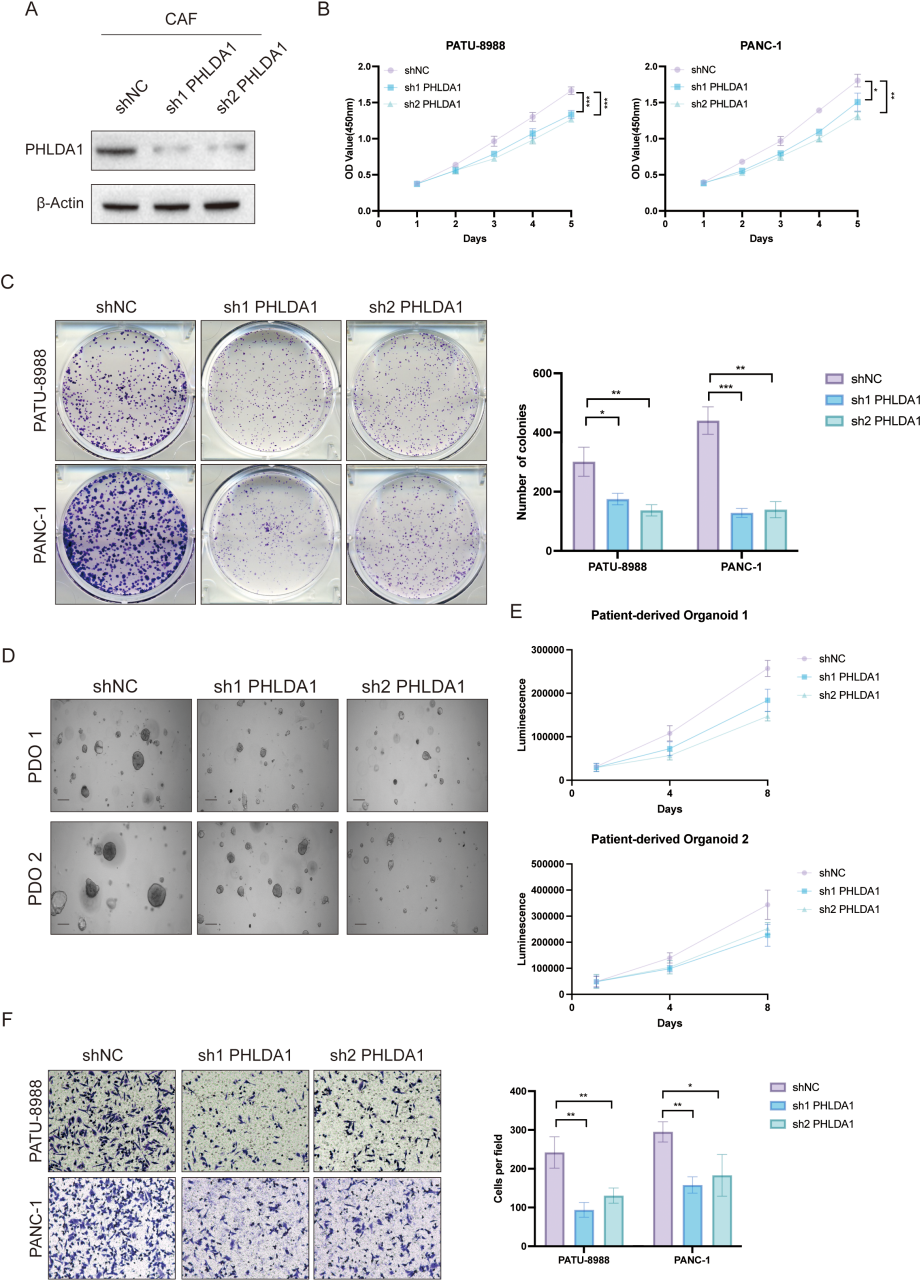
PHLDA1^+^ CAF facilitated malignant biological behavior in pancreatic cancer. **(A)** Knockdown efficiency of PHLDA1 at the protein level in CAFs. **(B)** When PHLDA1 was inhibited, CCK8 assays were used to detect the proliferative activity of PANC-1 or PATU-8988 cells when cocultured with CAFs. **(C)** Colony formation assays in PATU-8988 and PANC1 cells after cocultured with CAFs (shNC, sh1 PHLDA1, sh2 PHLDA1). **(D)** Representative images of PDO 1 or PDO 2 co-cultured with CAFs. **(E)** CTG assays revealed the proliferative capacity of different patient-derived organoids after cocultured with CAFs in Days 1, 4, and 8. **(F)** Evaluation of the migration capacity of PATU-8988 and PANC-1 cells after cocultured with CAFs (shNC, sh1 PHLDA1, sh2 PHLDA1). * represents p<0.05; ** represents p<0.01; *** represents p<0.001.

Next, we investigated the role of PHLDA1 in the development of pancreatic cancer. According to survival analysis, patients with high PHLDA1 expression had a worse prognosis than patients with low PHLDA1 expression, which suggested that PHLDA1 could be a possible prognostic marker for pancreatic cancer (**Figures 9A, B**). Also, the expression of PHLDA1 in clinical samples of patients with pancreatic cancer was subsequently described. It could be observed higher mRNA expression of PHLDA1 in tumor tissues (**Figure 9C**). At the same time, it was linked to lymph node metastases and worse pathological staging characteristics according to paired tumor tissues and adjacent tissues (**Figures 9D, E**) (**Supplementary Table S1**). Moreover, PHLDA1 was shown to be expressed differently in tumor and adjuvant tumor tissues by further western blot and IHC staining (**Figures 9F, G**). Following the previous analysis, immunofluorescence was performed on the tumor sample and adjuvant tumor sample, and the findings indicated that PHLDA1 was expressed primarily in CAFs in pancreatic cancer (**Figure 9H**). As demonstrated in **Figures 10A–C**, downregulating PHLDA1 in CAFs dramatically decreased tumor cell proliferation activity, and similar results were achieved in colony formation assays. Additionally, patient-derived organoids were cocultured with CAFs, and we discovered that when PHLDA1 expression was reduced in CAFs, organoid proliferation ability was limited (**Figures 10D, E**). To ascertain if PHLDA1 in CAFs aided in the migration of pancreatic cancer cells, transwell experiments were yd. As expected, PHLDA1 considerably increased the migration capacity of PATU-8988 and PANC-1 cells (**Figure 10F**). Together, our findings suggest that PHLDA1 serves as a prognostic biomarker in pancreatic cancer and influences tumor growth by modulating cancer-cell proliferation and migration.

### PHLDA1 reflects prognosis and immune status in multiple tumors

**Figure 11:**
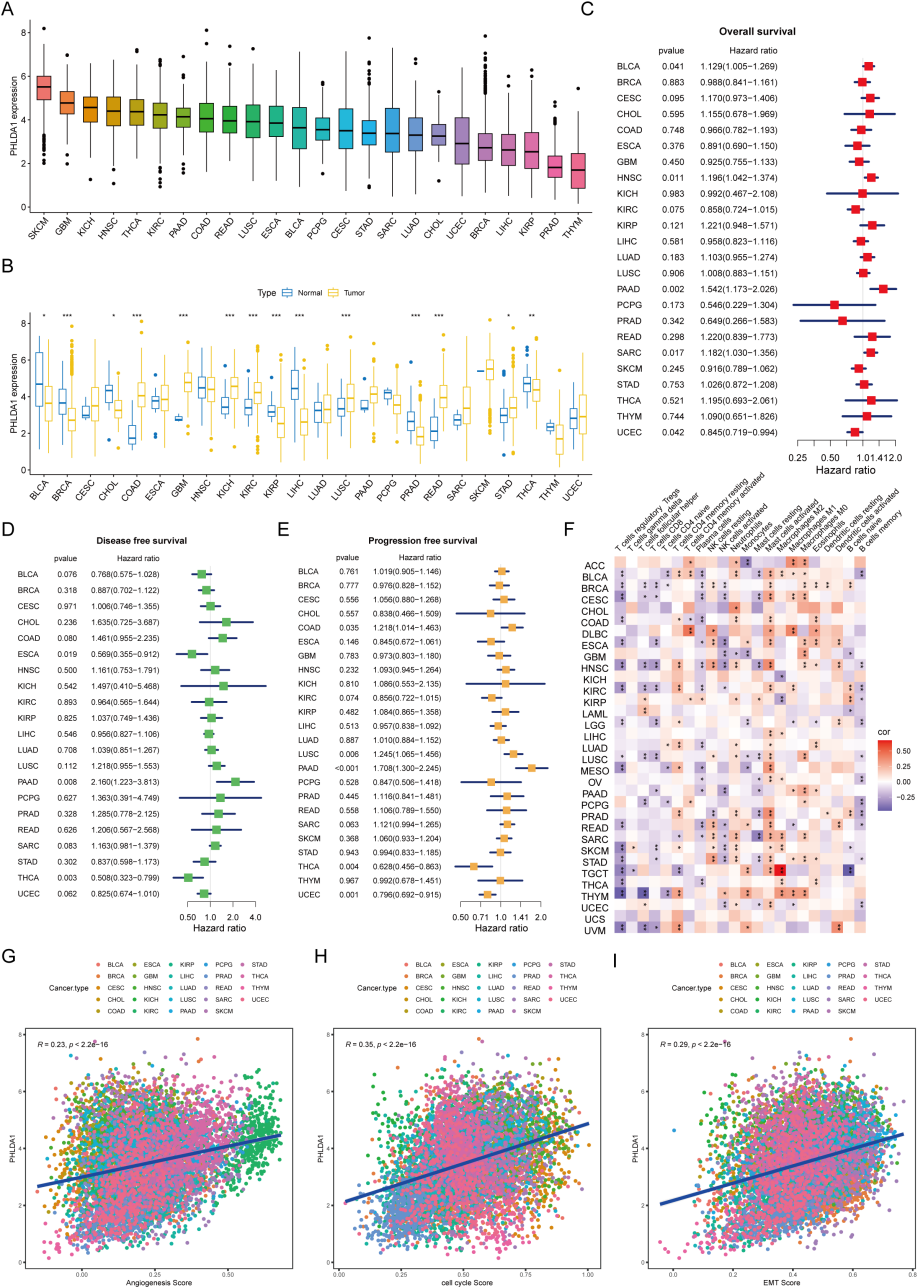
The applicability of PHLDA1 at the pan-cancer level. **(A)** The mRNA expression level of PHLDA1 in various cancer types. **(B)** PHLDA1 expression in the tumor and adjacent tumor tissues of different tumors. **(C–E)** The overall survival **(C)**, disease-free survival **(D)** and progression-free survival **(E)** of PHLDA1 at the pan-cancer level. **(F)** Correlations of PHLDA1 with the infiltration of various immune cells in multiple tumor types. **(G, H)** Evaluation of PHLDA1 expression, angiogenesis score, cell cycle score and EMT score in a range of cancers. * represents p<0.05; ** represents p<0.01; *** represents p<0.001.

Finally, to broaden the application of PHLDA1, we analyzed its applicability across various types of cancer. It could be seen that melanomas exhibited the highest PHLDA1 expression, while thymomas showed the lowest (**Figure 11A**). The expression of this gene varies among different tumors. Specifically, PHLDA1 was highly expressed in the control group for bladder cancer, breast cancer, cholangiocarcinoma, renal papillary cell carcinoma, liver cancer, prostate cancer, and thyroid cancer, whereas it was highly expressed in the tumor groups for colorectal cancer, glioma, renal clear cell carcinoma, lung squamous cell carcinoma, rectal cancer, and gastric cancer (**Figure 11B**). PHLDA1 was found to be substantially associated with disease-free survival in patients with thyroid cancer, head and neck squamous cell carcinoma, pancreatic cancer, soft tissue sarcoma, bladder cancer, and endometrial cancer, as well as with overall survival in these patients. It was also significantly related to progression-free survival in patients with colorectal cancer, lung adenocarcinoma, pancreatic cancer, lung squamous cell carcinoma, thyroid cancer, and endometrial cancer (**Figures 11C–E**), suggesting that this indicator could be used to guide survival prognosis in these types of cancer. Additionally, PHLDA1 and the quantity of activated mast cells in practically all cancer types showed a strong positive connection, according to immune infiltration study, indicating that mast cells may contribute to the development of cancer (**Figure 11F**). Finally, the correlation analysis uncovered that angiogenesis, cell cycle, and EMT were significantly positively correlated with PHLDA1 in all types of cancer, further suggesting the cancer-promoting role of PHLDA1 (**Figures 11G–I**).

## Discussion

An increasing body of research has highlighted the pronounced intratumoral heterogeneity within pancreatic cancer (PC), posing significant challenges for the development of effective therapeutic strategies. Therefore, finding innovative treatment strategies is crucial to raising PC patients’ overall survival rates. A growing body of research suggests that the complex tumor microenvironment’s (TME) neoplastic and stromal cells’ intercellular communication is closely related to the tumor cells’ malignant biological activities (36–38). As essential components of the TME, cancer-associated fibroblasts (CAFs) are known to influence important facets of carcinogenesis, such as metastasis, angiogenesis, proliferation, and resistance to different treatment modalities in a variety of cancers (12, 39, 40). Additionally, mounting data emphasizes how crucial CAFs are in initiating drug susceptibility in pancreatic cancer to immunotherapy, targeted treatment, chemotherapy, and radiation (41, 42). According to certain report, the formation and spread of cancer are directly linked to the interactions between various cell types inside the TME (43). Our analysis of published single-cell RNA sequencing (scRNA-seq) data revealed an increased proportion of ductal and CAF cells in PC tissues, along with enhanced interactions between these cell types as shown by CellChat analysis. In addition, a higher prevalence of CAFs was correlated with advanced disease stages and poorer overall survival. These observations indicate a potential involvement of CAFs in PC progression.

CAFs are a heterogeneous population arising from various cell types across solid tumors, activated by multiple signaling pathways (44). Their diverse origins and activation mechanisms generate a spectrum of phenotypes, resulting in functional heterogeneity. CAFs were first divided into two subgroups (CAF-A and CAF-B) via single-cell sequencing in colorectal cancer (45). In recent years, CAFs have been divided into three groups based on their roles in lung, prostate, and triple-negative breast cancer: myofibroblastic CAFs (myCAFs), inflammatory CAFs (iCAFs), and matrix CAFs (mCAFs) (46–48). Our goal in this study was to clarify how CAFs affect PC’s biological behavior. Comprehensive analysis revealed the existence of five distinct CAF subpopulations, namely, iCAFs, progenitor cell CAFs (proCAFs), mCAFs, MT2A-expressing myofibroblastic CAFs (MT2A+ myCAFs), and CXCL14-expressing myofibroblastic CAFs (CXCL14+ myCAFs). Among these, mCAFs have emerged as the most critical malignant CAF subgroup with the potential to predict the prognosis of PC patients. We developed a 7-gene mCAF-related gene risk model, which allowed us to accurately forecast the survival rates of patients by classifying them into high-risk and low-risk groups. This stratification approach holds promise for improving prognostic accuracy and may inform personalized treatment strategies for PC patients.

CAFs are intricately involved in the progression of PC and are closely linked to metastasis, immune evasion, and resistance to immunotherapy (49). Within the TME, CAFs engage tumor cells and other stromal components, producing excess extracellular matrix proteins, soluble mediators, and matrix-degrading enzymes. These activities result in increased matrix deposition, increased interstitial pressure, and compression of blood vessels, which collectively contribute to hypoxia and nutrient deprivation (50, 51). Consequently, this environment restricts the administration of chemotherapeutic drugs and prevents immune cells from infiltrating.

In this study, we employed our established risk model to assess the immune profiles of patients, as well as their potential response to immunotherapy. Our findings showed that high-risk patients exhibited a pronounced inflammatory cytokine surge, which correlates with chronic tumor progression (52). Moreover, the high-risk group displayed immune dysfunction, weakened antitumor responses, and an intensified protumorigenic microenvironment. Our risk model demonstrated statistically significant predictive accuracy for immunotherapy outcomes in PC patients, suggesting its potential utility in guiding patient care and clinical decision-making.

Prior investigation has emphasized how crucial the temporal and spatial dynamics within the TME are in promoting tumor heterogeneity (53). Malignant tumor invasiveness, metastatic potential, and clinical outcomes have been found to be substantially associated with the density and spatial distribution of immune cells across different tissue subregions (54). These conclusions are supported by published data, which also confirm that the spatial variability of CAFs significantly affects cancer patients’ survival (55). These observations highlight the importance of considering the spatial architecture of the TME when examining the biology of cancer and its response to therapy. Although previous studies have characterized CAF signatures based on a limited set of markers (56), our study extends these findings by identifying a distinct PHLDA1-positive CAF subtype that is significantly associated with poor prognosis and tumor-stroma interactions. Recent advancements in single-cell and spatial transcriptomic technologies have revealed that specific CAF subpopulations play critical roles in mediating immunotherapy resistance and overall patient outcomes (8, 17). Importantly, our data suggest that targeting PHLDA1-positive CAFs could serve as a novel therapeutic strategy, thereby bridging the gap between conventional CAF classifications and personalized treatment approaches (16). In this context, our findings not only refine the existing CAF paradigm but also enhance its clinical applicability by providing a more robust biomarker for prognostic stratification and therapeutic decision-making.

Consistent with these findings, our analysis revealed that mCAFs are in close proximity to malignant ductal cells and that their spatial distribution and density within tumor tissues are correlated with the aggressiveness and prognosis of PC. According to these findings, mCAFs most likely promote PC development by having a direct impact on the biological activity of ductal cells. Notably, mCAFs’ spatial heterogeneity may play a role in the regulation of tumor behavior. Specifically, mCAFs located at different spatial distances from ductal cells may carry out unique biological tasks that could influence tumor growth and invasion through a variety of mechanisms of action, ultimately accelerating the advancement of the tumor. Further mechanistic analysis revealed that elevated expression of PHLDA1 promotes CAF activation and proliferation by activating key signaling pathways such as TGF-β and KRAS, thereby inducing epithelial-mesenchymal transition (EMT) and enhancing extracellular matrix (ECM) deposition and remodeling. This ECM remodeling increases tissue stiffness and density, creating physical barriers that restrict immune infiltration and therapeutic drug penetration, consequently facilitating invasion and migration of pancreatic cancer cells and leading to poor patient prognosis. These findings uncover the underlying mechanisms by which PHLDA1+ CAFs shape the tumor microenvironment and drive pancreatic cancer progression.

To provide more direct evidence for PHLDA1’s functional involvement in key protumorigenic pathways, we examined both published mechanistic studies and our own co-culture results. PHLDA1 has been identified as a direct TGF-β/SMAD target, with TGF-β1 treatment increasing PHLDA1 mRNA expression approximately threefold in keratinocyte models, and chromatin immunoprecipitation confirmed SMAD3/SMAD4 binding to a regulatory region upstream of PHLDA1 (57). In addition, PHLDA1 overexpression can induce β-catenin nuclear localization, disrupt adherens junctions, and trigger EMT-associated transcriptional programs—specifically upregulating SNAI1 and VIM—consistent with a functional role in EMT induction (58). And, PHLDA1 augments KRAS pathway activity by stabilizing the RAS–ERK axis: in glioblastoma, PHLDA1 binds to Ras and competitively inhibits Src-mediated Ras phosphorylation, resulting in sustained Ras-GTP levels, increased RAF/MEK/ERK signaling, and elevated downstream MYC transcription; conversely, PHLDA1 knockdown reduces phospho-ERK1/2 levels by approximately 50 % (59).

PHLDA1 (also known as TDAG51) is a pleckstrin homology domain-containing protein originally implicated in regulating apoptosis, cell proliferation, and cellular stress responses. Under physiological conditions, PHLDA1 plays a role in maintaining cellular homeostasis and signaling balance, as well as in specific differentiation processes. Notably, prior studies have reported that PHLDA1 is involved in several key signaling pathways, including the PI3K/Akt, TGF-β, and KRAS pathways. In our study, elevated PHLDA1 expression was associated with the activation of protumorigenic pathways such as EMT, KRAS, and TGF-β, suggesting that it may contribute to the regulation of tumor cell transformation, invasion, and migration. Moreover, the high expression of PHLDA1 in CAFs and its significant correlation with poor prognosis indicate that it plays an important role in modulating the tumor microenvironment. Therefore, targeting PHLDA1 might not only disrupt the detrimental crosstalk between CAFs and tumor cells but also enhance the efficacy of conventional therapies, offering a promising new avenue for pancreatic cancer treatment. Further studies are needed to fully elucidate its mechanisms and validate its potential as a therapeutic target.

Although we applied established batch-correction tools (e.g., Harmony for single-cell data and sva for bulk RNA-seq), some technical variability from library preparation, sequencing platforms, and sample handling likely persists. These residual batch effects may obscure subtle transcriptomic differences, especially among rare cell populations. Moreover, although pan-cancer analysis highlights the broad prognostic and immunomodulatory relevance of PHLDA1, interpretation of these cross-tumor associations must be tempered by the fact that expression patterns and downstream signaling networks can vary dramatically between tumor lineages. In particular, tissue-specific microenvironmental cues and distinct oncogenic drivers in each cancer type may confound the generalizability of PHLDA1’s functional role. Future work incorporating uniformly processed samples and validation in lineage-matched models will be essential to disentangle true biological signals from cohort-specific artifacts.

Although our study focused on the enrichment of PHLDA1 in mCAFs and its association with ECM remodeling and tumor invasion, it is important to place these findings within the broader context of CAF heterogeneity. In contrast to inflammatory CAFs (iCAFs), which are marked by high cytokine secretion, and myofibroblastic CAFs (myCAFs), known for their contractile and matrix-remodeling functions, our data indicate that PHLDA1+ mCAFs preferentially activate TGF-β and KRAS signaling pathways. This suggests that targeting PHLDA1+ mCAFs, either alone or in combination with interventions aimed at other CAF subtypes, could provide a more comprehensive strategy for disrupting tumor–stroma interactions and improving therapeutic outcomes in pancreatic cancer. Unlike traditional CAF markers such as FAP and α-SMA—which mainly indicate matrix remodeling and myofibroblastic activation, respectively—our results show that PHLDA1 specifically marks a CAF subset that is strongly associated with protumorigenic pathways (e.g., EMT, KRAS, and TGF-β signaling). In our study, high PHLDA1 expression correlated with advanced TNM stage and poorer overall survival, a relationship that was less pronounced for FAP and α-SMA. Furthermore, PHLDA1 knockdown in CAFs significantly impaired tumor cell proliferation and migration. These findings suggest that PHLDA1 not only offers superior prognostic value but also plays a direct role in mediating tumor-stroma crosstalk, thereby representing a promising therapeutic target.

In addition to molecular targeting of PHLDA1, recent bioengineering platforms offer promising avenues to overcome stromal barriers and reshape the tumor microenvironment. For example, Huang et al. developed a Christmas tree–shaped microneedle patch that achieved spatiotemporal delivery of FOLFIRINOX directly into orthotopic pancreatic tumors by layering oxaliplatin/leucovorin and irinotecan/fluorouracil within hierarchical microneedle tiers, significantly enhancing intratumoral penetration and drug retention. Such a device could be adapted to co-deliver PHLDA1 inhibitors alongside chemotherapeutics or CAF-modulating agents, thereby improving drug distribution in desmoplastic lesions. Similarly, Zetrini et al. engineered polymer–lipid manganese dioxide nanoparticles that consume hydrogen (60)peroxide to generate oxygen and buffer acidity, reoxygenating hypoxic tumors and driving macrophage polarization toward an M1 phenotype when combined with radiotherapy (61). Translating this platform to PHLDA1^high CAF-rich pancreatic tumors could normalize the microenvironment, attenuate CAF-mediated immunosuppression, and potentiate the efficacy of PHLDA1-targeted therapies and immune checkpoint blockade. Future studies should investigate the integration of microneedle-based spatiotemporal delivery and redox-active nanoparticle strategies in lineage-matched pancreatic cancer models to evaluate synergistic effects on stromal depletion and antitumor immunity.

In addition to molecular and delivery‐based innovations, emerging spatial genomics platforms and integrin–mTOR signaling studies offer novel avenues for future PHLDA1 research. For example, the recently described Perturb-DBiT technology enables simultaneous *in vivo* CRISPR screening and spatial transcriptomics, providing single‐cell resolution maps of how genetic perturbations affect both coding and noncoding RNAs within their native microenvironment (62). By applying Perturb-DBiT to CAF populations, researchers could uncover PHLDA1’s spatially resolved downstream effectors and identify context‐dependent interactions between CAFs and tumor cells *in situ*. Likewise, insights from integrin-mediated mTOR/TGF-β overactivity in fibrotic valve disease illuminate how integrin–mTOR axes drive profibrotic signaling and immune cell recruitment (63). Translating these findings to CAF biology suggests that integrin–mTOR inhibitors may synergize with PHLDA1‐targeted approaches to normalize the stroma and enhance anti‐tumor immunity. Altogether, integrating spatial CRISPR screens with targeted modulation of integrin–mTOR pathways could accelerate the development of more precise, microenvironment-focused therapies.

## Conclusion

In conclusion, our study found a novel CAF cluster with strong predictive significance and offered a thorough examination of ductal cells and fibroblasts in the PC tumor microenvironment. According to comprehensive RNA-seq and ST findings, the mCAF subset may facilitate PC development by directly interacting with ductal cells. Through additional pan-cancer analysis tests, we investigated the function of PHLDA1. To sum up, we detailed the variety of CAFs in PC and discovered a distinct mCAF isoform and target linked to tumor growth, which enables us to better comprehend why immunotherapy is so ineffective in this situation. The present research offers a workable concept for upcoming PC medication interventions.

## Data Availability

The original contributions presented in the study are included in the article/**Supplementary Material**. Further inquiries can be directed to the corresponding authors.
