# Supplementary figures and images for: Integrated multiomics analysis identifies PHLDA1+ fibroblasts as prognostic biomarkers and mediators of biological functions in pancreatic cancer
Source: Front Immunol. 2025 Jul 4;16:1592416. doi: 10.3389/fimmu.2025.1592416 (PMC12271128; doi:10.3389/fimmu.2025.1592416)

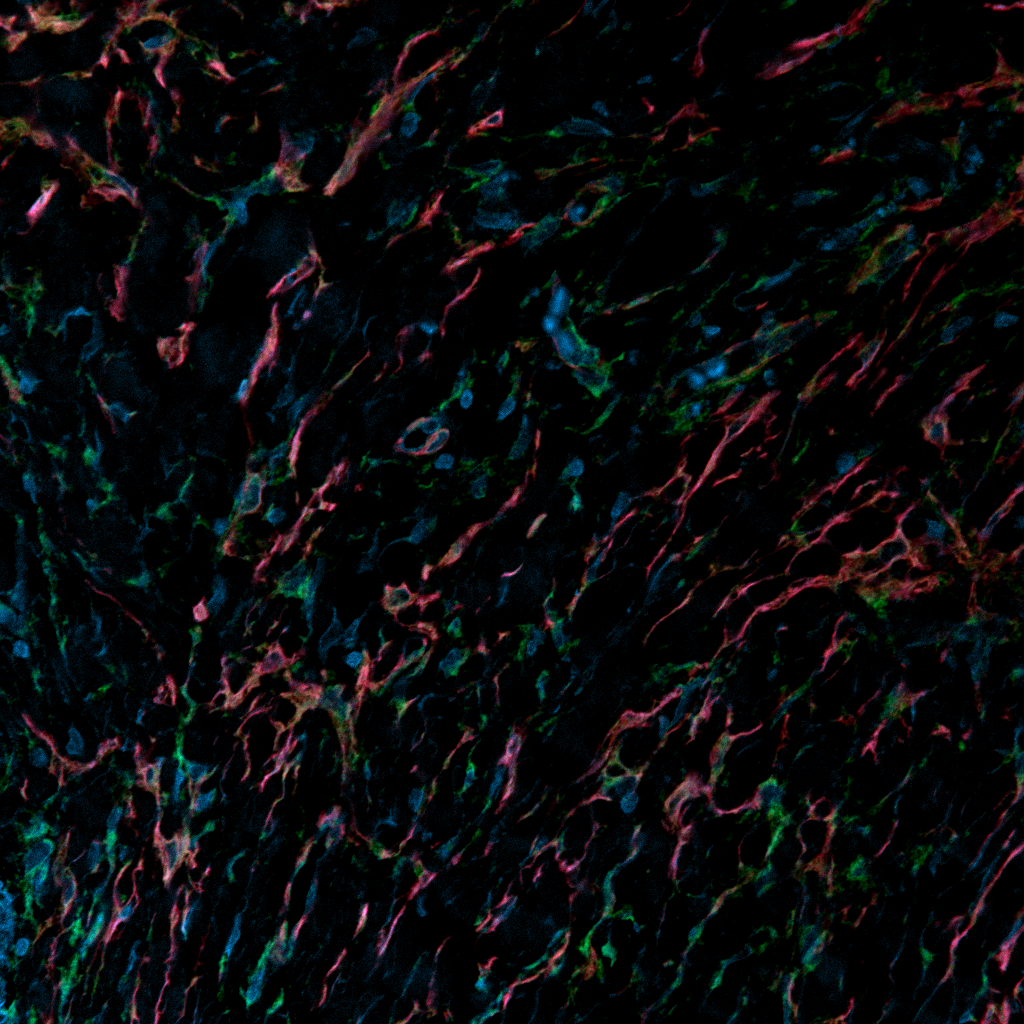

Supplement: Supplementary file 2 [file DataSheet2.zip › IF/p+a-normal-12.11 20x-1-Image Export-01_c1+2+3.tif]

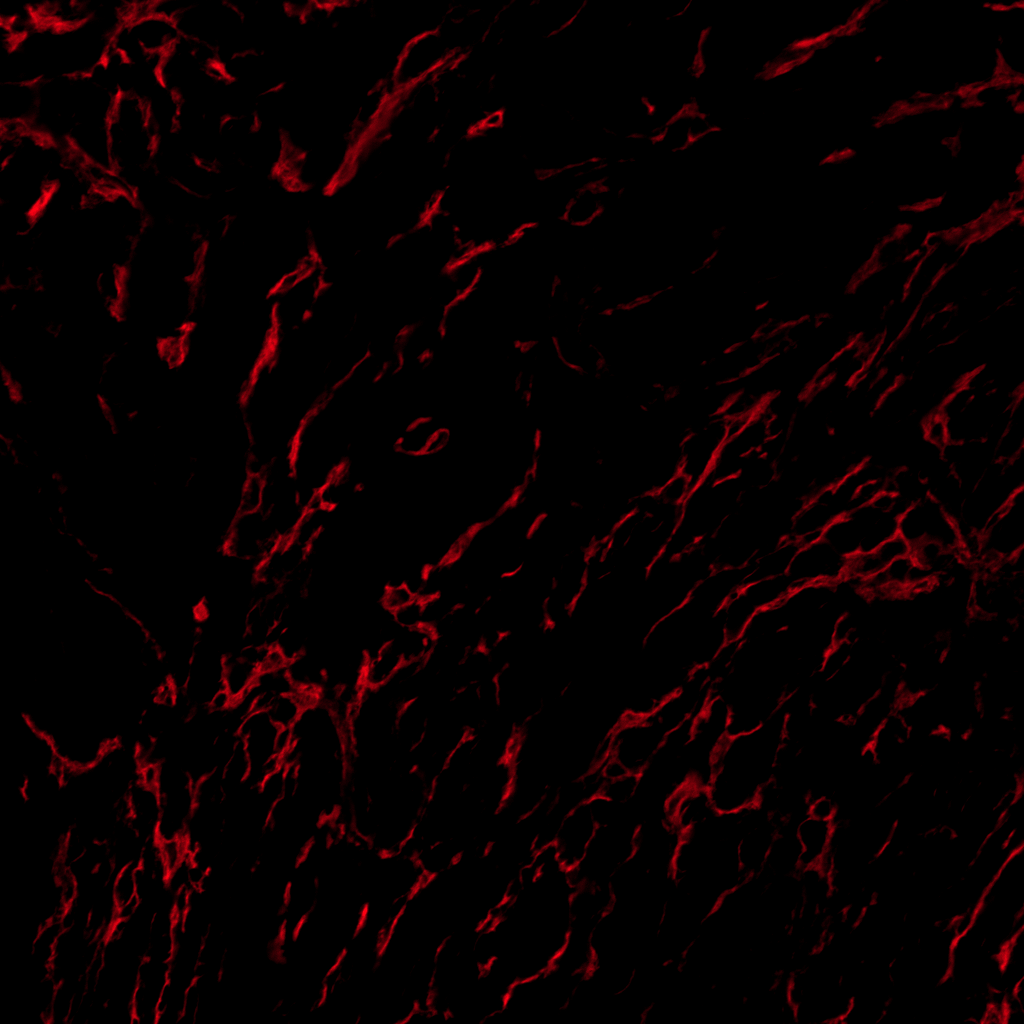

Supplement: Supplementary file 2 [file DataSheet2.zip › IF/p+a-normal-12.11 20x-1-Image Export-01_c1.tif]

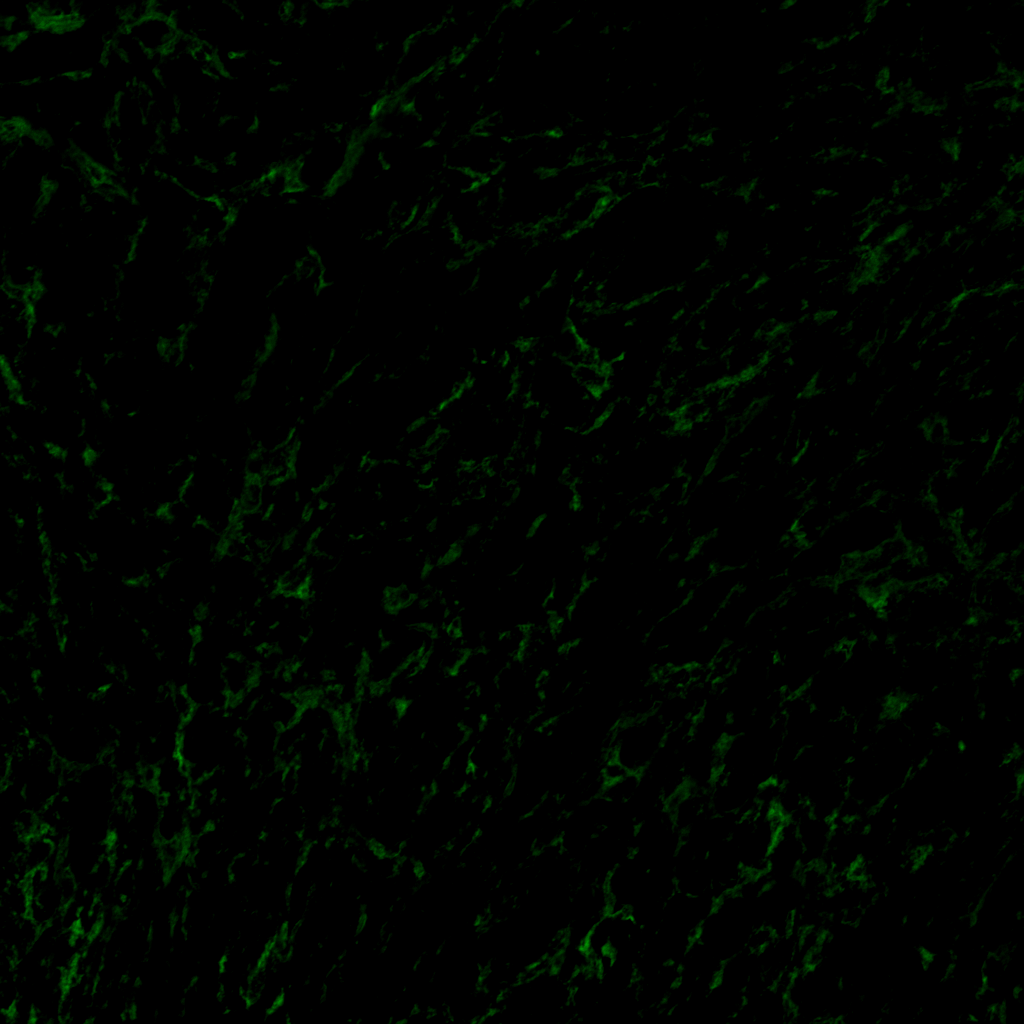

Supplement: Supplementary file 2 [file DataSheet2.zip › IF/p+a-normal-12.11 20x-1-Image Export-01_c2.tif]

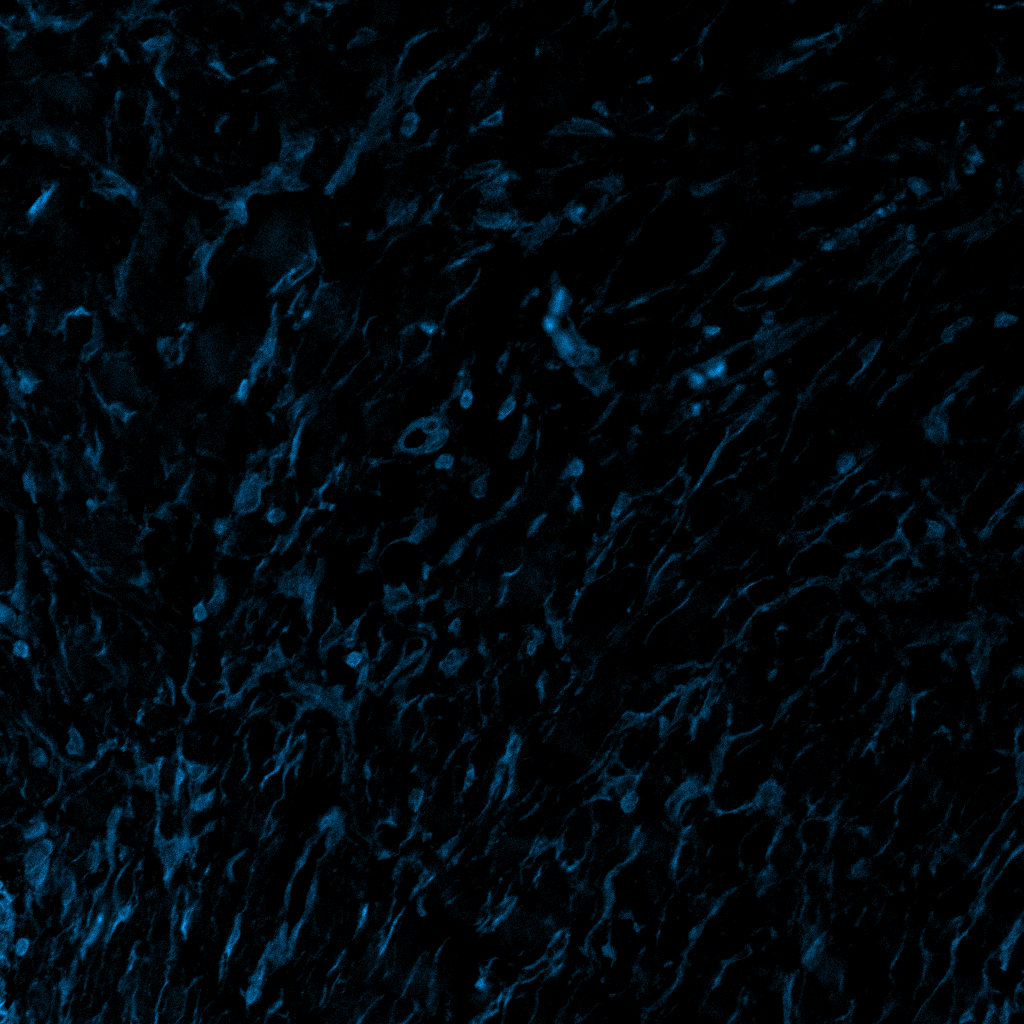

Supplement: Supplementary file 2 [file DataSheet2.zip › IF/p+a-normal-12.11 20x-1-Image Export-01_c3.tif]

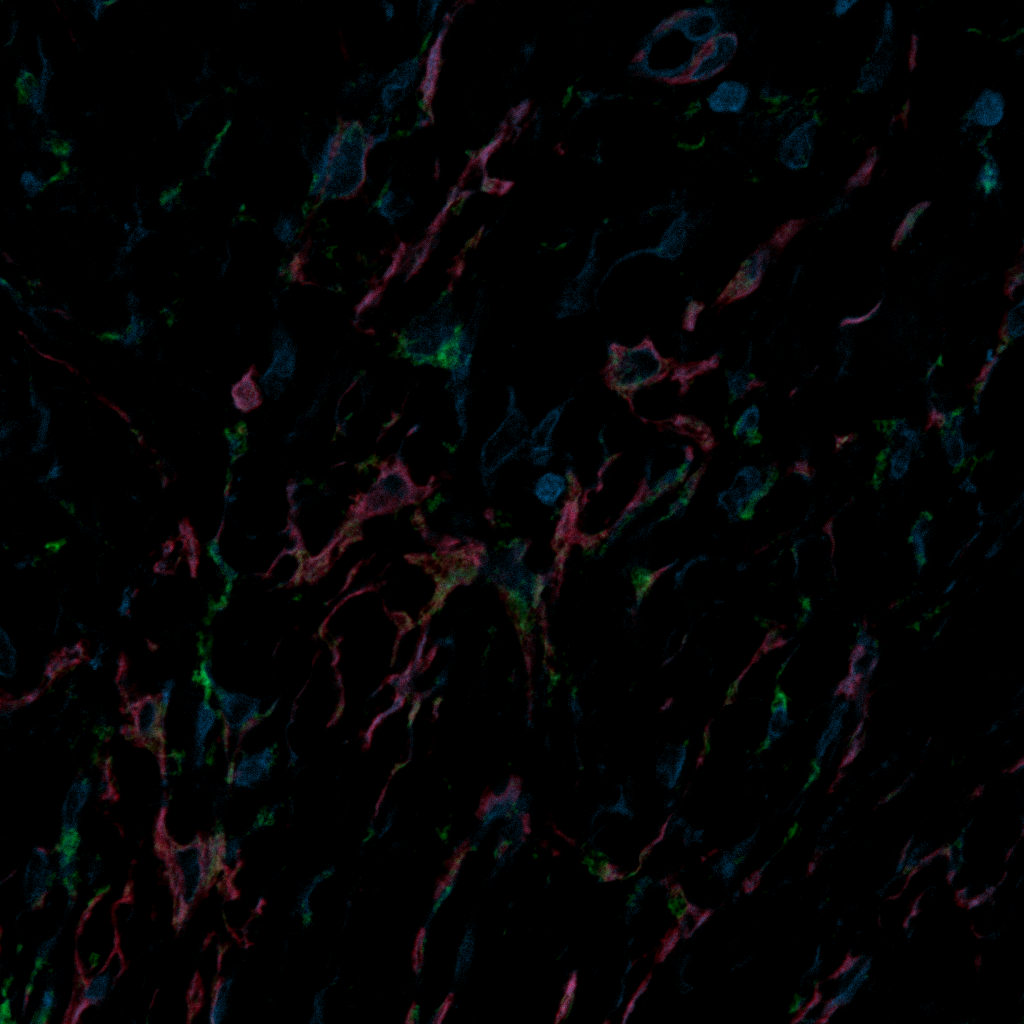

Supplement: Supplementary file 2 [file DataSheet2.zip › IF/p+a-normal-12.11 40x-1-Image Export-04_c1+2+3.tif]

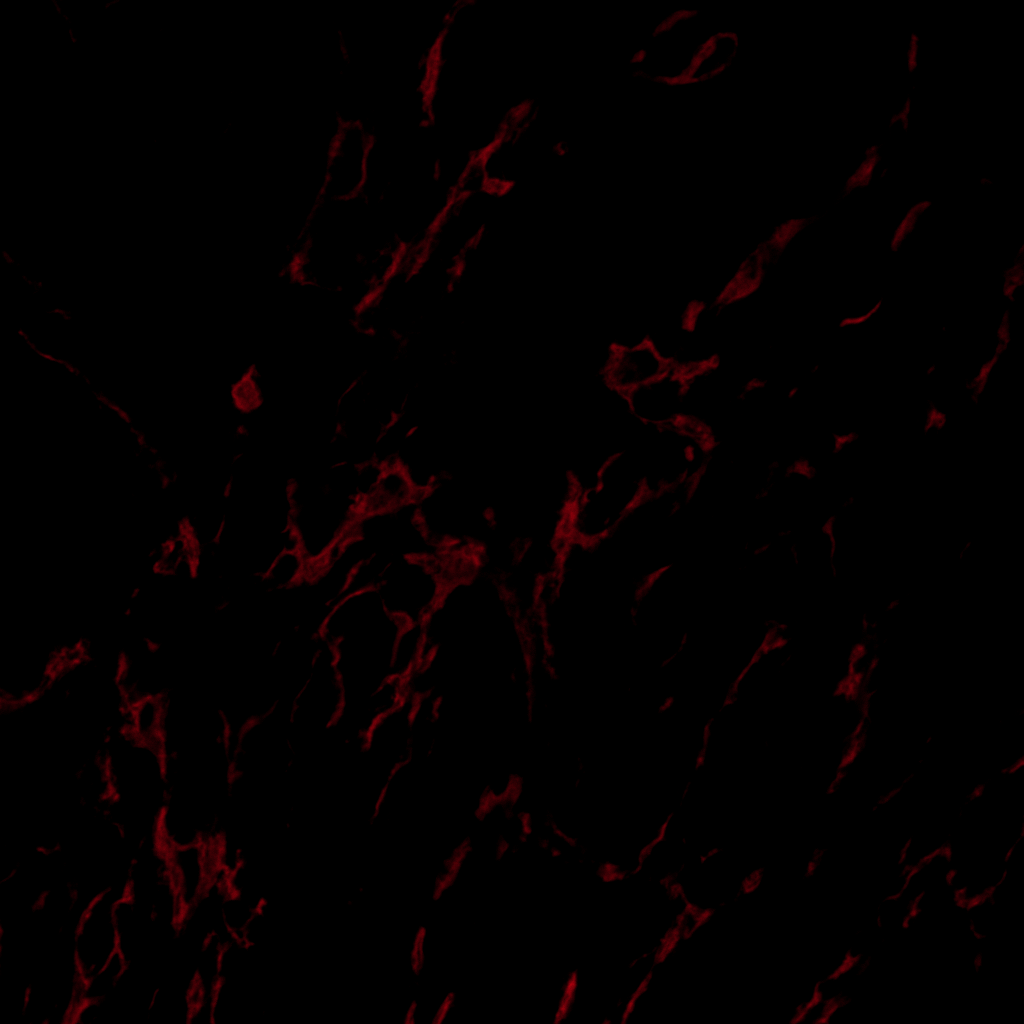

Supplement: Supplementary file 2 [file DataSheet2.zip › IF/p+a-normal-12.11 40x-1-Image Export-04_c1.tif]

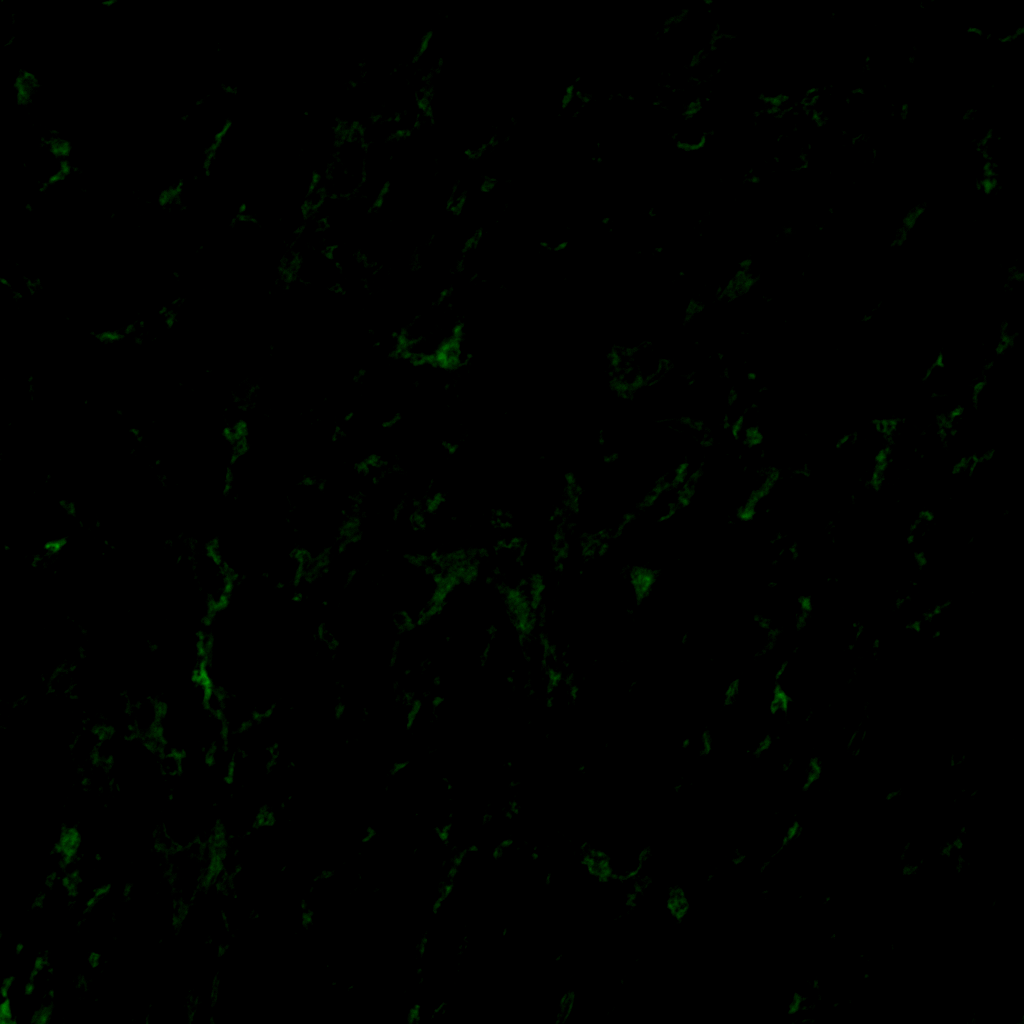

Supplement: Supplementary file 2 [file DataSheet2.zip › IF/p+a-normal-12.11 40x-1-Image Export-04_c2.tif]

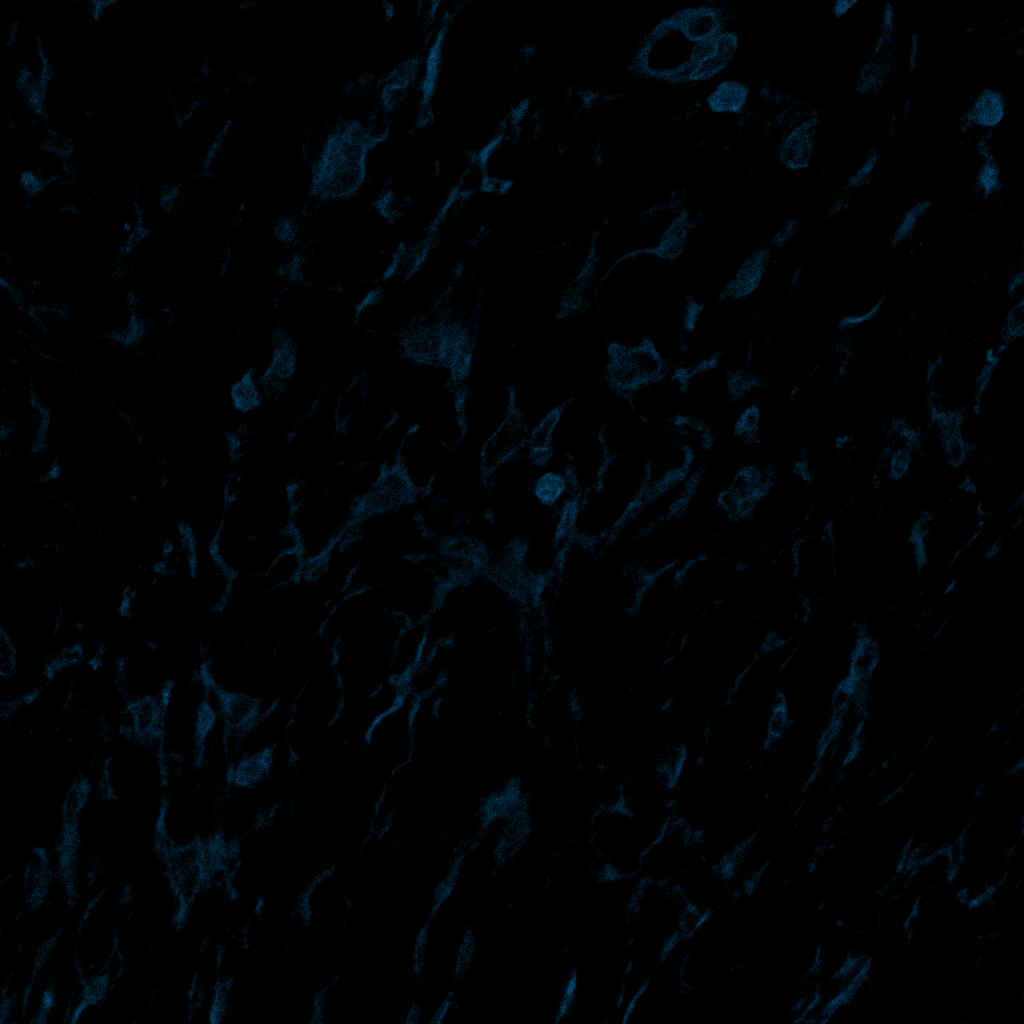

Supplement: Supplementary file 2 [file DataSheet2.zip › IF/p+a-normal-12.11 40x-1-Image Export-04_c3.tif]

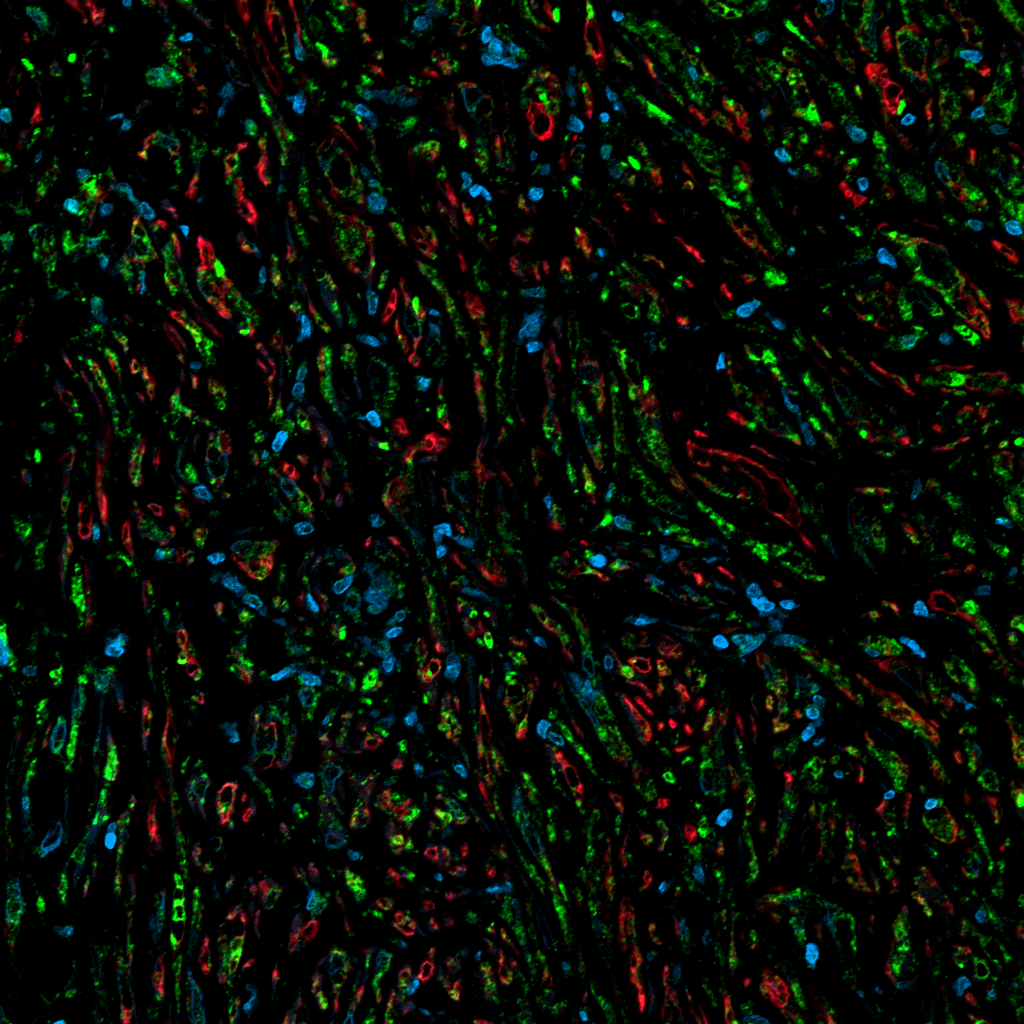

Supplement: Supplementary file 2 [file DataSheet2.zip › IF/p+a-tumor-12.11 20x-2-Image Export-02_c1+2+3.tif]

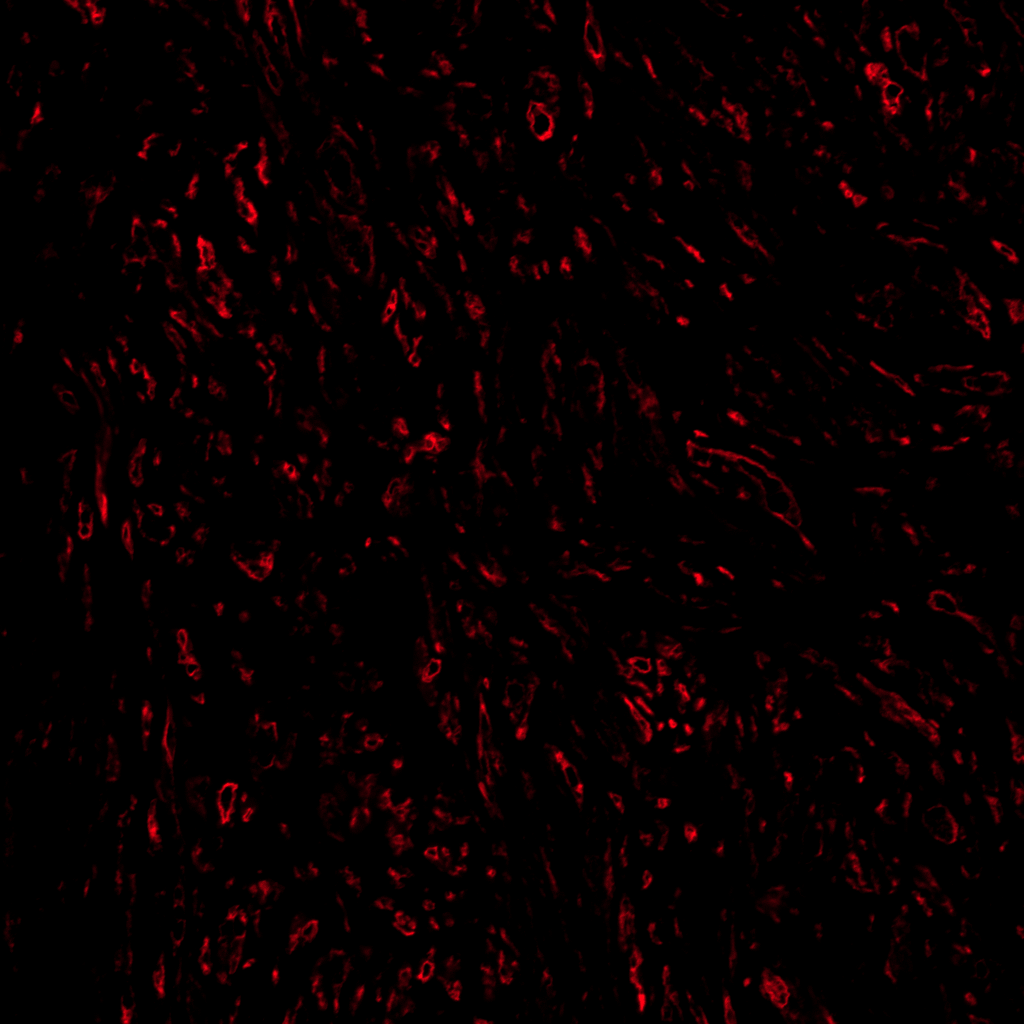

Supplement: Supplementary file 2 [file DataSheet2.zip › IF/p+a-tumor-12.11 20x-2-Image Export-02_c1.tif]

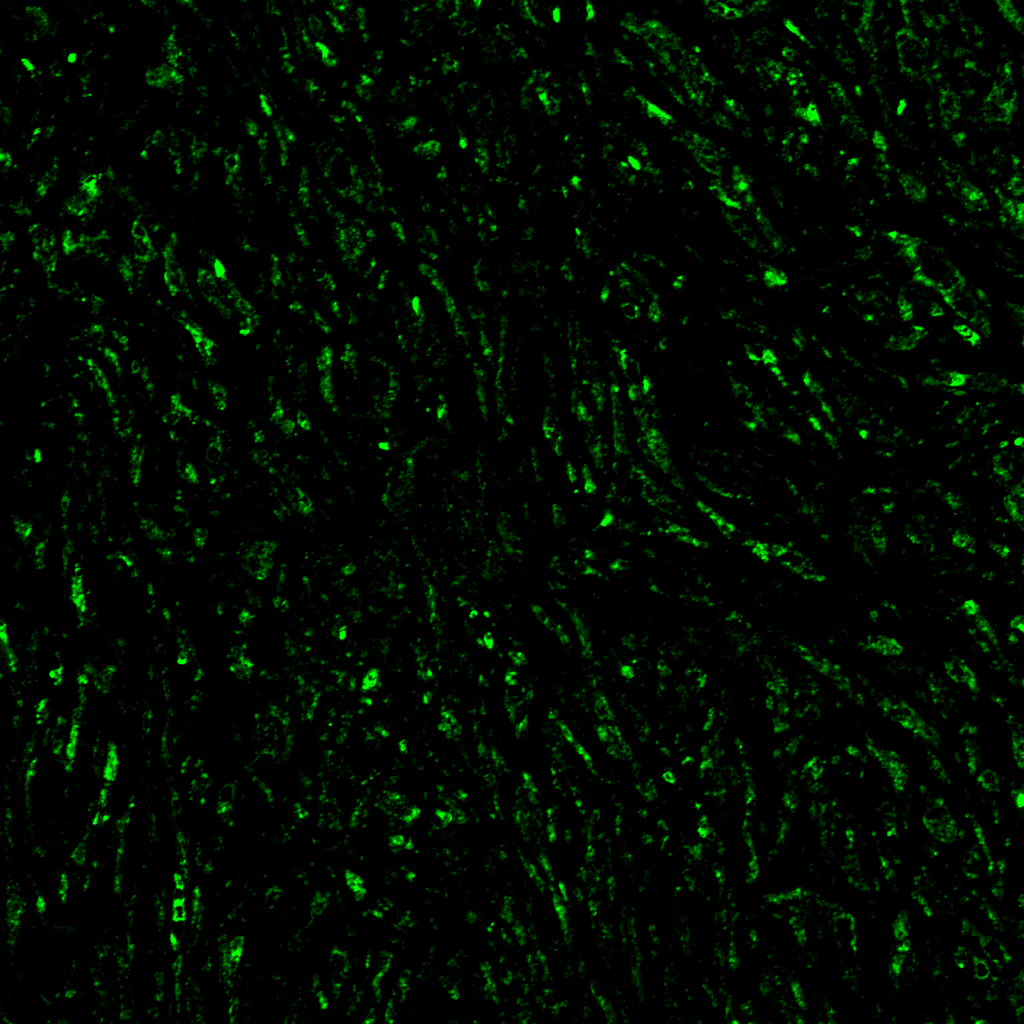

Supplement: Supplementary file 2 [file DataSheet2.zip › IF/p+a-tumor-12.11 20x-2-Image Export-02_c2.tif]

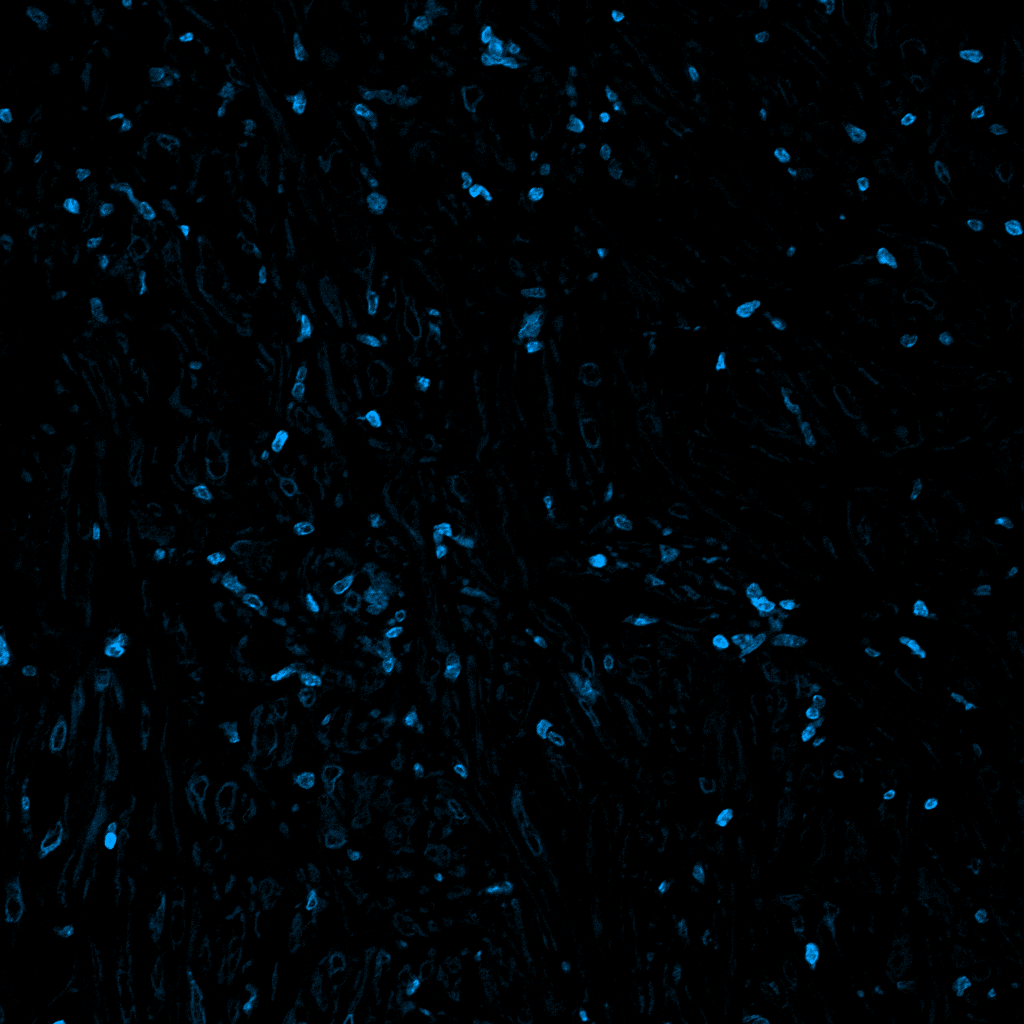

Supplement: Supplementary file 2 [file DataSheet2.zip › IF/p+a-tumor-12.11 20x-2-Image Export-02_c3.tif]

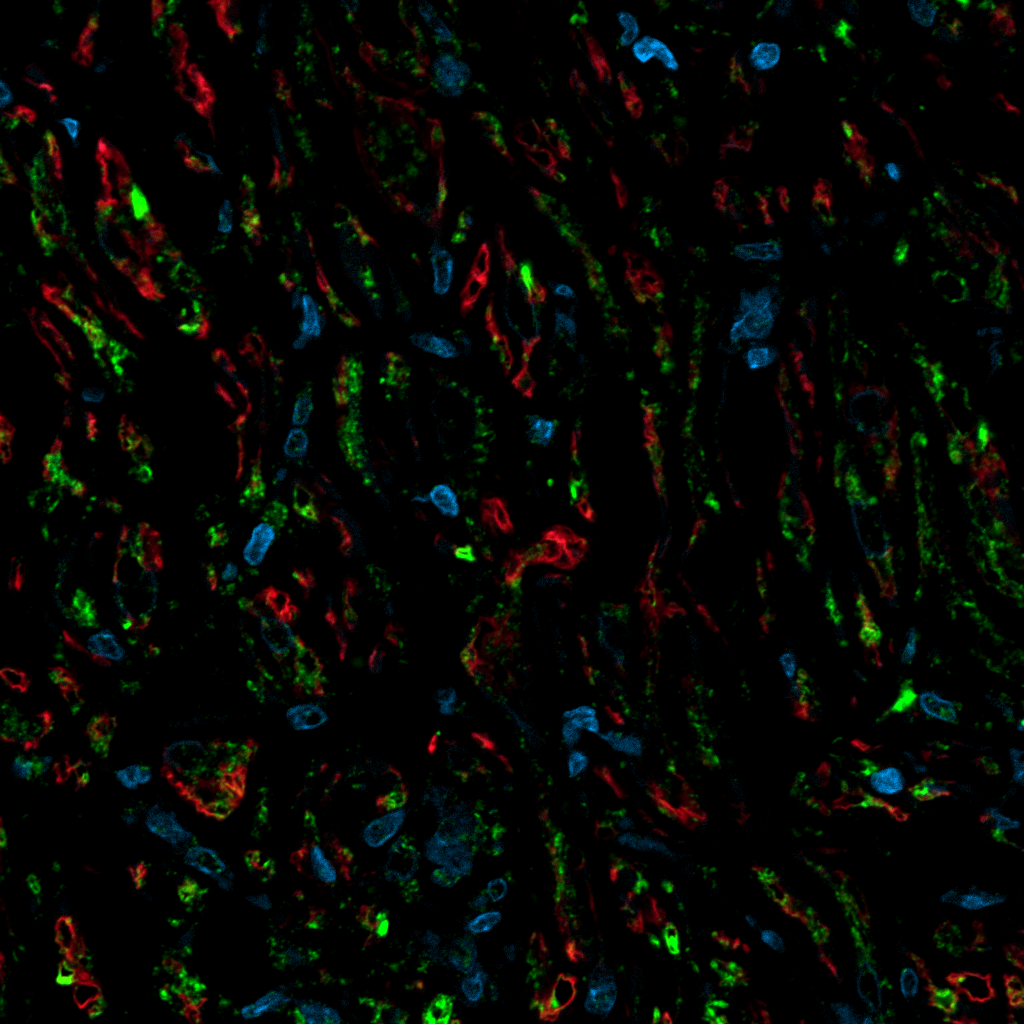

Supplement: Supplementary file 2 [file DataSheet2.zip › IF/p+a-tumor-12.11 40x-1-Image Export-03_c1+2+3.tif]

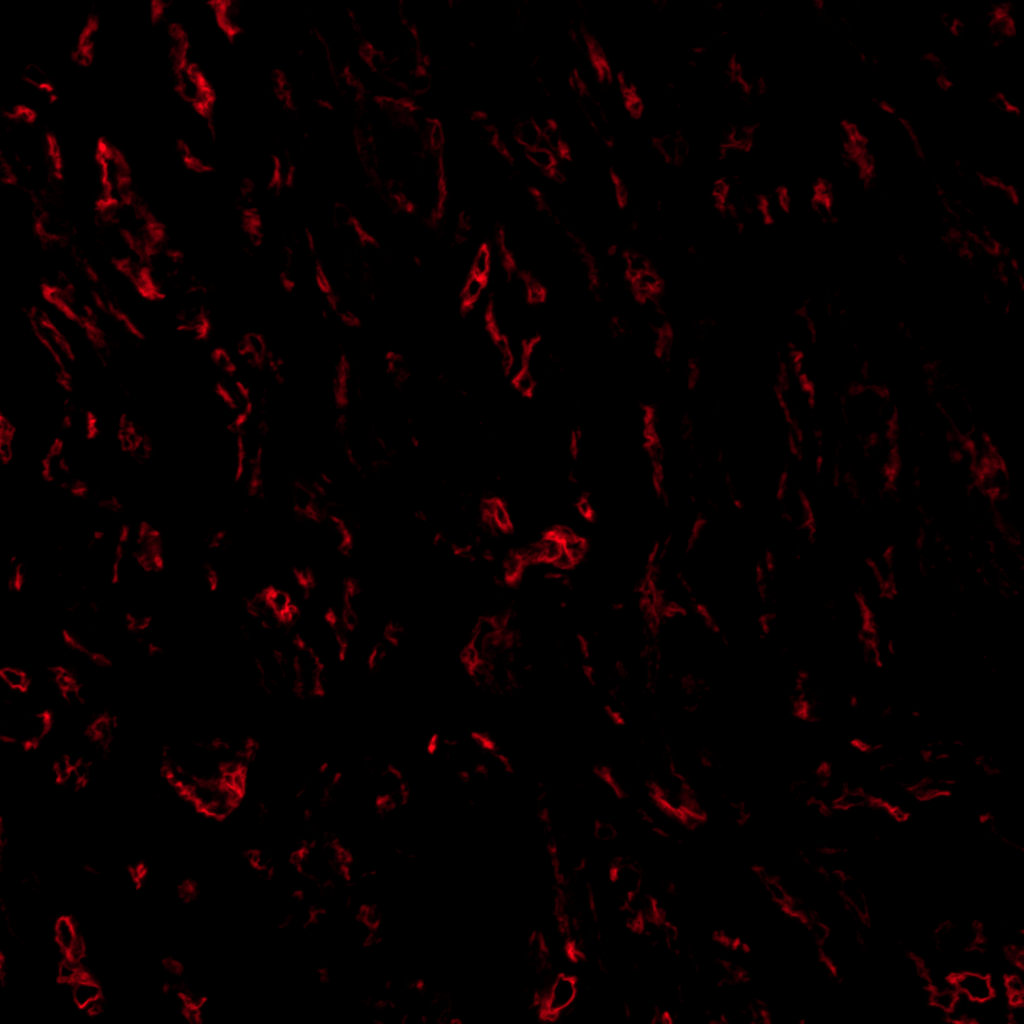

Supplement: Supplementary file 2 [file DataSheet2.zip › IF/p+a-tumor-12.11 40x-1-Image Export-03_c1.tif]

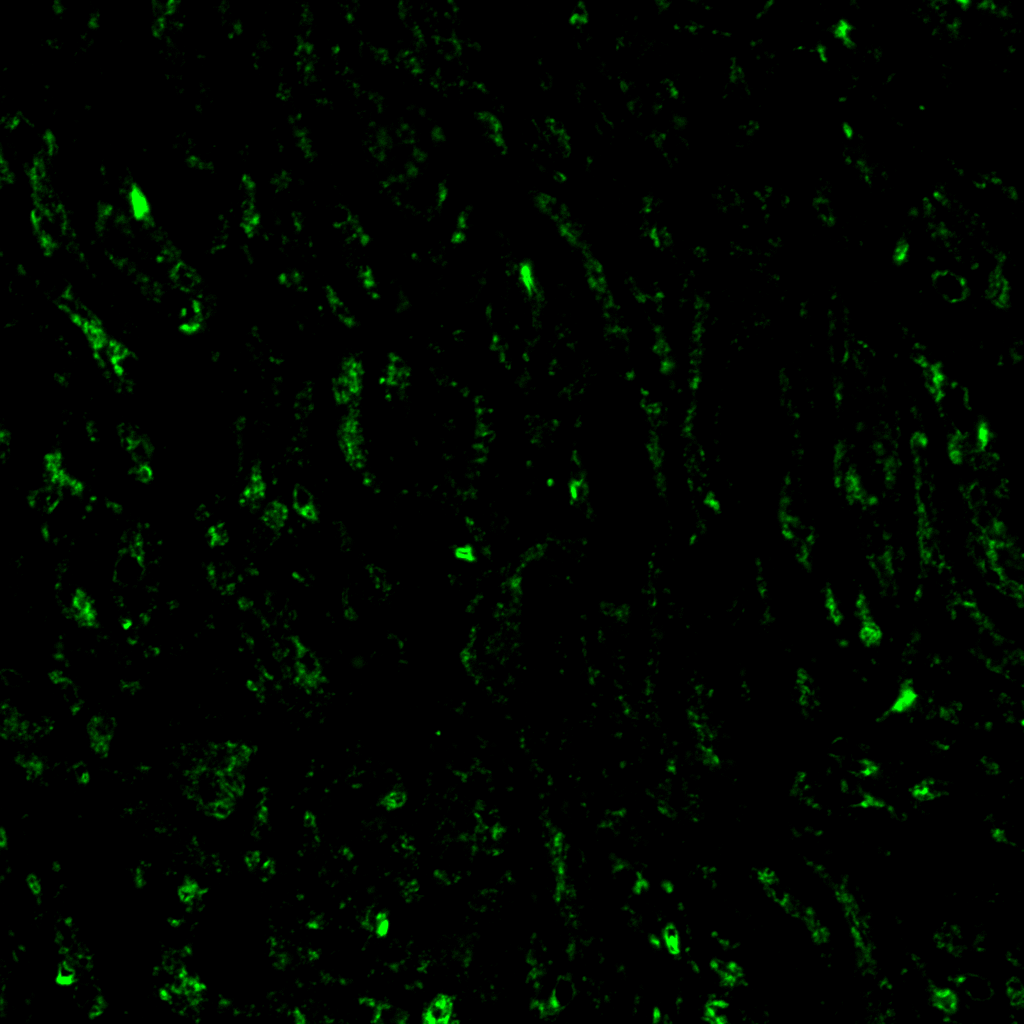

Supplement: Supplementary file 2 [file DataSheet2.zip › IF/p+a-tumor-12.11 40x-1-Image Export-03_c2.tif]

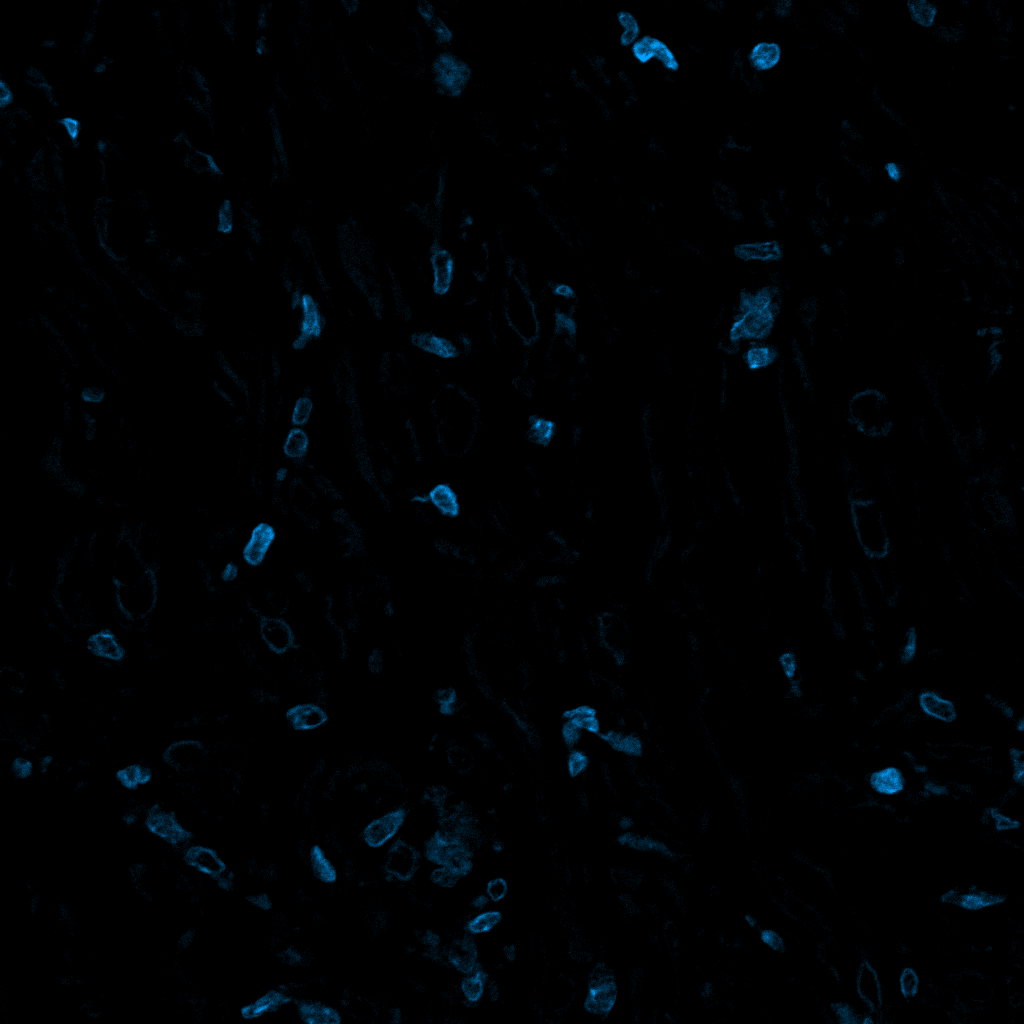

Supplement: Supplementary file 2 [file DataSheet2.zip › IF/p+a-tumor-12.11 40x-1-Image Export-03_c3.tif]

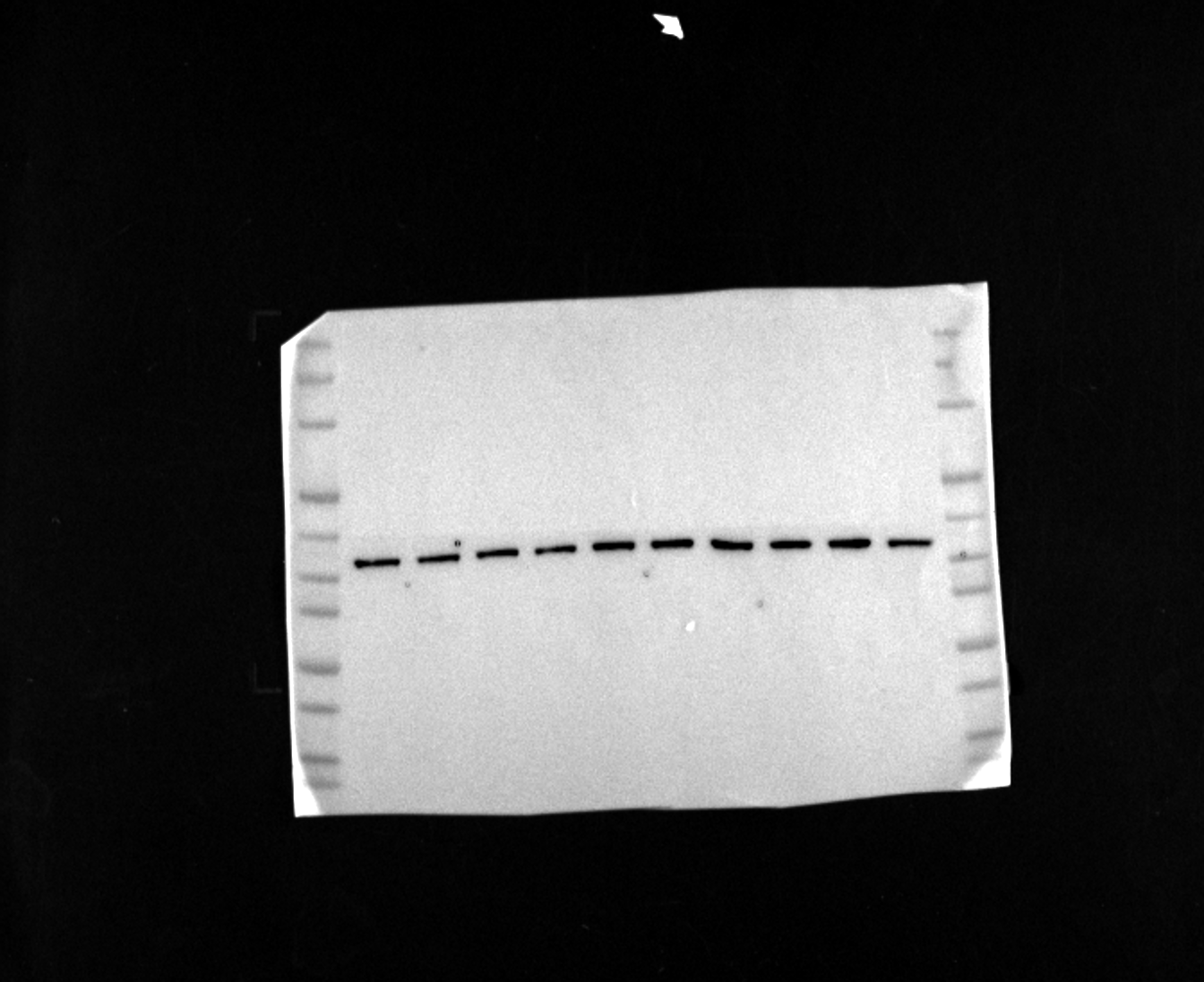

Supplement: Supplementary file 3 [file DataSheet3.zip › PHLDA1-WB/1#-B-ACTIN-M.Tif]

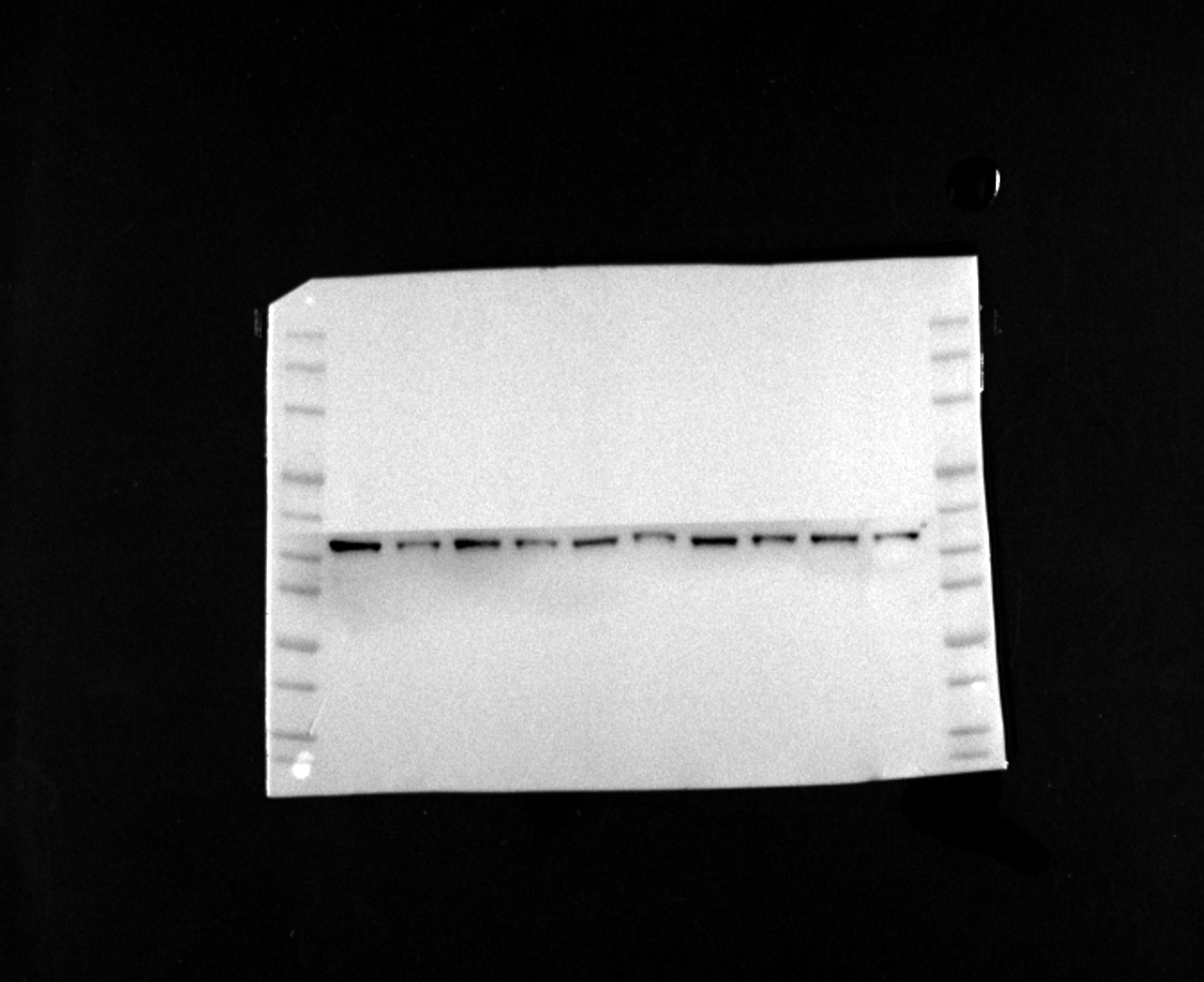

Supplement: Supplementary file 3 [file DataSheet3.zip › PHLDA1-WB/2#-PHLDA1-M.Tif]

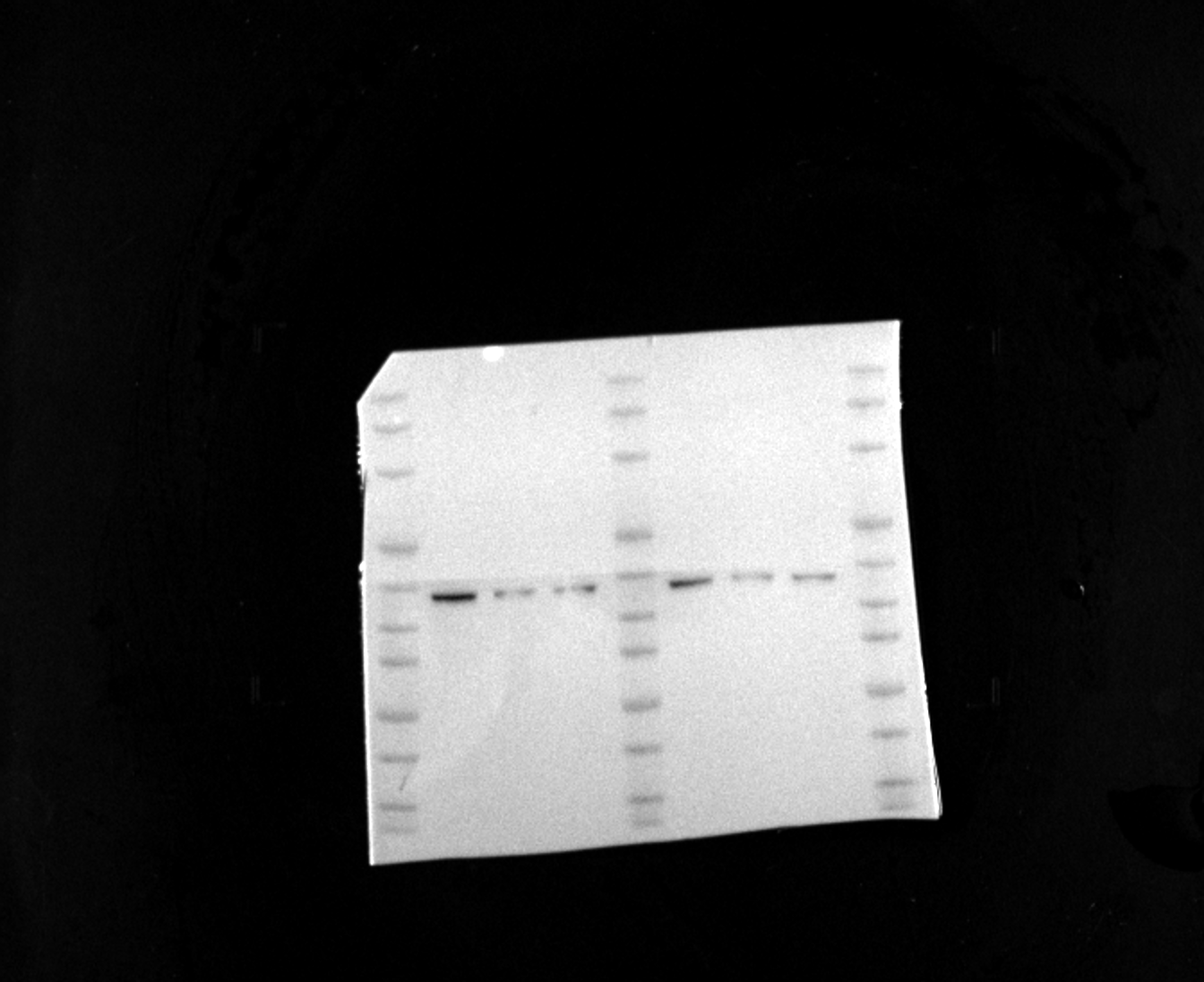

Supplement: Supplementary file 3 [file DataSheet3.zip › PHLDA1-WB/3#-PHLDA1-M.Tif]

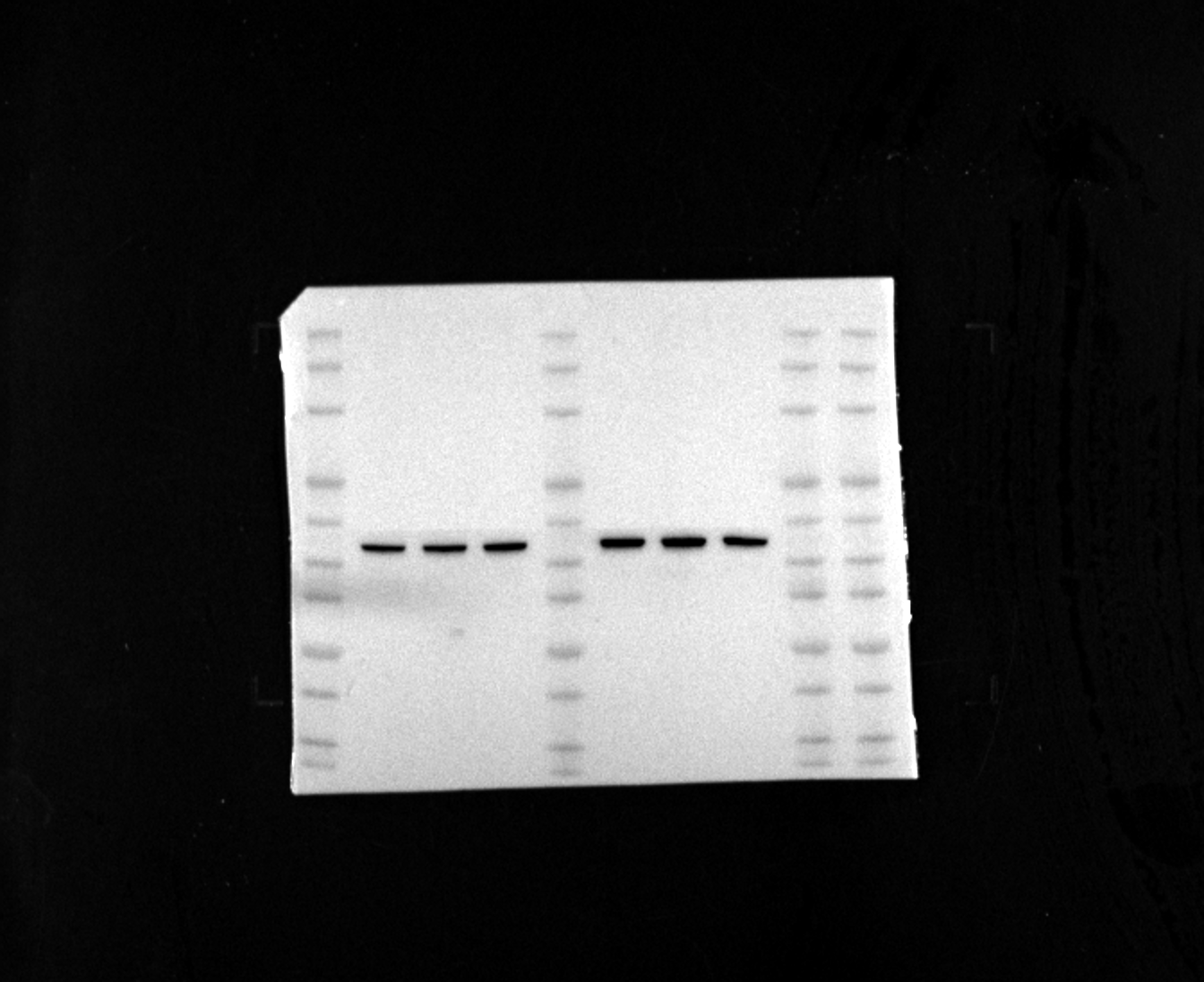

Supplement: Supplementary file 3 [file DataSheet3.zip › PHLDA1-WB/4#-B-ACTIN-M.Tif]

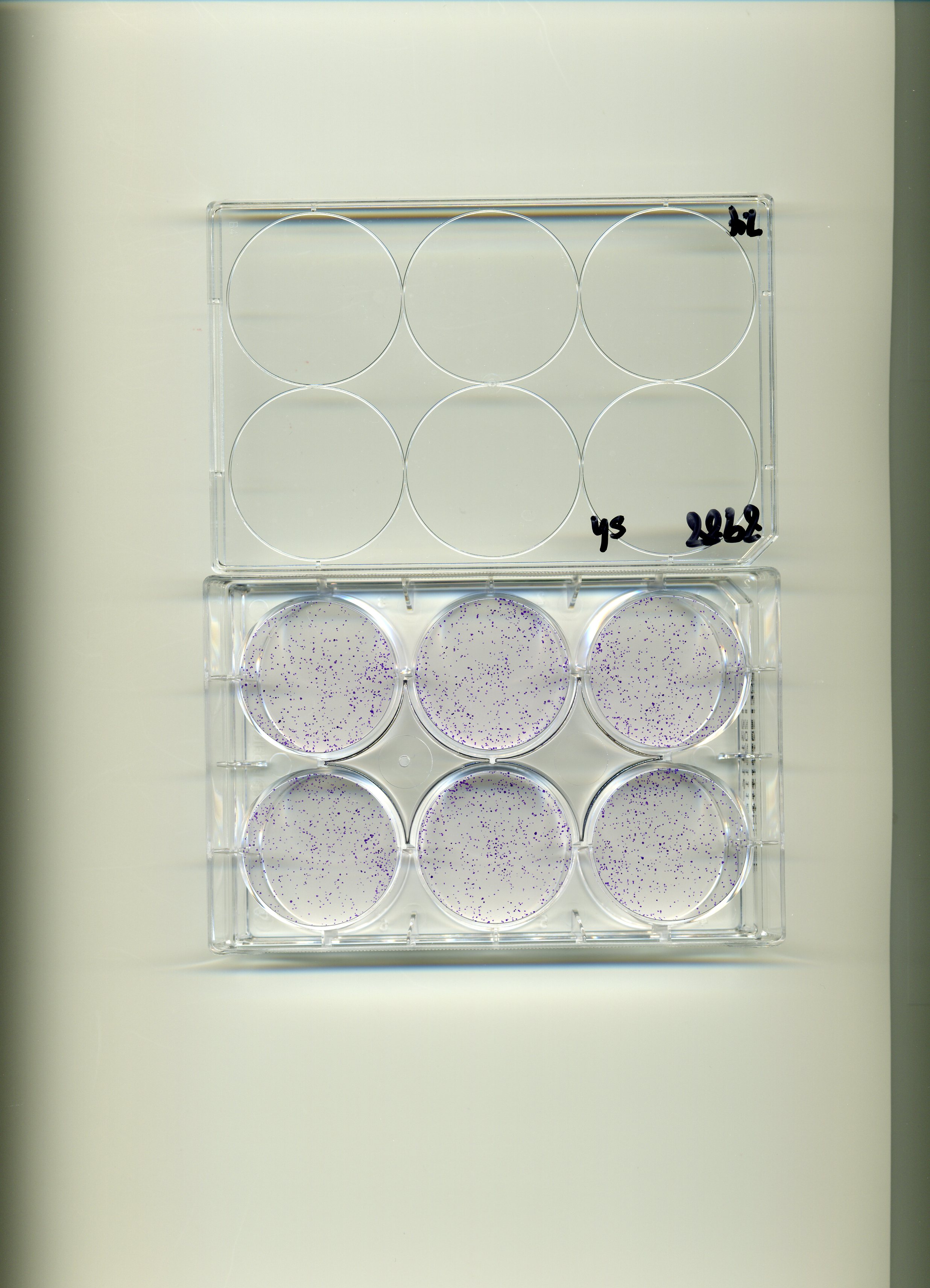

Supplement: Supplementary file 4 [file DataSheet4.zip › Plate cloning experiment/8988/8988 SH-1-2.jpg]

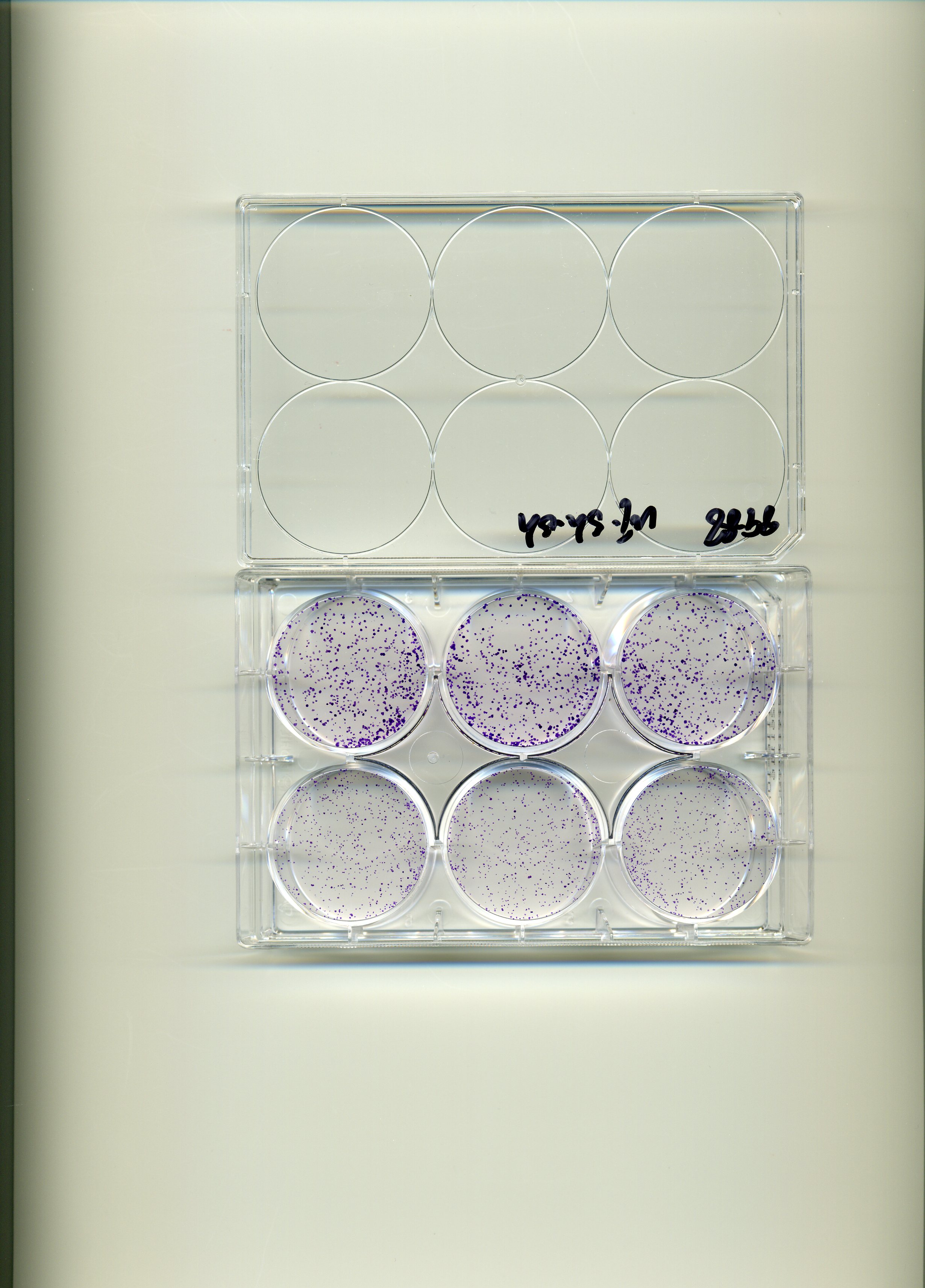

Supplement: Supplementary file 4 [file DataSheet4.zip › Plate cloning experiment/8988/8988 WT-SH-SH-2.jpg]

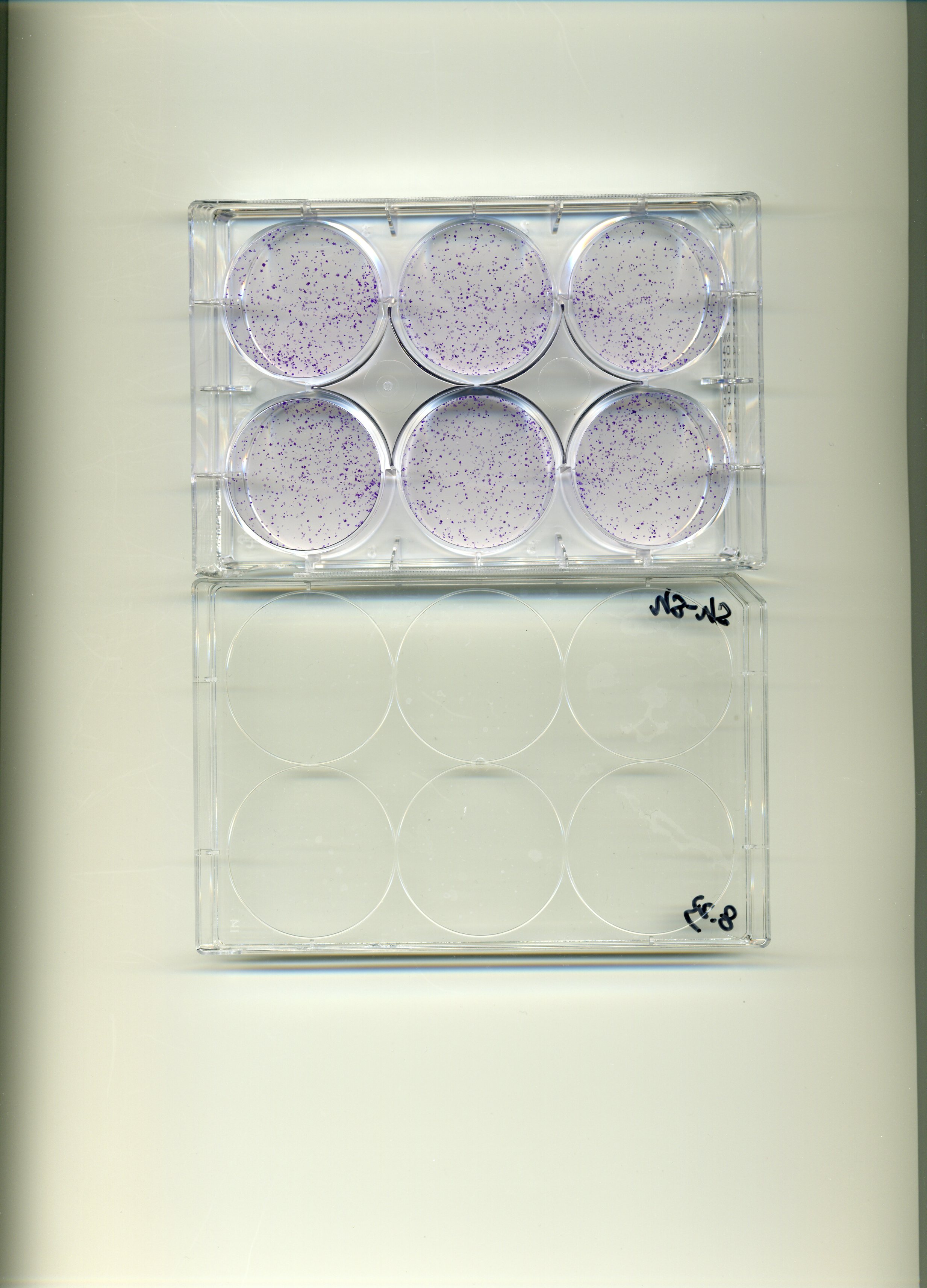

Supplement: Supplementary file 4 [file DataSheet4.zip › Plate cloning experiment/Panc1/panc1 SH-SH-4.jpg]

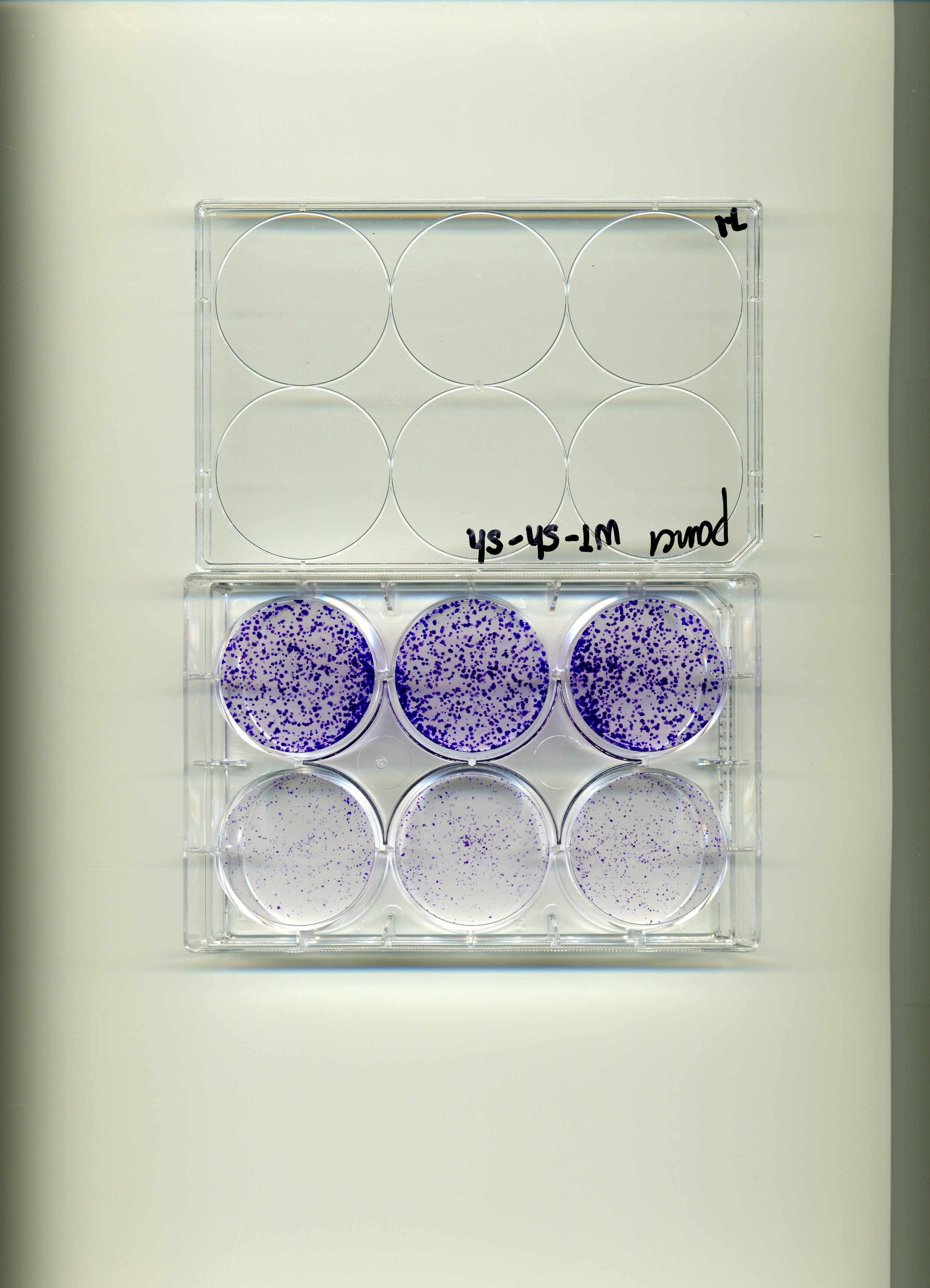

Supplement: Supplementary file 4 [file DataSheet4.zip › Plate cloning experiment/Panc1/panc1 WT-SH-SH-7.jpg]

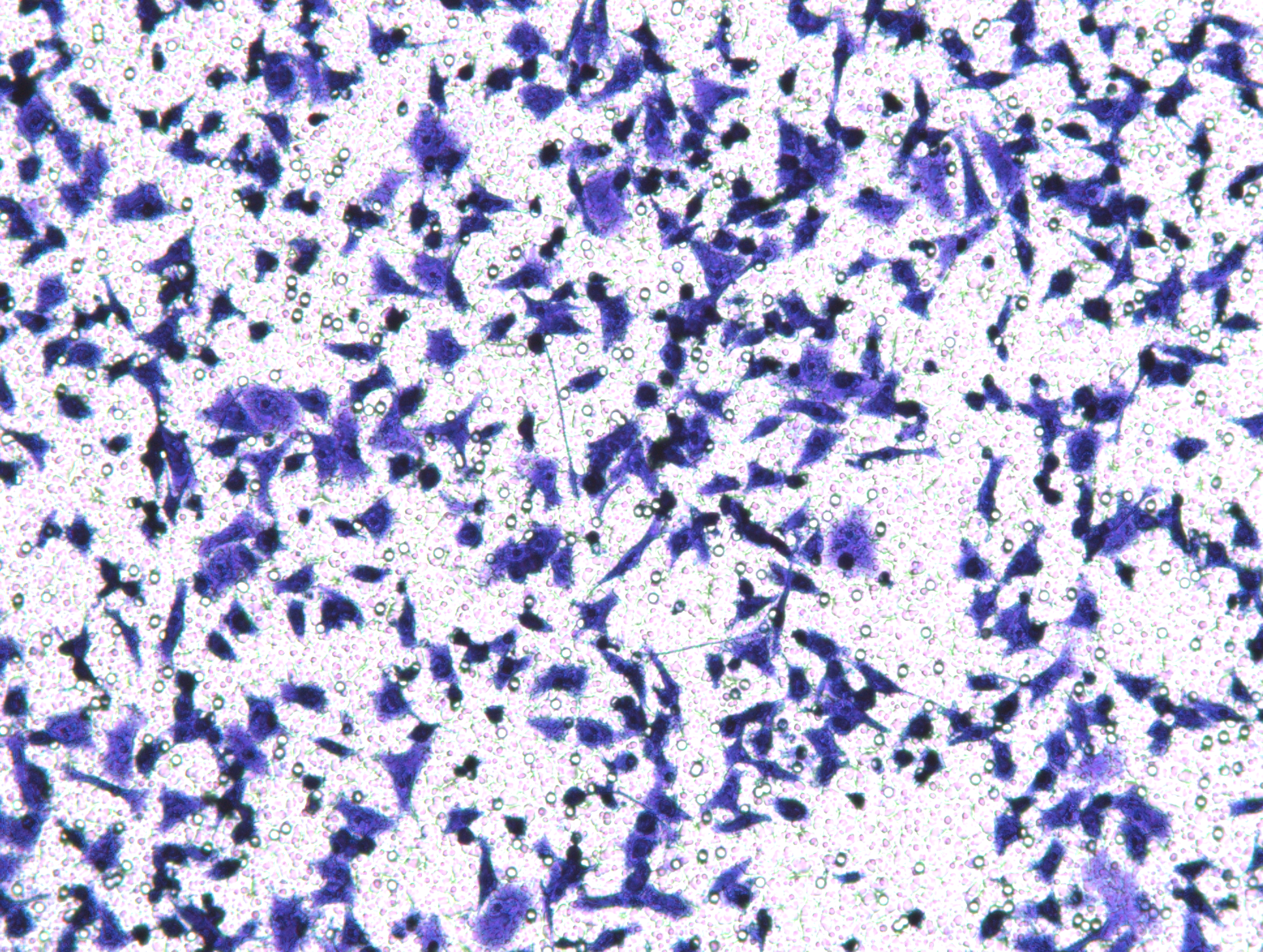

Supplement: Supplementary file 5 [file DataSheet5.zip › Panc1/14-5-图像导出-102.tif]

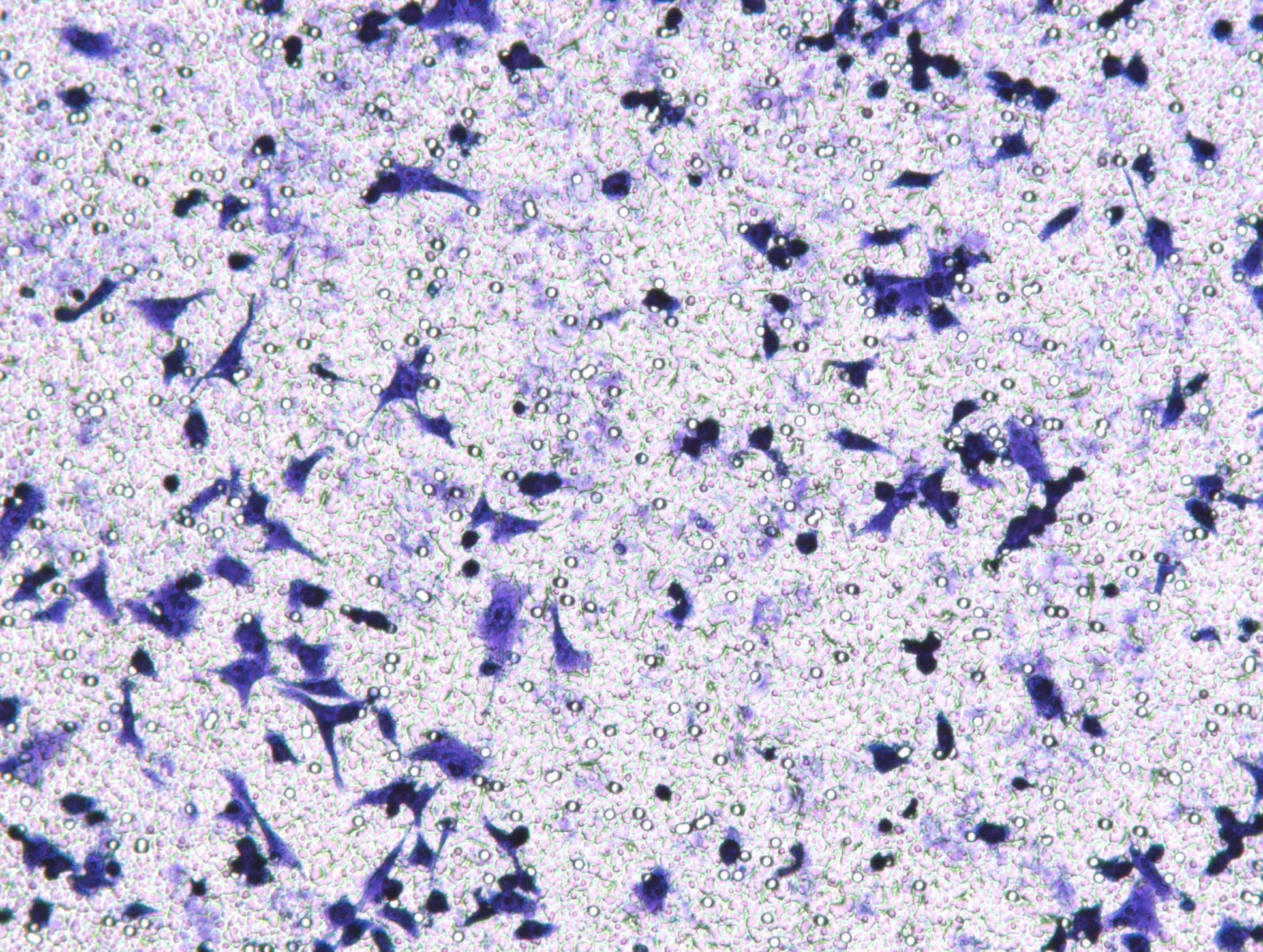

Supplement: Supplementary file 5 [file DataSheet5.zip › Panc1/19-1-图像导出-131.tif]

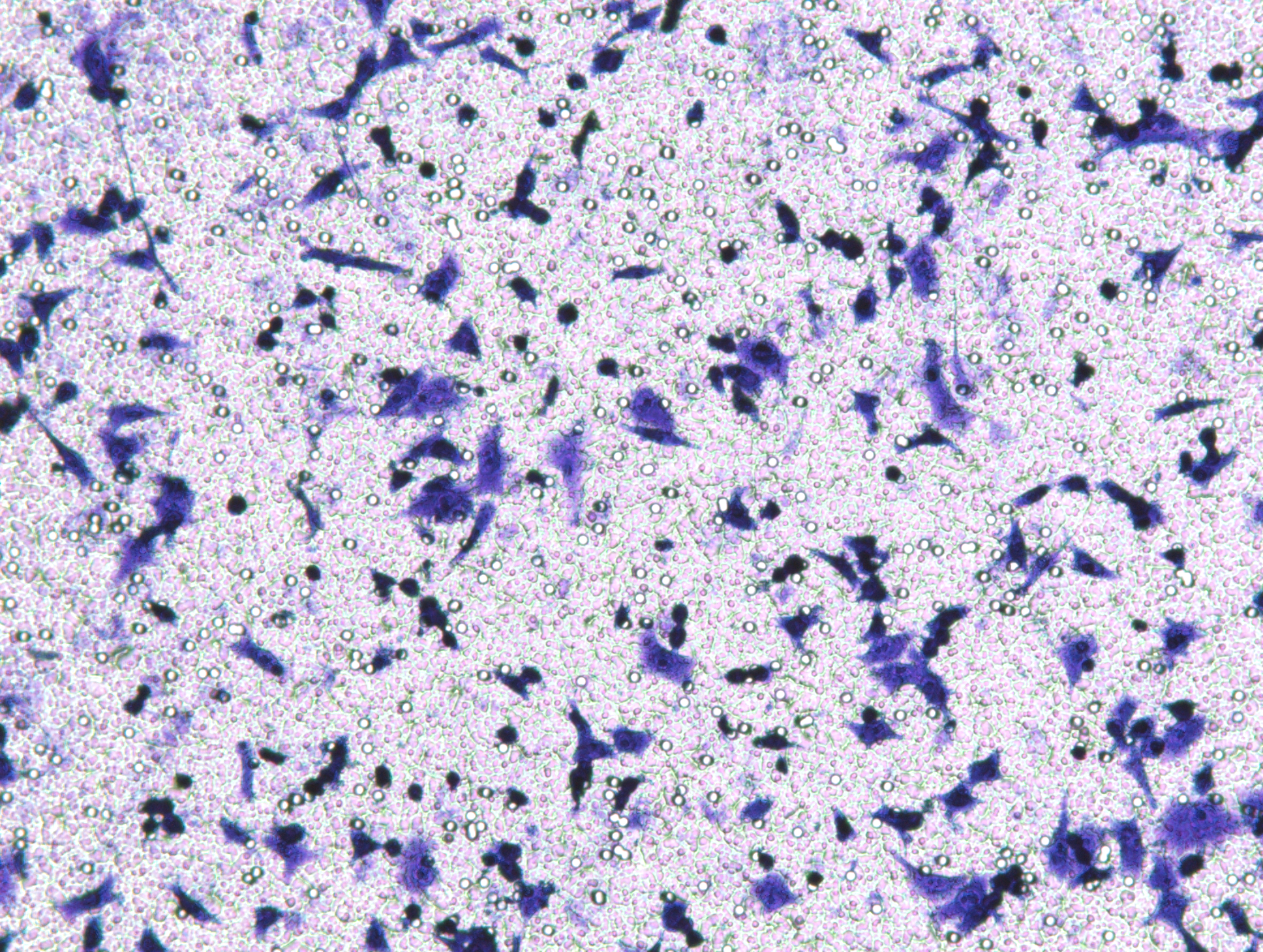

Supplement: Supplementary file 5 [file DataSheet5.zip › Panc1/19-2-图像导出-132.tif]

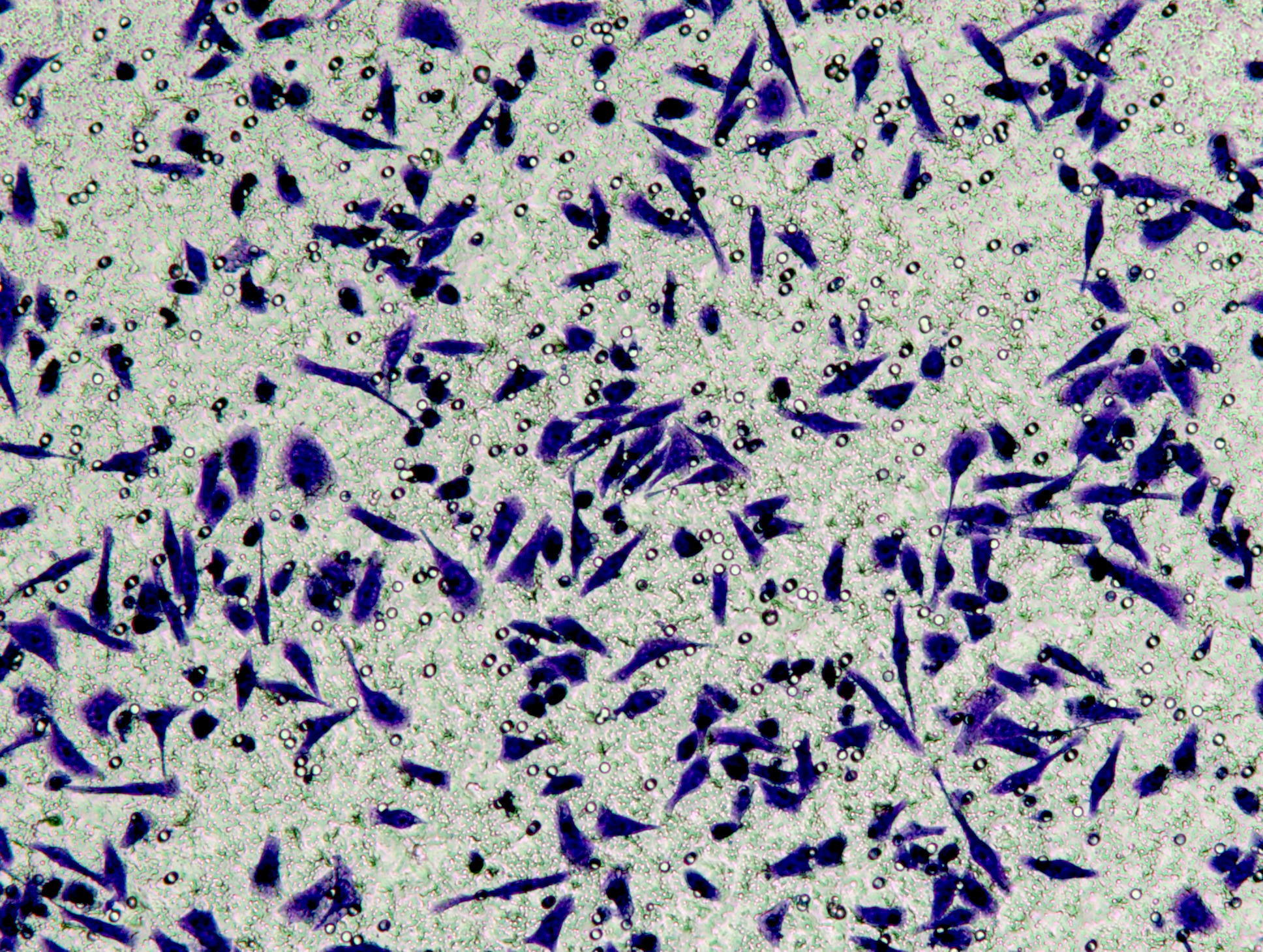

Supplement: Supplementary file 6 [file DataSheet6.zip › 8988/2-11-图像导出-25.tif]

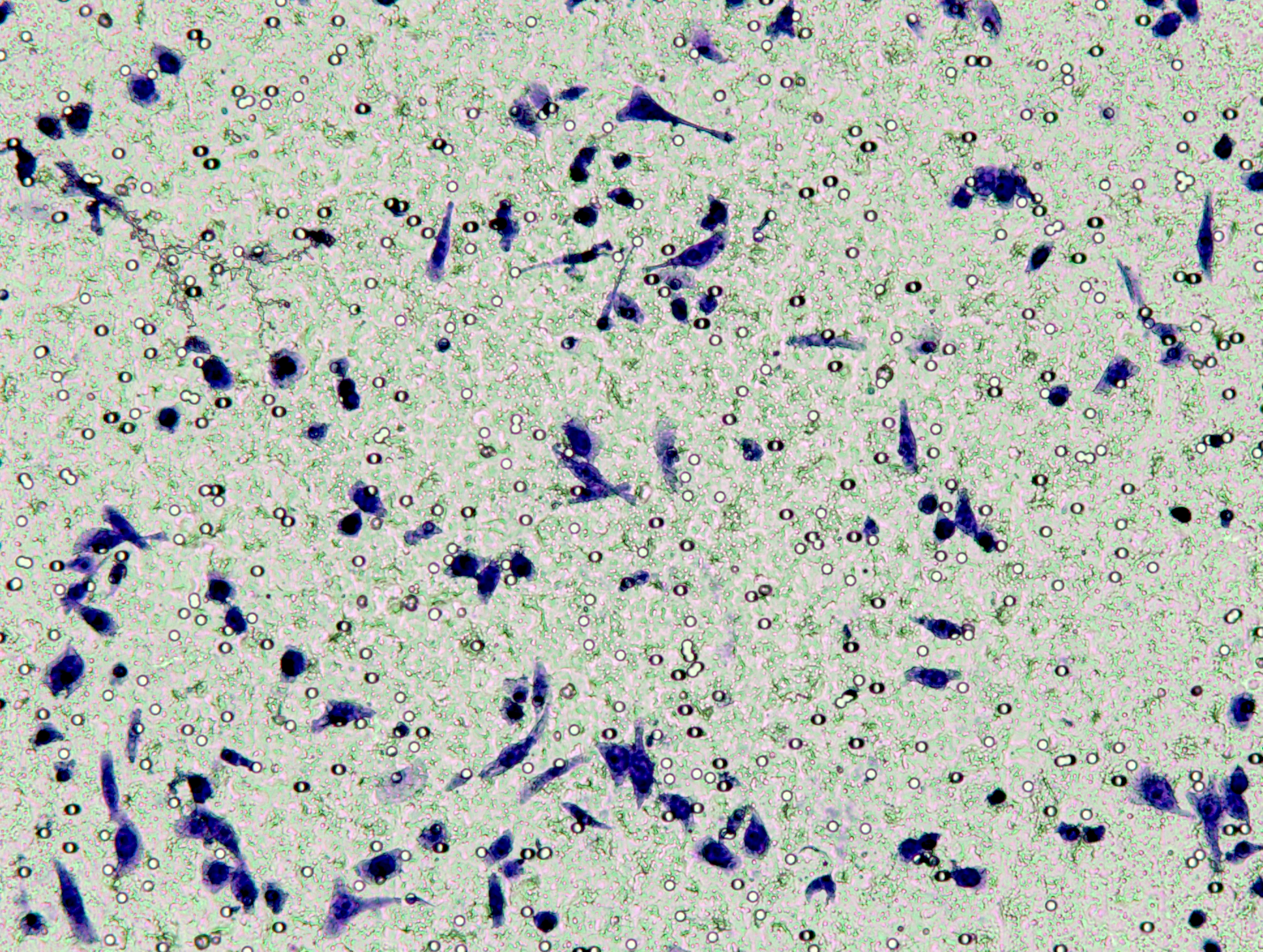

Supplement: Supplementary file 6 [file DataSheet6.zip › 8988/3-10-图像导出-36.tif]

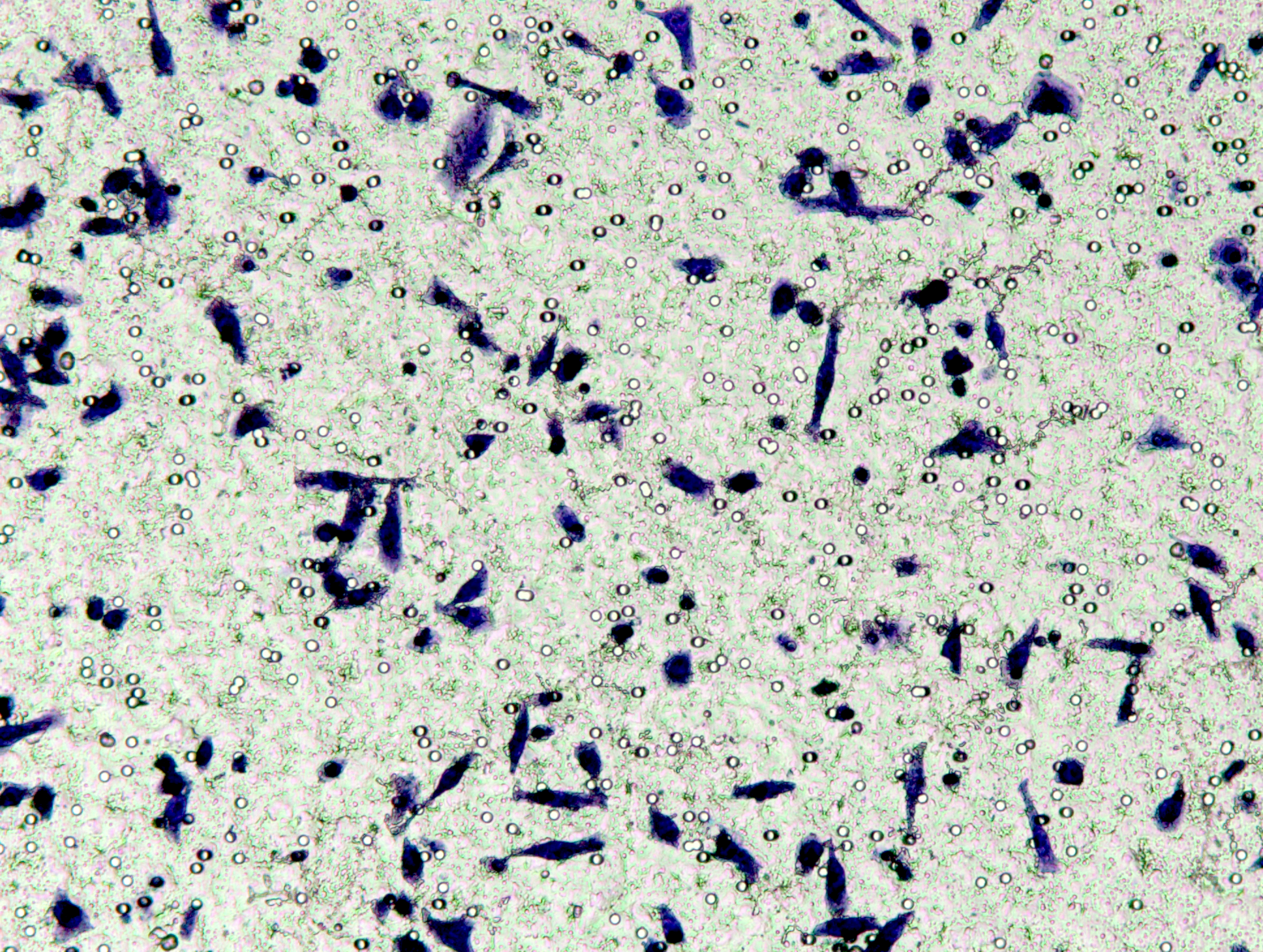

Supplement: Supplementary file 6 [file DataSheet6.zip › 8988/3-11-图像导出-37.tif]

# Number of interactions

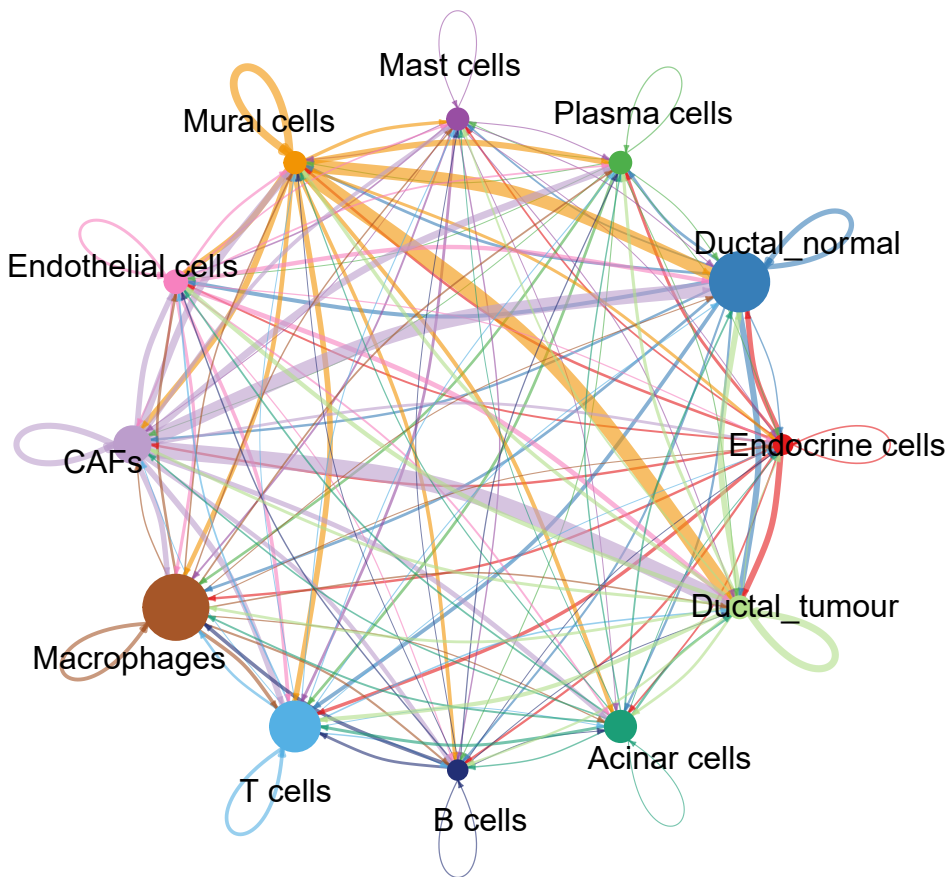

Supplement: Supplementary file 7 [file DataSheet7.zip › raw data/2-cellchat/1.1_Cellchat-Interaction number.pdf]

# Interaction weights/strength

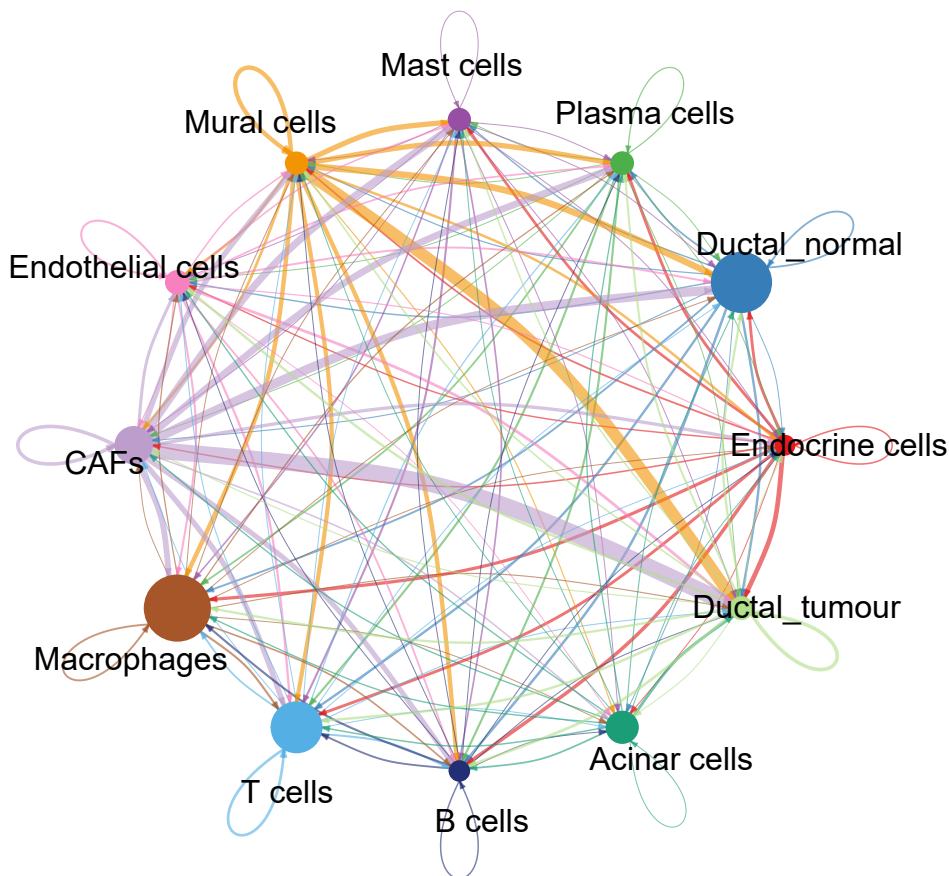

Supplement: Supplementary file 7 [file DataSheet7.zip › raw data/2-cellchat/1.1_Cellchat-Interaction weights.pdf]

Outgoing signaling patterns

Incoming signaling patterns

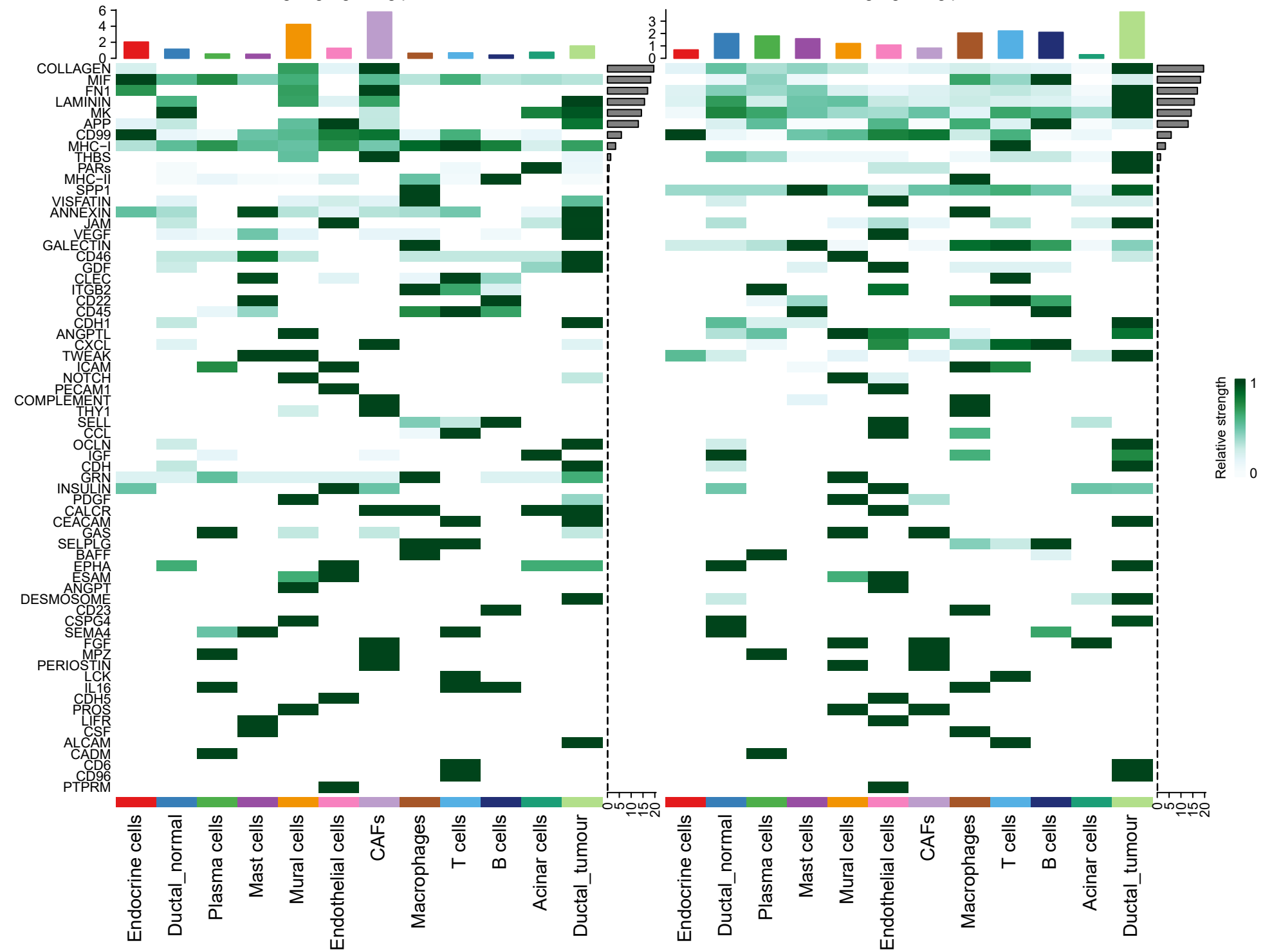

Supplement: Supplementary file 7 [file DataSheet7.zip › raw data/2-cellchat/1.2_signalingRole.pdf]

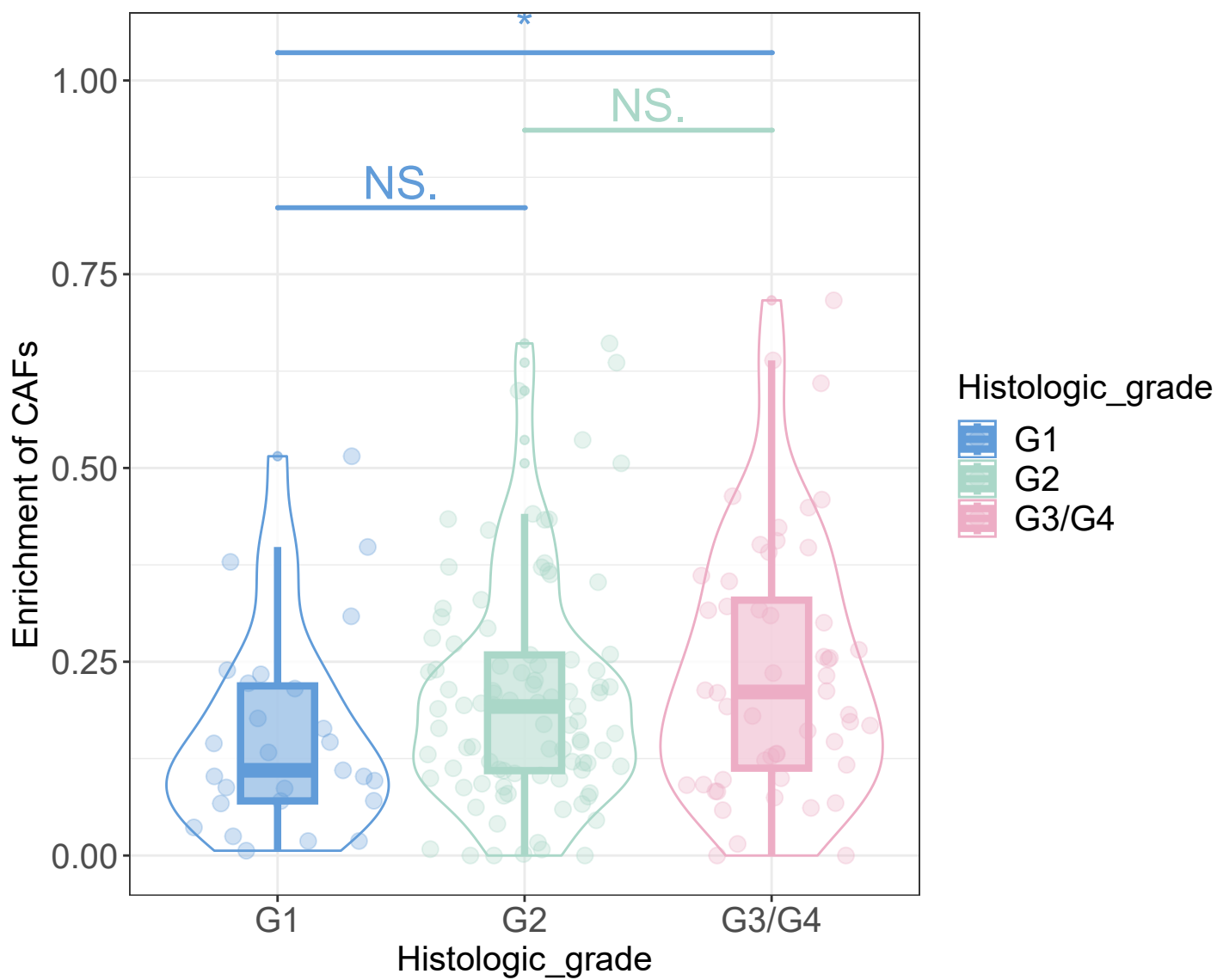

Supplement: Supplementary file 7 [file DataSheet7.zip › raw data/2-cellchat/2.2_Histologic_grade.pdf]

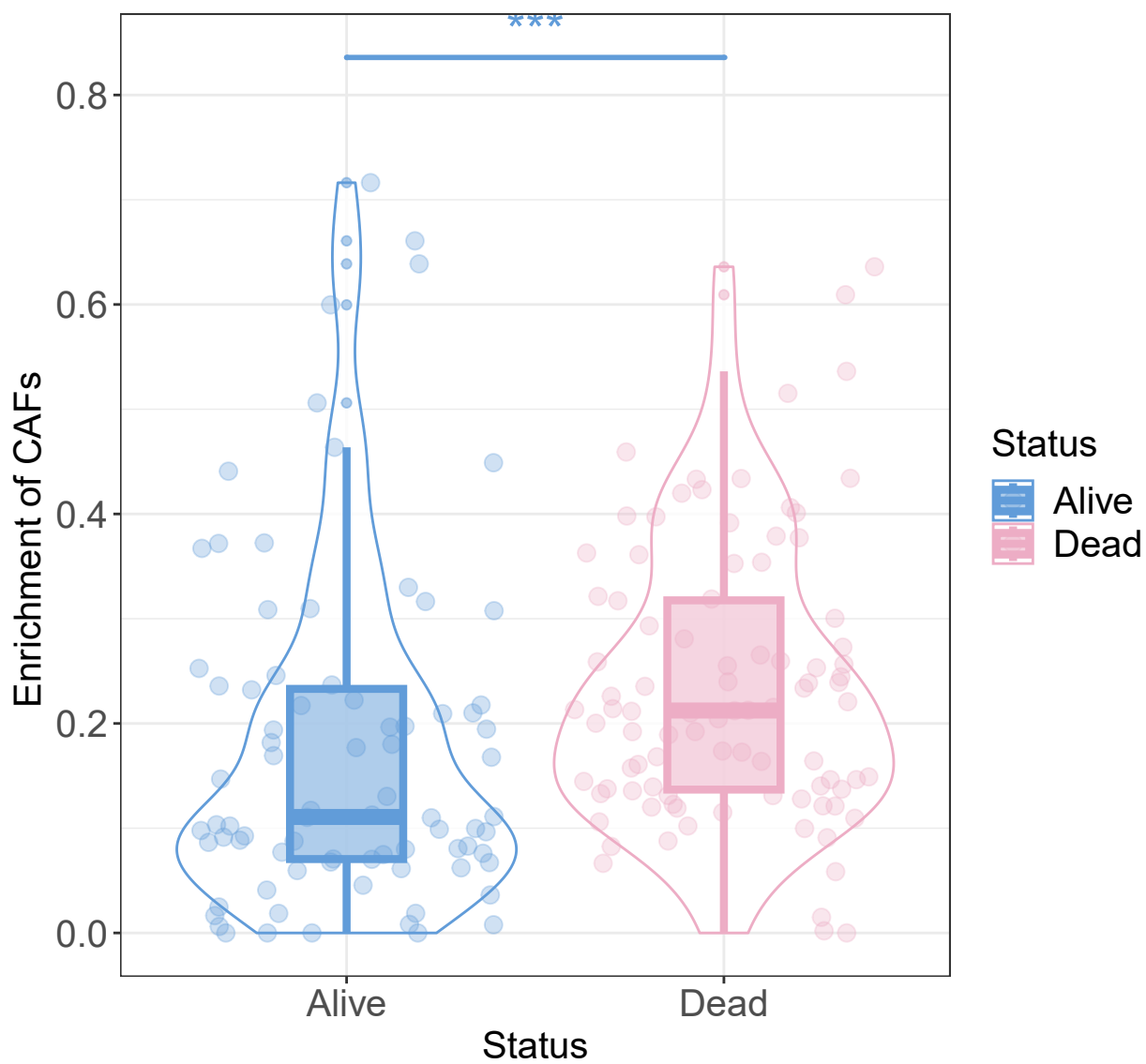

Supplement: Supplementary file 7 [file DataSheet7.zip › raw data/2-cellchat/2.2_Status.pdf]

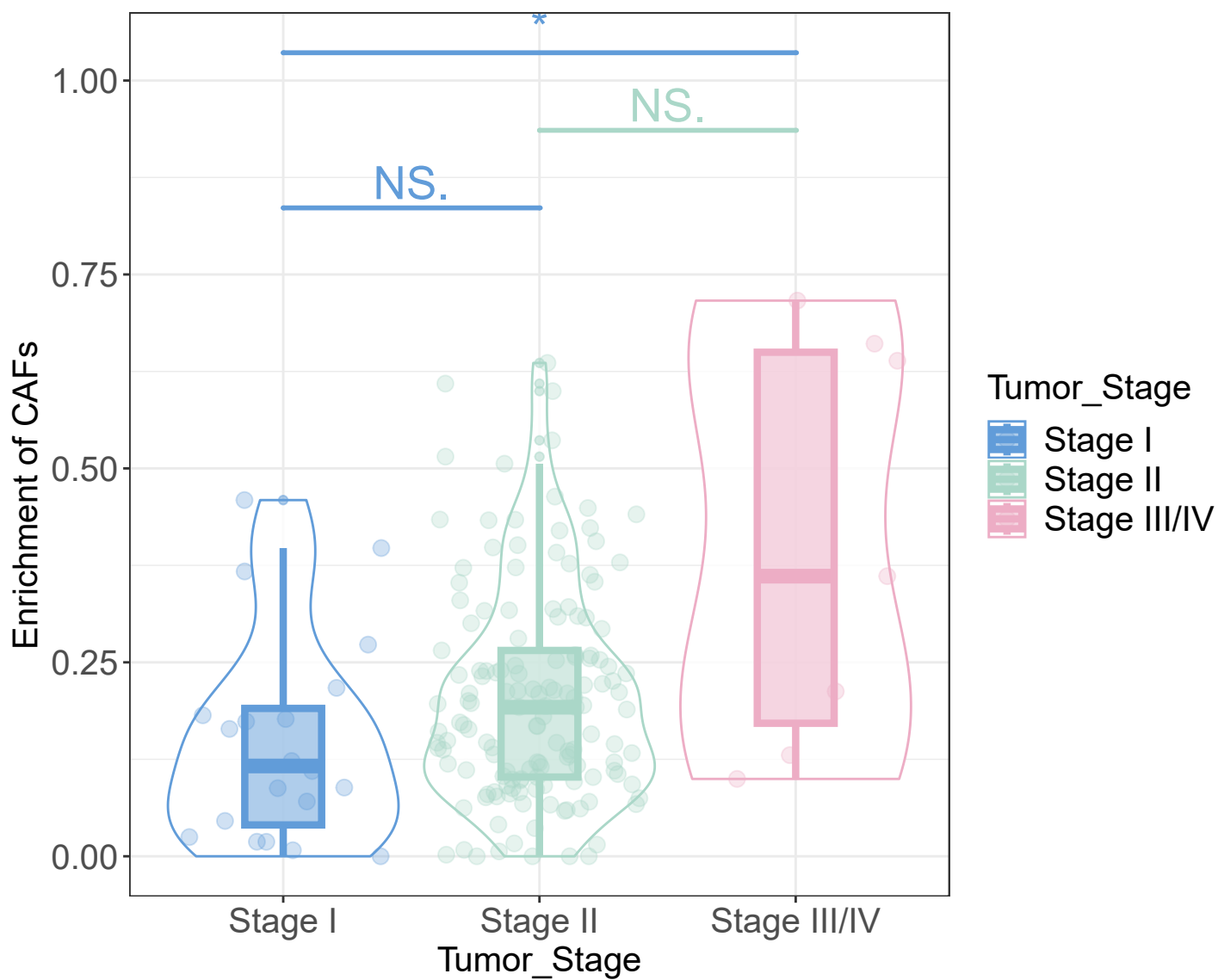

Supplement: Supplementary file 7 [file DataSheet7.zip › raw data/2-cellchat/2.2_Tumor_Stage.pdf]

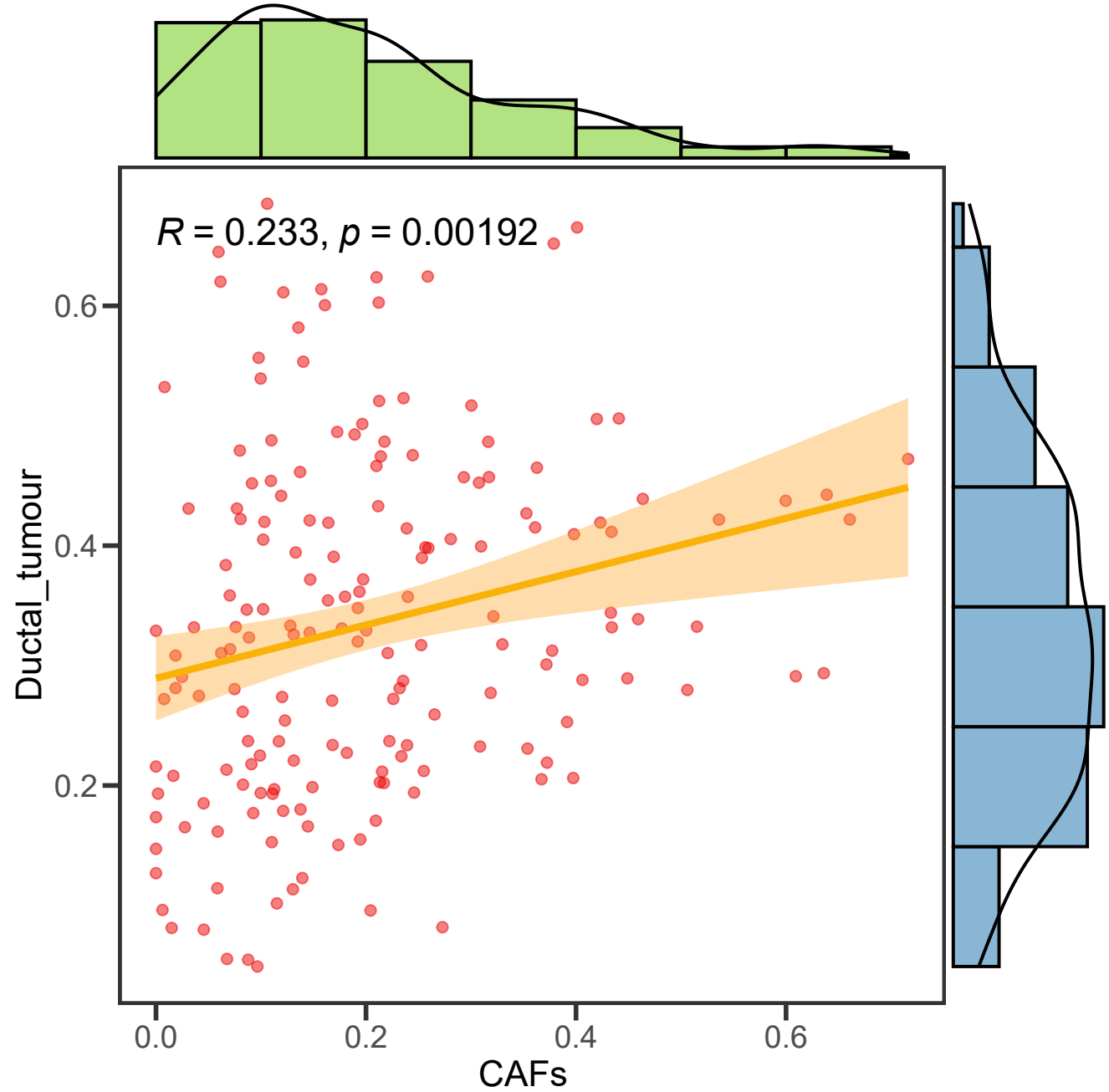

Supplement: Supplementary file 7 [file DataSheet7.zip › raw data/2-cellchat/2.3_cor.pdf]

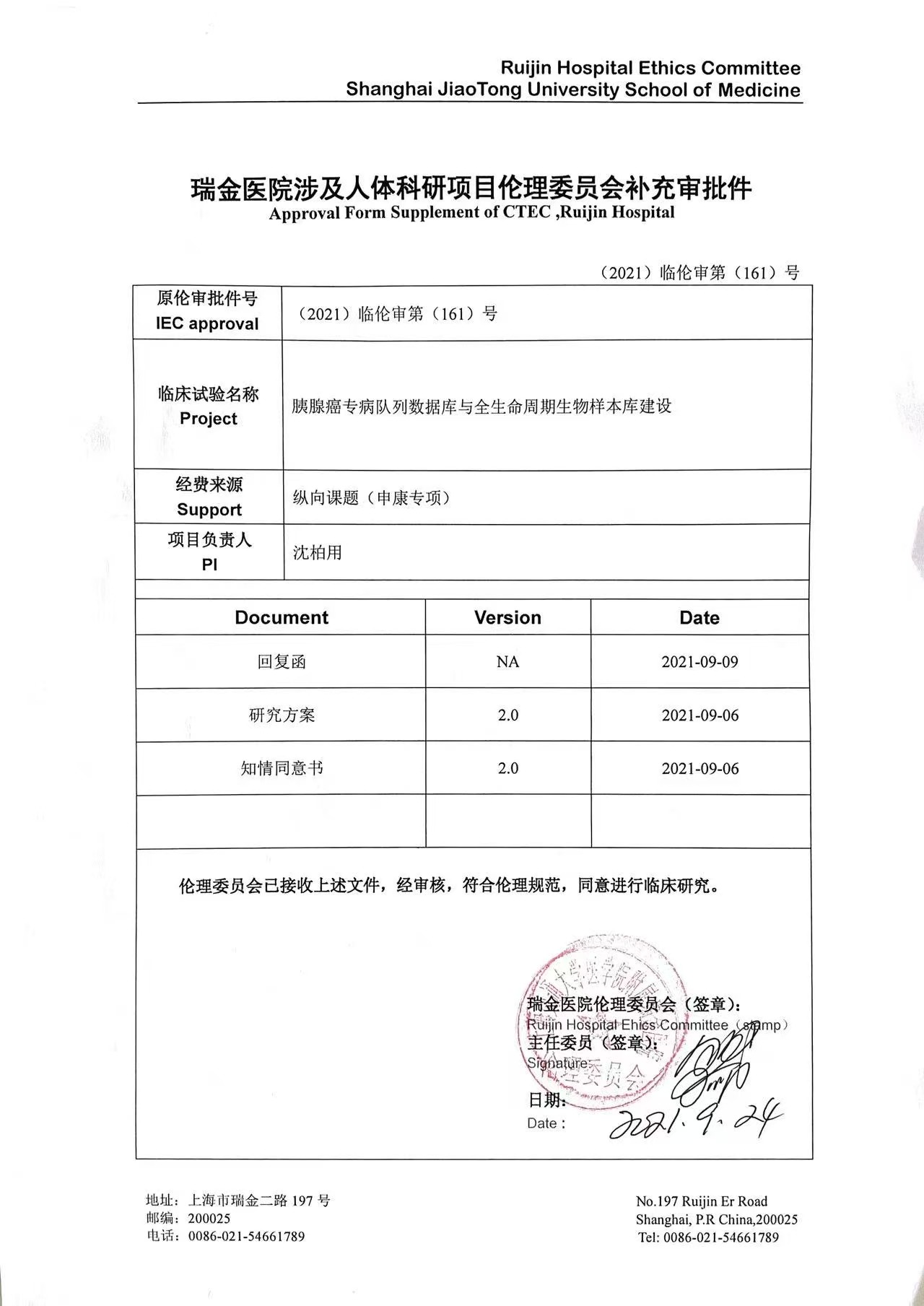

Supplement: Supplementary file 9 [file Image1.jpeg]
