# Supplementary material for: Integrated multiomics analysis identifies PHLDA1+ fibroblasts as prognostic biomarkers and mediators of biological functions in pancreatic cancer
Source: Front Immunol. 2025 Jul 4;16:1592416. doi: 10.3389/fimmu.2025.1592416 (PMC12271128; doi:10.3389/fimmu.2025.1592416)
Supplement: Supplementary file 7 [file DataSheet7.zip › raw data/2-cellchat/2.1_Fibroblasts.survival.pdf]

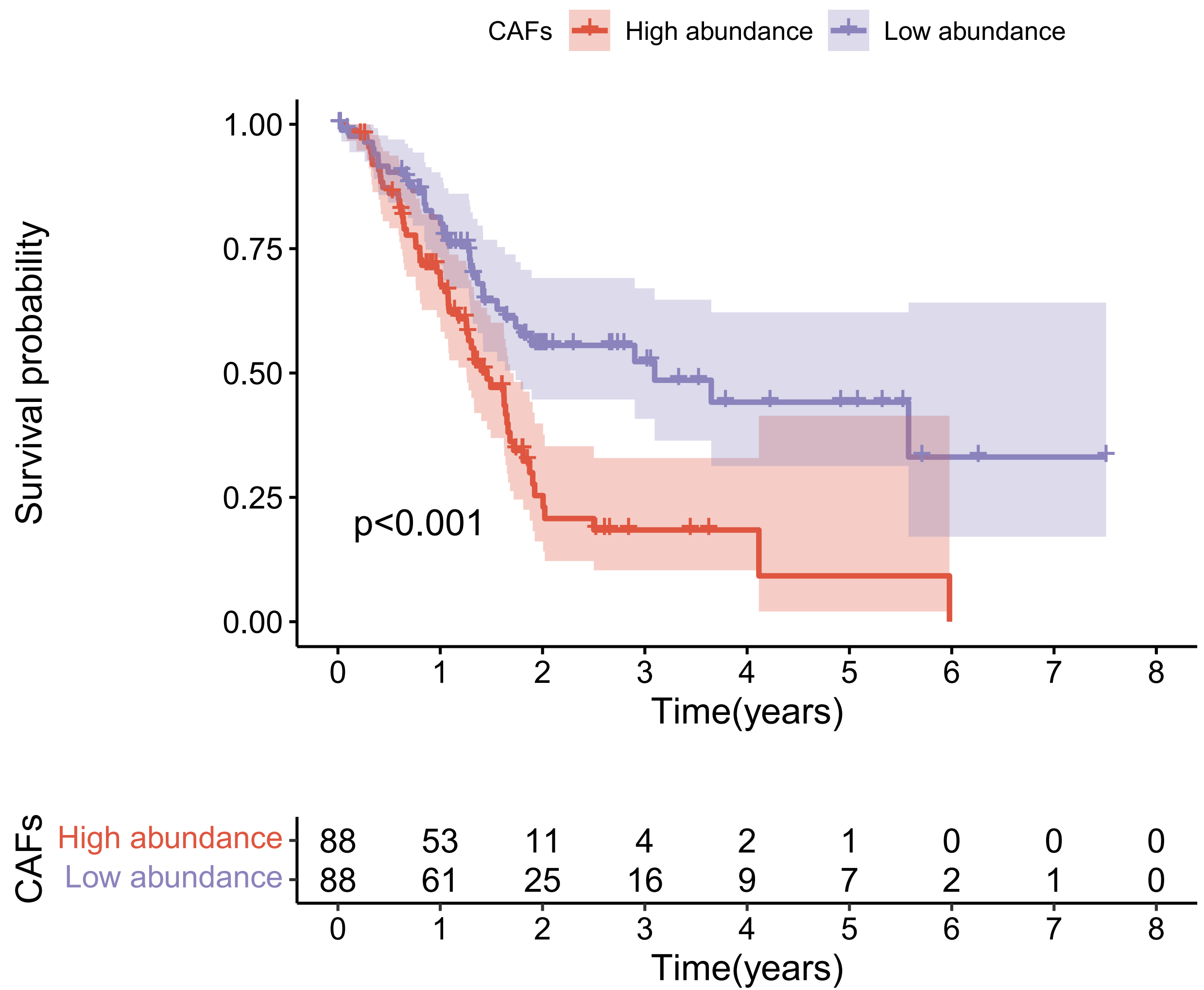
CAFs
High abundance
Low abundance
Survival probability
1.00
0.75
0.50
0.25
0.00
p<0.001
0
1
2
3
4
5
6
7
8
Time(years)
CAFs
High abundance 88 53 11 4 2 1 0 0 0
Low abundance 88 61 25 16 9 7 2 1 0
0
1
2
3
4
5
6
7
8
Time(years)
