# Supplementary material for: Integrated multiomics analysis identifies PHLDA1+ fibroblasts as prognostic biomarkers and mediators of biological functions in pancreatic cancer
Source: Front Immunol. 2025 Jul 4;16:1592416. doi: 10.3389/fimmu.2025.1592416 (PMC12271128; doi:10.3389/fimmu.2025.1592416)
Supplement: Supplementary file 7 [file DataSheet7.zip › raw data/2-cellchat/2.1_Mural_cells.survival.pdf]

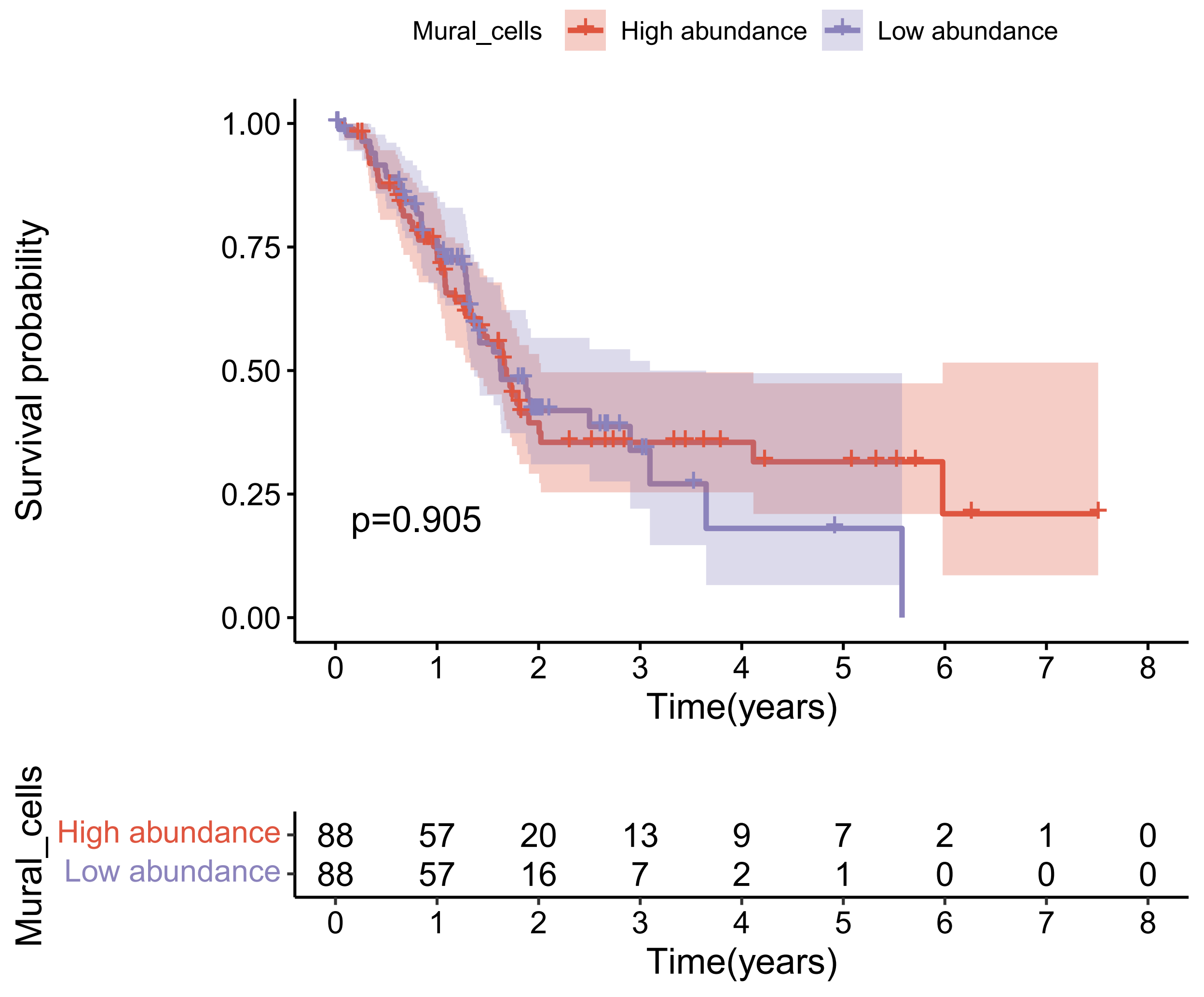

Mural_cells
High abundance
Low abundance
Survival probability
1.00
0.75
0.50
0.25
0.00
p=0.905
0 1 2 3 4 5 6 7 8
Time(years)
Mural_cells
High abundance 88 57 20 13 9 7 2 1 0
Low abundance 88 57 16 7 2 1 0 0 0
0 1 2 3 4 5 6 7 8
Time(years)
