# Supplementary material for: Integrated multiomics analysis identifies PHLDA1+ fibroblasts as prognostic biomarkers and mediators of biological functions in pancreatic cancer
Source: Front Immunol. 2025 Jul 4;16:1592416. doi: 10.3389/fimmu.2025.1592416 (PMC12271128; doi:10.3389/fimmu.2025.1592416)
Supplement: Supplementary file 8 [file Table1.docx]

**Table S1 Clinical characteristics of 30 patients with PDAC from Ruijin Hospital**

| **Characteristics** | **Number of cases** | **Percentages (%)** |
| --- | --- | --- |
| **Gender** | |  |
| Female | 13 | 43.3 |
| Male | 17 | 56.7 |
| **Age (years)** | |  |
| < 60 | 10 | 33.3 |
| ≥ 60 | 20 | 66.7 |
| **AJCC stage** | |  |
| I  II | 8  8 | 26.7  26.7 |
| III  IV | 8  6 | 26.7  20.0 |
| **T classification** | |  |
| T1 | 2 | 6.7 |
| T2 | 13 | 43.3 |
| T3 | 8 | 26.7 |
| T4 | 7 | 23.3 |
| **N classification** | |  |
| N0 | 12 | 40.0 |
| N1 | 9 | 30.0 |
| N2 | 9 | 30.0 |
| **M classification** | |  |
| M0 | 24 | 80.0 |
| M1 | 6 | 20.0 |
| **LN metastasis** | |  |
| Positive | 7 | 23.3 |
| Negative | 23 | 76.7 |
